# CHIMGEN: a Chinese imaging genetics cohort to enhance cross-ethnic and cross-geographic brain research

**DOI:** 10.1038/s41380-019-0627-6

**Published:** 2019-12-11

**Authors:** Qiang Xu, Lining Guo, Jingliang Cheng, Meiyun Wang, Zuojun Geng, Wenzhen Zhu, Bing Zhang, Weihua Liao, Shijun Qiu, Hui Zhang, Xiaojun Xu, Yongqiang Yu, Bo Gao, Tong Han, Zhenwei Yao, Guangbin Cui, Feng Liu, Wen Qin, Quan Zhang, Mulin Jun Li, Meng Liang, Feng Chen, Junfang Xian, Jiance Li, Jing Zhang, Xi-Nian Zuo, Dawei Wang, Wen Shen, Yanwei Miao, Fei Yuan, Su Lui, Xiaochu Zhang, Kai Xu, Long Jiang Zhang, Zhaoxiang Ye, Chunshui Yu, for the CHIMGEN Consortium

**Affiliations:** 1 Department of Radiology and Tianjin Key Laboratory of Functional Imaging, Tianjin Medical University General Hospital, 300052 Tianjin, China; 2 Department of Magnetic Resonance Imaging, The First Affiliated Hospital of Zhengzhou University, 450052 Zhengzhou, China; 3 Department of Radiology, Zhengzhou University People’s Hospital and Henan Provincial People’s Hospital, 450003 Zhengzhou, China; 4 Henan Key Laboratory for Medical Imaging of Neurological Diseases, 450003 Zhengzhou, China; 5 Department of Medical Imaging, The Second Hospital of Hebei Medical University, 050000 Shijiazhuang, China; 6 Department of Radiology, Tongji Hospital, Tongji Medical College, Huazhong University of Science and Technology, 430030 Wuhan, China; 7 Department of Radiology, Drum Tower Hospital, Medical School of Nanjing University, 210008 Nanjing, China; 8 Department of Radiology, Xiangya Hospital, Central South University, 410008 Changsha, China; 9 National Clinical Research Center for Geriatric Disorder, 410008 Changsha, China; 10 Department of Medical Imaging, The First Affiliated Hospital of Guangzhou University of Chinese Medicine, 510405 Guangzhou, China; 11 Department of Radiology, The First Hospital of Shanxi Medical University, 030001 Taiyuan, China; 12 Department of Radiology, The Second Affiliated Hospital of Zhejiang University, School of Medicine, 310009 Hangzhou, China; 13 Department of Radiology, The First Affiliated Hospital of Anhui Medical University, 230022 Hefei, China; 14 Department of Radiology, Yantai Yuhuangding Hospital, 264000 Yantai, China; 15 Department of Radiology, Tianjin Huanhu Hospital, 300350 Tianjin, China; 16 Tianjin Key Laboratory of Cerebral Vascular and Neurodegenerative Diseases, 300350 Tianjin, China; 17 Department of Radiology, Huashan Hosptial, Fudan University, 200040 Shanghai, China; 18 Functional and Molecular Imaging Key Lab of Shaanxi Province & Department of Radiology, Tangdu Hospital, The Military Medical University of PLA Airforce (Fourth Military Medical University), 710038 Xi’an, China; 19 Collaborative Innovation Center of Tianjin for Medical Epigenetics, Tianjin Key Laboratory of Medical Epigenetics, School of Basic Medical Sciences, Tianjin Medical University, 300070 Tianjin, China; 20 School of Medical Imaging, Tianjin Medical University, 300203 Tianjin, China; 21 Department of Radiology, Hainan General Hospital, 570311 Haikou, China; 22 Department of Radiology, Beijing Tongren Hospital, Capital Medical University, 100730 Beijing, China; 23 Department of Radiology, The First Affiliated Hospital of Wenzhou Medical University, 325000 Wenzhou, China; 24 Department of Magnetic Resonance, Lanzhou University Second Hospital, 730050 Lanzhou, China; 25 Department of Psychology, University of Chinese Academy of Sciences (CAS), 100049 Beijing, China; 26 CAS Key Laboratory of Behavioral Science, Institute of Psychology, 100101 Beijing, China; 27 Department of Radiology, Qilu Hospital of Shandong University, 250012 Jinan, China; 28 Department of Radiology, Tianjin First Center Hospital, 300192 Tianjin, China; 29 Department of Radiology, The First Affiliated Hospital of Dalian Medical University, 116011 Dalian, China; 30 Department of Radiology, Pingjin Hospital, Logistics University of Chinese People’s Armed Police Forces, 300162 Tianjin, China; 31 Department of Radiology, The Center for Medical Imaging, West China Hospital of Sichuan University, 610041 Chengdu, China; 32 Department of Radiology, The Second Affiliated Hospital and Yuying Children’s Hospital of Wenzhou Medical University, 325000 Wenzhou, China; 33 CAS Key Laboratory of Brain Function and Disease, University of Science and Technology of China, 230026 Hefei, China; 34 School of Life Sciences, University of Science & Technology of China, 230026 Hefei, China; 35 Department of Radiology, The Affiliated Hospital of Xuzhou Medical University, 221006 Xuzhou, China; 36 School of Medical Imaging, Xuzhou Medical University, 221004 Xuzhou, China; 37 Department of Medical Imaging, Jinling Hospital, Medical School of Nanjing University, 210002 Nanjing, China; 38 Department of Radiology, Tianjin Medical University Cancer Institute and Hospital, National Clinical Research Center for Cancer, Tianjin’s Clinical Research Center for Cancer, Key Laboratory of Cancer Prevention and Therapy, 300060 Tianjin, China; 39 CAS Center for Excellence in Brain Science and Intelligence Technology, Chinese Academy of Sciences, Shanghai, 200031 China

**Keywords:** Neuroscience, Psychiatric disorders

## Abstract

The Chinese Imaging Genetics (CHIMGEN) study establishes the largest Chinese neuroimaging genetics cohort and aims to identify genetic and environmental factors and their interactions that are associated with neuroimaging and behavioral phenotypes. This study prospectively collected genomic, neuroimaging, environmental, and behavioral data from more than 7000 healthy Chinese Han participants aged 18–30 years. As a pioneer of large-sample neuroimaging genetics cohorts of non-Caucasian populations, this cohort can provide new insights into ethnic differences in genetic-neuroimaging associations by being compared with Caucasian cohorts. In addition to micro-environmental measurements, this study also collects hundreds of quantitative macro-environmental measurements from remote sensing and national survey databases based on the locations of each participant from birth to present, which will facilitate discoveries of new environmental factors associated with neuroimaging phenotypes. With lifespan environmental measurements, this study can also provide insights on the macro-environmental exposures that affect the human brain as well as their timing and mechanisms of action.

## Introduction

Neuroimaging (intermediate) phenotypes reflecting the structural and functional properties of the human brain have been linked to human cognitive abilities and neuropsychiatric disorders (external phenotypes), and both intermediate and external phenotypes are precisely modulated by genetics, environments and their complex interactions [1, 2]. However, we know little about pathways from genetics and environments to neuroimaging phenotypes and then to external phenotypes. The associations between genetic factors and neuroimaging phenotypes have been investigated using neuroimaging genetics [3], initially by exploring the effects of a single nucleotide polymorphism (SNP) in small samples and eventually by identifying reliable genetic effects using genome-wide association studies (GWAS) in large samples [4, 5]. However, almost all available neuroimaging genetics cohorts include only Caucasian populations (Table 1), preventing us from identifying ethnic differences in genetic-neuroimaging associations. Although previous cohorts have included many micro-environmental factors, such as social economic status, early life events and lifestyle, few cohorts have included macro-environmental factors derived from remote sensing and national survey databases, such as climate, air pollution, population density, and gross domestic product (GDP) per capita. The joint analyses of micro- and macro-environmental variables will provide more information about environmental-neuroimaging associations and gene-environment interactions on neuroimaging phenotypes [6, 7]. Moreover, China has the largest populations in the world and has experienced dramatic changes in its macro-environments in recent decades, making the Chinese population more suitable for identifying macro-environmental factors associated with neuroimaging phenotypes.

**Table 1:** Comparisons of major neuroimaging genetics cohorts (*N* > 2000 with both genetic and neuroimaging data).

Project name	Number of subjects	Ethnic populations	Age range (year)	Diagnosis	Prominent features	Assessments of data quality^a^
CHIMGEN	*N* > 7000	Chinese Han only	18–30	Healthy	The largest prospective neuroimaging genetics cohort of Chinese Han adults with lifespan natural and socioeconomic environmental measurements obtained from remote sensing and national survey databases	Brain imaging data (+++): 3 modalities for all subjects; 2 for 2/3 subjects Genetic data (++++): genomic data Environment data (++++): more than a hundred of quantitative measures Behavioral data (++++): dozens of measures
UK Biobank	*N* > 30,000	Most Caucasian	40–69 at baseline	Mixed	The largest prospective longitudinal imaging genetics cohort of adults in the world	Brain imaging (++++): 5 imaging modalities for most subjects Genetic data (++++): genomic data Environment data (++): dozens of measures Behavioral data (++++): dozens of measures
ENIGMA	*N* > 50,000	Most Caucasian	3.3–91	Mixed	The largest imaging genetics pooling dataset included more than 50 currently available datasets with both imaging and genetic data	Brain imaging (++): only structural imaging for all subjects Genetic data (++++): genomic data Environment data (−): no measure Behavioral data (++): no measure but with diagnostic information
ABCD	*N* = 11,875	Most American African and Caucasian	9–10 at baseline	Relatively healthy	The largest prospective longitudinal imaging genetics cohort of children to explore adolescent brain development	Brain imaging (++++): 4 modalities for most subjects Genetic data (++++): genomic data Environment data (++): dozens of measures Behavioral data (++++): dozens of measures
IMAGEN	*N* = 2000	Most Caucasian	14–22	Relatively healthy	The first prospective longitudinal imaging genetics cohort of adolescence to investigate the risk for mental disorders	Brain imaging (++++): 4 modalities for most subjects Genetic data (++++): genomic data Environment data (++): dozens of measures Behavioral data (++++): dozens of measures
ADNI	*N* > 2000	Most Caucasian	55–90	AD, MCI and normal controls	The largest prospective longitudinal imaging genetics cohort of elderly people to define the progression of AD	Brain imaging (+++): 5 modalities but not collected from all subjects Genetic data (++++): genomic data Environment data (+): a few measures Behavioral data (++++):dozens of measures

*ABCD* Adolescent Brain Cognitive Development, *AD* Alzheimer’s disease, *ADNI* Alzheimer's Disease Neuroimaging Initiative, *CHIMGEN* Chinese Imaging Genetics, *ENIGMA* Enhancing Neuro Imaging Genetics Through Meta-Analysis, *IMAGEN* imaging genetics, *MCI* mild cognitive impairment

^a^Notes: The seven major imaging modalities included structural imaging, susceptibility weighted imaging (SWI), diffusion tensor imaging (DTI), artery spin labeling (ASL), task fMRI, resting-state fMRI, and positron emission tomography (PET). For each kind of data, the number of +signs indicates the subjective data availability, which includes two factors: the richness of the variables and the number of participants with data on these variables

Although the China Brain Project, which covers basic neuroscience, translational research, and brain-inspired intelligence, is being developed [8], there are no available large-scale Chinese neuroimaging genetics data. In this context, the Chinese Imaging Genetics (CHIMGEN) study was designed to collect genomic, environmental, neuroimaging, and behavioral data from a large number of Chinese participants to enhance neuroimaging genetics research in different ethnic populations and geographic locations. Compared with currently available large-scale neuroimaging genetics studies (Table 1), the CHIMGEN study includes the only cohort of non-Caucasian participants and has collected hundreds of macro-environmental measurements in addition to micro-environmental measurements. These comprehensive multiscale data can fill the gap in our understanding of how environmental factors, and their interaction with genetic factors can affect the human brain and consequently affect behavior by using effective dimension reduction or feature selection techniques [9–11].

## The CHIMGEN study

The CHIMGEN study (chimgen.tmu.edu.cn) was approved by the local ethics committee, and written informed consent was obtained from each participant. The aim of this study was to collect genomic, neuroimaging, environmental, and behavioral data from 10,000 healthy Chinese Han participants aged 18–30 years in 30 research centers from 21 mainland cities in China. To date, we have recruited more than 7000 participants, becoming the largest and most integrative Chinese neuroimaging genetics cohort. The detailed inclusion and exclusion criteria as well as the methods and procedures for screening; genotyping; blood sample collection; and behavioral, environmental, and neuroimaging data acquisition are described in the standardized operation procedures (SOPs) of the CHIMGEN study (Supplementary file 2). The detailed quality control procedures for personal information; blood samples; GWAS; and behavioral, environmental, and neuroimaging assessments are elaborated in the quality control manual of the CHIMGEN study (Supplementary file 3). Since the CHIMGEN study is ongoing, the following description of the CHIMGEN cohort was based on the data of only 5819 participants who had undergone comprehensive quality assessments.

### Sampling strategies

All participants were recruited by advisements posted in colleges and communities. The number of participants in each center depends on the available resources (researchers, funds, scanners, etc.) of the center. The recruited participants were not solely from the city or province of the participating centers. These samples are not used to represent populations (epidemiological samples), but to investigate biological mechanisms. Their epidemiological relevance needs to be investigated in subsequent studies.

### Recruitment distribution

**Fig. 1:**
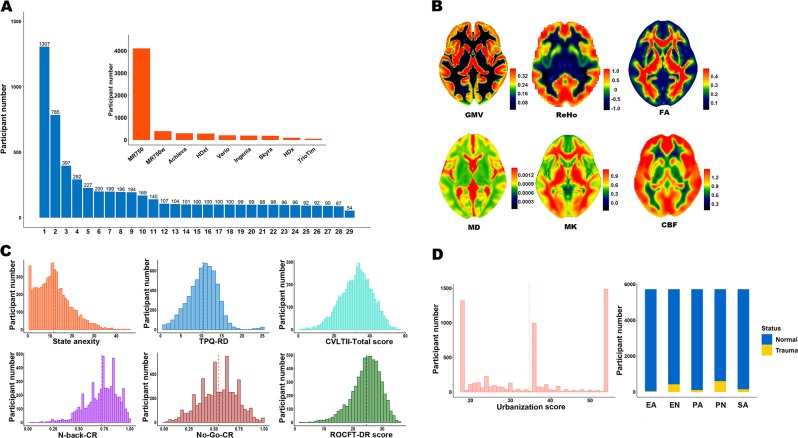
Recruitment and neuroimaging, behavioral, and environmental characteristics. **a** The main graph shows the numbers of participants recruited by each of the 29 centers. The insertion shows the numbers of participants recruited using each type of scanner. **b** The mean parameter maps of the gray matter volume (GMV), regional homogeneity (ReHo), fractional anisotropy (FA), mean diffusivity (MD), mean kurtosis (MK), and cerebral blood flow (CBF). **c** Data distribution of the representative behavioral assessments. CVLT II-Total score, the total number of correct recalls over the five learning trials of the word list A in the version 2 of the California verbal learning test; N-back-CR, the correct rate of the 3-back task in the N-back task; No-Go-CR, the correct rate of the No-Go task in the Go/ No-Go task; ROCFT-DR score, the score of delayed recall of the Rey-Osterrieth complex figure test; TPQ-RD, reward dependence of tridimensional personality questionnaire. **d** Data distribution of the representative paper-based environmental assessments. EA emotional abuse, EN emotional neglect, PA physical abuse, PN physical neglect, and SA sexual abuse.

The 5819 participants were recruited from 29 centers. The recruitment distribution of these participants across centers is shown in Fig. 1a. Eighteen of the 29 centers recruited more than 100 participants. The largest center recruited 1307 participants and the smallest center recruited 54 participants.

### Quality control for MR scanners

For each MR scanner, two phantoms were used to assess the imaging quality of the scanner. Specifically, an American College of Radiology MRI phantom was used to assess the functioning of the MR scanner, including geometric distortion, slice positioning and thickness accuracy, high contrast spatial resolution, intensity uniformity, ghosting artefacts and low contrast object detectability. A custom phantom [12, 13] was used to evaluate temporal stability during a functional MRI acquisition. Moreover, two healthy volunteers were scanned at all centers to assess the consistency of the MRI data acquired by different MR scanners. The effects of scanners on common MRI measures (gray matter volume (GMV), regional homogeneity (ReHo) and fractional anisotropy (FA)) are shown in Supplementary Fig. 1. These measures showed high consistency for MRI data acquired by the same type of MR scanner with the same scan parameters; however, there were visible differences for MRI data acquired by different types of MR scanners. For the latter, a meta-analysis of the results derived from MR data from different scanners may be a practical method to reduce the bias caused by MR scanner types.

### First-step quality assessments of the neuroimaging data

All 5819 participants were included in the first-step quality assessments of the neuroimaging data: 23 participants were excluded for metal artefacts, 1 for brain atrophy and 1 for excessively large ventricle. The remaining 5794 participants were included in the following quality control and statistics.

### Genotyping and quality control

A high-throughput genotyping chip designed for the Asian population (Illumina Asian screening array chip) with 700,000 sampling SNPs was used for genome-wide genotyping. Although all 5794 participants had blood samples, only 4885 participants have been genotyped thus far. After excluding two sex mismatching samples, nine duplicated or related samples, 29 samples with extreme heterozygosity and one sample with divergent ancestry (Supplementary Fig. 2), 4844 participants (99.16%) passed the quality control for the genetic data. It should be noted that the following quality assessments (*n* = 5753) also included 909 participants without genotyping results.

### Neuroimaging data and quality control

Neuroimaging data were acquired by nine types of 3.0-Tesla MRI scanner (Supplementary Fig. 3). Structural MRI (sMRI), diffusion tensor imaging (DTI) and resting-state functional MRI (rs-fMRI) data were acquired in all centers, and diffusion kurtosis imaging (DKI) and arterial spin labeling (ASL) data were acquired in 16 centers. The numbers of participants whose MRI data were acquired by each type of MRI scanner are shown in the insertion of Fig. 1a. The MRI data of 4045 (70.31%) of the 5753 participants were acquired by the MR 750 scanners. For each type of MRI scanner, the voxel-level maps of GMV calculated based on sMRI data, ReHo calculated based on rs-fMRI data, and FA and mean diffusivity (MD) calculated based on DTI data averaged across all qualified participants are shown in Supplementary Fig. 4. All types of scanner showed similar and symmetrical spatial distribution of the GMV, FA and MD, and 8/9 types of scanner showed similar and symmetrical spatial distribution of ReHo with the GE Signa HDx which showed asymmetric spatial distribution of the ReHo map, especially in posterior brain regions, being the only exception (Supplementary Fig. 4C). Therefore, the rs-fMRI data of the 97 participants acquired by the GE Signa HDx were excluded from this study.

The quality control results of the neuroimaging data (*n* = 5753) are shown in Supplementary Fig. 5. In the 5753 participants, there were 5743 (99.83%) participants with qualified sMRI data, 5507 (95.72%) with qualified rs-fMRI data, and 5750 (99.95%) with qualified DTI data. In the 3619 participants with DKI data, 3610 (99.75%) participants passed the quality control. In the 4108 participants with ASL data, all participants passed the quality control. Based on these MRI data, thousands of neuroimaging variables could be calculated. For example, the average maps of the GMV of the 5743 participants, the ReHo of the 5507 participants, the FA and MD of the 5750 participants, the mean kurtosis (MK) calculated based on DKI data of the 3610 participants, and the cerebral blood flow (CBF) calculated based on ASL data of the 4108 participants are shown in Fig. 1b. All of these parameter maps showed a symmetrical spatial distribution in the brain.

### Quality control for behavioral and paper-based environmental data

The preliminary quality control results for behavioral and paper-based environmental data of the 5753 participants are shown in Supplementary Fig. 6. In the 5753 participants, 8 participants were excluded for the loss of almost all behavioral and paper-based environmental data. In the remaining 5745 participants, 5723 (99.48%) participants with qualified Beck depression inventory (BDI- II) data, 5722 (99.46%) with qualified state and trait anxiety inventory (STAI) data, 5728 (99.57%) with qualified tridimensional personality questionnaire (TPQ) data, 5688 (98.87%) with qualified California verbal learning test (CVLT-II) data, 5619 (97.67%) with qualified symbol digit modalities test (SDMT) data, 5640 (98.04%) with qualified Rey-Osterrieth complex figure test (ROCFT) data, 5578 (96.96%) with qualified N-back task data, 5536 (96.23%) with qualified Go/No-Go task data, 5616 (97.62%) with qualified ball-tossing game data, 5639 (98.02%) with qualified ultimatum game (UG) data, 5733 (99.65%) with qualified urbanization score data, and 5728 (99.57%) with qualified childhood trauma questionnaire (CTQ) data.

The data distributions of the representative behavioral variables are demonstrated in Fig. 1c and those of the representative paper-based environmental variables are shown in Fig. 1d. Although some variables do not follow a normal distribution, the relatively wide range of values indicates good discriminative power across participants.

### Sample characteristics

The demographic characteristics of the 5745 participants with relatively complete assessments are shown in Table 2. This study included 3718 females and 2027 males. Their ages ranged from 18 to 30 years, with a mean ± standard deviation (SD) of 23.7 ± 2.4 years. Their years of education ranged from 9 to 24 years, with a mean ± SD of 16.8 ± 1.9 years. Their heights ranged from 146 to 197 cm, with a mean ± SD of 166.4 ± 7.9 cm. Their weights ranged from 23 to 114 kg, with a mean ± SD of 58.8 ± 10.7 kg. Their body mass indices (BMI) ranged from 10.8 to 38.5, with a mean ± SD of 58.8 ± 10.7. Most of these participants were unmarried (*n* = 5550), with only 195 married.

**Table 2:** Sex-specific demographic, behavioral and environmental data (*n* = 5745).

Items	Total (mean ± SD)	Males (mean ± SD)	Females (mean ± SD)	Gender differences		
				*Z/χ*² value	*P*-value	Effect size
Age (years)	23.7 ± 2.4 (*n* = 5745)	23.6 ± 2.6 (*n* = 2027)	23.7 ± 2.3 (*n* = 3718)	−2.77	5.58 × 10^−3^	−0.04
Years of education	16.8 ± 1.9 (*n* = 5745)	16.5 ± 1.9 (*n* = 2027)	16.9 ± 1.9 (*n* = 3718)	−8.18	2.74 × 10^−16^	−0.11
Marital status (No/Yes)	5550/195 (*n* = 5745)	1941/86 (*n* = 2027)	3609/109 (*n* = 3718)	6.88	8.73 × 10^−3^	0.03
Height (cm)	166.4 ± 7.9 (*n* = 5745)	174.5 ± 5.7 (*n* = 2027)	162.0 ± 5.0 (*n* = 3718)	−56.35	<1.15 × 10^−190^	−0.74
Weight (Kg)	58.8 ± 10.7 (*n* = 5745)	68.5 ± 10.1 (*n* = 2027)	53.6 ± 6.6 (*n* = 3718)	−51.12	<1.15 × 10^−190^	−0.67
BMI	21.1 ± 2.6 (*n* = 5745)	22.5 ± 2.8 (*n* = 2027)	20.4 ± 2.2 (*n* = 3718)	−29.45	1.15 × 10^−190^	−0.39
TPQ-NS	13.8 ± 4.5 (*n* = 5728)	13.0 ± 4.3 (*n* = 2019)	14.2 ± 4.5 (*n* = 3709)	−9.48	2.66 × 10^−21^	−0.13
BDI-II	3.37 ± 4.41 (*n* = 5723)	3.01 ± 4.06 (*n* = 2017)	3.56 ± 4.58 (*n* = 3706)	−4.25	2.19 × 10^−5^	−0.06
State anxiety	30.8 ± 7.6 (*n* = 5722)	30.2 ± 7.5 (*n* = 2018)	31.2 ± 7.6 (*n* = 3704)	−5.39	7.20 × 10^−8^	−0.07
CVLT II-total score	55.6 ± 8.8 (*n* = 5703)	54.1 ± 9.1 (*n* = 2014)	56.4 ± 8.6 (*n* = 3689)	−9.06	1.32 × 10^−19^	−0.12
ROCFT-DR score	24.7 ± 5.0 (*n* = 5643)	24.8 ± 5.0 (*n* = 1989)	24.6 ± 5.0 (*n* = 3654)	−1.46	0.15	−0.02
SDMT score	69.6 ± 12.7 (*n* = 5619)	68.8 ± 13.1 (*n* = 1989)	70.0 ± 12.5 (*n* = 3630)	−4.12	3.76 × 10^−5^	−0.05
N-back-CR	0.73 ± 0.16 (*n* = 5578)	0.74 ± 0.15 (*n* = 1973)	0.72 ± 0.16 (*n* = 3605)	−5.88	4.18 × 10^−9^	−0.079
No-Go-CR	0.55 ± 0.19 (*n* = 5536)	0.58 ± 0.19 (*n* = 1957)	0.53 ± 0.18 (*n* = 3579)	−10.05	9.48 × 10^−24^	−0.14
UG-AR	0.76 ± 0.32 (*n* = 5639)	0.78 ± 0.31 (*n* = 1989)	0.74 ± 0.33 (*n* = 3650)	−4.08	4.54 × 10^−5^	−0.05
BTG-ACC	0.80 ± 0.19 (*n* = 5616)	0.81 ± 0.19 (*n* = 1977)	0.79 ± 0.19 (*n* = 3639)	−3.82	1.34 × 10^−4^	−0.05
Urbanization score	34.7 ± 13.9 (*n* = 5733)	35.2 ± 14.0 (*n* = 2022)	34.5 ± 13.9 (*n* = 3711)	−1.91	0.06	−0.03
CTQ-EN	8.19 ± 4.17 (*n* = 5728)	8.01 ± 3.98 (*n* = 2022)	8.28 ± 4.26 (*n* = 3706)	−1.45	0.15	−0.02

Notes: Only one representative measure of each behavioral or environmental assessment is shown in this table. The sample sizes of the behavioral and environmental assessments are different across measures because only qualified participants are included in the statistical analysis. The effect sizes for categorical variables are evaluated by *Φ* and those for continuous variables are evaludated with Mann–Whitney and Wilcoxon nonparametric tests using *r*. Cohen’s guidelines for effect size are that a large effect is >0.5, a medium effect is between 0.3 and 0.5, and a small effect is between 0.1 and 0.3

*BDI-II* beck depression inventory II, *BMI* body mass index, *BTG-ACC* the total correct rate in the ball-tossing game, *CTQ-EN* the emotional neglect score of childhood trauma questionnaire, *CVLT II-Total score* the total number of correct recalls over the five learning trials of the word list A in the version 2 of the California verbal learning test, *N-back-CR* the correct rate of the 3-back task in the N-back task, *No-Go-CR* the correct rate of the No-Go task in the Go/ No-Go task, *ROCFT-DR score* the score of delayed recall of the Rey-Osterrieth complex figure test, *SDMT* symbol digit modalities test, *TPQ-NS* novelty-seeking of tridimensional personality questionnaire, *UG-AR* the ratio of participants who accept the 1:9 allocation schemes in situation 1 of the ultimate game. In this situation, if the participant accepts the plan, the proposer and the participant will divide the money according to this plan. If the participant rejects the plan, neither of them gets the money

### Sex-specific demographic, behavioral, and paper-based environmental statistics

The sex-specific demographic, behavioral and paper-based environmental statistics of the 5745 participants with relatively complete assessments are shown in Table 2. Although most of these variables show significant differences (*P* < 0.05) between male and female participants, the effect sizes were generally very small except for sex differences in height (|*r*| = 0.74, large effect), weight (|*r*| = 0.67, large effect), and BMI (|*r*| = 0.39, medium effect).

### Quantitative environmental variables derived from remote sensing and national survey databases

**Fig. 2:**
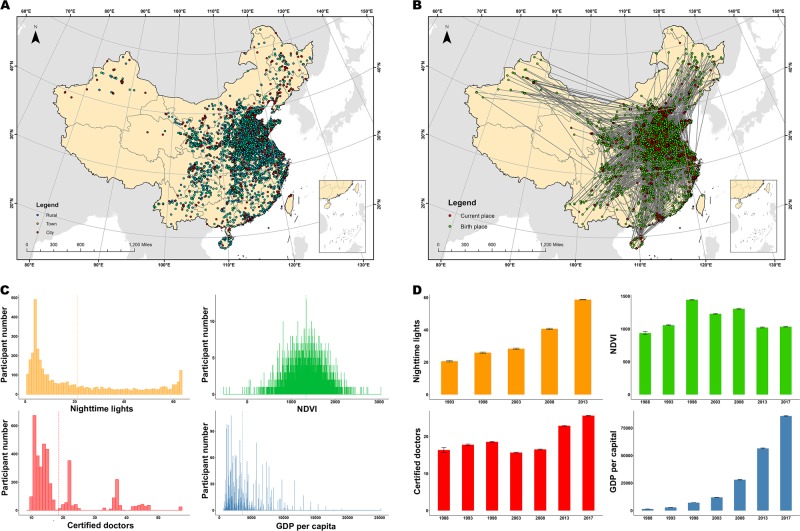
Environmental variables derived from remote sensing and national survey data. **a** Geographic location of each participant’s birthplace (*n* = 5723). Blue dots indicate rural area, green dots indicate towns, and red dots indicate cities. **b** The migration map of participants (*n* = 3979). Red dots indicate current places of residence, and green dots indicate birthplaces. Gray lines connect the birthplaces and current places of residence of a given participant. **c** Data distribution of the representative environmental variables in the birth year or the year nearest to the birth year. Certified doctors is the number of certified doctors per 10,000 persons. NDVI, normalized difference vegetation index, and GDP, gross domestic product. **d** Longitudinal changes of the representative environmental variables in selected years. The value in each column is shown as the mean ± SE.

In this study, we recorded the precise residential location of each participant in each year from birth to present. In the 5745 participants who passed the initial quality controls for the neuroimaging, behavioral and genetic data, 5723 participants (99.62%) provided both current and birthplace (Fig. 2a) residential locations; however, only 3979 participants (69.26%) provided lifetime migration information (Fig. 2b). Based on remote sensing and national survey databases, we obtained hundreds of macro-environmental measurements for each participant. Some representative macro-environmental variables at birth (Fig. 2c) and their lifetime changes are shown in Fig. 2d.

### Future plans of the CHIMGEN study

In the future, the CHIMGEN consortium will complete the following tasks: (a) further recruit at least 3000 participants to reach the goal of 10,000 qualified participants; (b) simultaneously obtain the genomic, epigenomic, and transcriptomic data of ~700 participants; (c) collect 2000–3000 patients with major mental disorders; and (d) develop the CHIMGEN cohort into a longitudinal cohort by recalling the participants at a later time.

### Data sharing policy

We would like to share all CHIMGEN data (including the genetic, environmental, neuroimaging and behavioral data) with other scientific communities according to the laws and regulations of the Chinese government. All the raw data of the CHIMGEN study can be accessed via collaboration with the CHIMGEN consortium. The summary statistics of the CHIMGEN data can be freely accessed via a formal application. A detailed scheme for sharing the CHIMGEN data can be found on our website (chimgen.tmu.edu.cn) and in Supplementary file 4.

## Discussion

With genomic, environmental, neuroimaging, and behavioral data, the CHIMGEN study will help answer the following scientific questions about the associations between genetic and environmental factors on one hand and brain and cognitive phenotypes on the other hand.

### Cross-ethnic differences in genetic-neuroimaging associations

Although GWAS analyses have identified many genetic variants associated with cognitive and neuropsychiatric phenotypes [14–16], we know little about the genetic variants associated with neuroimaging phenotypes. The most substantial obstacle for neuroimaging genetics studies is the time and economic cost of collecting high-quality neuroimaging data in a large sample (e.g., 10,000 participants). Fortunately, European and American countries have launched several large-scale neuroimaging genetics studies (*n* > 2000) (Table 1), such as the Alzheimer Disease Neuroimaging Initiative (ADNI) [17, 18], Imaging Genetics (IMAGEN) [19], Enhancing Neuroimaging Genetics through Meta-analysis (ENIGMA) [20], UK Biobank (UKBB) [21], and Adolescent Brain Cognitive Development (ABCD) [22]. These studies aim to identify reliable genetic variants associated with neuroimaging phenotypes and to discover new biomarkers for neuropsychiatric disorders. However, the majority of the participants included in these cohorts are Caucasian.

Ethnic differences have been reported in the allele frequencies of SNPs [23–25], linkage disequilibrium and polygenic risk scores [26], genetic susceptibilities for neuropsychiatric disorders [27], and neuroimaging phenotypes [28–30]. In addition to environmental factors, genetic factors are the main causes for ethnic differences in neuroimaging phenotypes because of their high heritability [31–33]. However, the common and specific genetic variants associated with neuroimaging phenotypes of different ethnic populations remain unknown, because there is no available large-scale neuroimaging genetics cohort of non-Caucasian individuals. From this perspective, the CHIMGEN data will provide an opportunity to discover ethnic differences in neuroimaging-related genetic variants between Chinese and Caucasian participants.

Although it is clinically important to identify genetic associations with neuroimaging markers of neuropsychiatric disorders [34–38], it is also critical to identify genetic-neuroimaging associations in normal populations to better understand how genetic variants cause brain structural and functional impairments in neuropsychiatric disorders. However, none of the large-scale neuroimaging genetics studies (*n* > 2000) have included a sufficient number of healthy adults aged 18–30 years (Table 1), an age window during which human brains and their functions are minimally influenced by the confounding factors of development and ageing [39]. Thus, the CHIMGEN study of 7000 healthy adults between 18–30 years is suitable for investigating genetic-neuroimaging associations in unaged mature brains.

### Environmental factors associated with neuroimaging phenotypes

One unique aspect of the CHIMGEN study is the collection of hundreds of macro-environmental measurements from satellite images and national survey databases. Compared with micro-environmental assessments based on questionnaire and self-report data, remote sensing, and national survey data can provide many new quantitative macro-environmental assessments. For example, we can obtain quantitative environmental measurements of landform and topography, urbanization, climate, and air quality of the living places of each participant based on remote sensing data [40–43], and those of economy, urbanization, living condition, healthcare, and education of the living places of the participant based on national survey databases (data.stats.gov.cn/english/). Associations between neuroimaging phenotypes and most of these macro-environmental measurements have not been explored, and they may provide us with an opportunity to discover new environmental factors related to neuroimaging phenotypes. The feasibility of using macro-environmental measurements derived from remote sensing and national survey databases to discover new environmental factors associated with the human brain and behavioral phenotypes has been tested in pilot studies. For example, the green space assessed by the normalized difference vegetation index (NDVI) based on remote sensing data has been linked to human health [44, 45], and the lifelong exposure to greenness has been associated with GMV differences in children [7]. In addition, several macro-environmental measurements derived from national survey databases, such as population density, local GDP per capita, medical supply, and educational resources have also been associated with human health [46–48].

More importantly, with the precise lifelong residential locations of each participant, we can obtain the macro-environmental measurements of each participant in each year from birth to present, from which we can estimate the cumulative exposure of environmental risk factors throughout the lifespan or during a period of interest. The detailed lifelong environmental data of the CHIMGEN study will help determine the macro-environmental exposures that affect the structural and functional properties of the human brain as well as their timing and mechanisms of action.

### Genome-wide by environment interactions on neuroimaging phenotypes

Most neuropsychiatric disorders have a multifactorial etiology and emerge through the interplay of genetic and environmental factors [49]. Similarly, the structural and functional architectures of the human brain are also modulated by both factors [50], and gene-environment interactions may explain the missing heritability of certain phenotypes [51]. Candidate-gene approaches have been used extensively to explore gene-environment interactions. For example, the serotonin transporter promoter polymorphism interacts with stressful life events to increase the risk of depression [52]. However, candidate-gene approaches are criticized for oversimplifying the genetic substrates of these complex phenotypes since a single genetic variant minimally contributes to these phenotypes. The PRS integrates many genetic variants of the genome and is a better representation of genetic risk than single variants by having a much larger effect [53, 54]. Indeed, considering the combination of PRS and childhood trauma can improve the ability to predict depression [55, 56]. Genome-wide by environment interactions have been used to unbiasedly explore the effects of gene-environment interactions on depression [57]. However, the lack of large dataset simultaneously with genome-wide genetic data, objective environmental assessments and neuroimaging data has prevented investigations of genome-wide by environment interactions on neuroimaging phenotypes. In this context, the CHIMGEN study has rich genomic, environmental, and neuroimaging measurements of 7000 participants, and is particularly suited to investigate genome-wide by environment interactions on human neuroimaging phenotypes.

### Gene (environment)-brain-behavior pathways

In contrast to many studies focusing on pairwise correlations of genetic variants, environmental factors, neuroimaging measures, and cognitive or neuropsychiatric phenotypes, only a few studies have explored biological pathways from genes and environment to brain structure and function and ultimately to cognition and symptoms [58–60]. These studies have been primarily conducted using candidate-gene approaches and small samples, and they have been criticized based on the minimal effect size of a single variant and their lack of statistical power. In view of polygenic profiles of neuroimaging and cognitive phenotypes [61, 62], genomic data should be integrated to identify normal and abnormal gene-brain-behavior pathways. Since environmental factors alone and gene-environment interactions affect neuroimaging and cognitive phenotypes [6, 63, 64], it is important to identify the environmental factors associated with these phenotypes, which would help better guide clinical practice to address these adverse environmental factors. Furthermore, it is also critical to investigate how gene-environment interactions affect brain structure and function and then influence normal cognitive functions and brain disorders. By gathering genomic, environmental, neuroimaging, and cognitive data, the CHIMGEN project is ideally suited to explore the normal pathways of gene (environment)-brain-cognition.

### Comprehensive understanding of human cognitive functions with multiscale data

The human brain is the most complex system in the world, and even the simplest cognitive task requires an efficient cooperation of multiscale neural elements [65, 66]. Thus, human cognitive function can be understood only by integrating multimodal data at different scales, e.g., genomic, epigenomic, transcriptomic, and proteomic data at the microscale, neural circuit, and neuronal activity data at the mesoscale, and neuroimaging data at the macroscale. In addition to establishing reliable correlations between multiscale features and cognitive functions, it is also critical to identify causal linkages between these features to discover the causal pathways from the microscale to the mesoscale then to the macroscale and ultimately to cognition [8]. With genomic, transcriptomic, epigenomic, neuroimaging, and cognitive data obtained from 700 participants, the CHIMGEN study can be used to establish associations between microscale genetic variants and macroscale neuroimaging phenotypes, and then the functions of the identified genetic variants can be explored and validated at the cellular level [67] and in animal models [68] using gene editing techniques. One can also try to identify causal links among findings from different scales by integrating currently available multiscale neurobiological datasets and state-of-the-art bioinformatics.

### Associations with major neuropsychiatric disorders

Many neuropsychiatric disorders are associated with genetic and environmental factors and their interactions [69]. We have identified many risk factors for major neuropsychiatric disorders, but the underlying mechanisms remain largely unknown. Taking neuroimaging measures as intermediate phenotypes, researchers could explore how these factors increase the risk for neuropsychiatric disorders by investigating the effects of these factors on neuroimaging measures in healthy subjects. For example, the CHIMGEN data can be used to investigate the effects of a single or integrated genetic and/or environmental risk factor(s) for neuropsychiatric disorders on neuroimaging phenotypes in healthy individuals. Moreover, we can identify new genetic or environmental risk factors that significantly affect neuroimaging markers of neuropsychiatric disorders.

### Potential models, methods or strategies for analyzing the CHIMGEN data

Many models, methods and strategies can be used to analyze the CHIMGEN data. For example, GWAS can identify genetic variants associated with neuroimaging phenotypes [4, 70, 71], multifactor dimensionality reduction and derivatives can investigate genome-wide gene-gene interactions on these phenotypes [72–74], and canonical correlation and partial least square regression analyses can uncover environmental factors associated with these phenotypes [75, 76]. Although genome-wide gene-environment interaction studies theoretically need more samples than GWAS, the CHIMGEN data can be used to investigate gene-environment interactions on neuroimaging phenotypes with effective dimension reduction or feature selection techniques [77–79]. For example, a structured linear mixed model was recently proposed to identify candidate loci that interact with environmental variables [80]. The linkage disequilibrium score regression can estimate genetic correlations of neuroimaging phenotypes with disease-, personality- or cognition-related phenotypes [81]. Mendelian randomization and mediation analysis [82] can identify potential pathways from genes to brain to cognition. Artificial intelligence techniques, such as deep learning algorithms [83], can disclose meaningful relationships between measures from different scales.

## Conclusion

As an important supplement to the research field of neuroimaging genetics, the CHIMGEN cohort can be integrated with cohorts of different ethnicities, geographic locations and socioeconomic conditions to facilitate a cross-ethnic and cross-geographic understanding of the human brain. By integrating these cohorts, we can identify the effect of ethnic factors on the brain by controlling for or stratifying by geographic and socioeconomic factors. With the same strategies, we can identify common and specific genetic-neuroimaging associations in various ethnic populations. More importantly, we can identify brain-related macro- and micro-environmental factors that are common to all ethnic populations or specific to a certain ethnic population. Therefore, cross-ethnic and cross-geographic studies based on integrated cohorts would enhance our understanding of how human brains differ from each other.

## Supplementary information

Supplementary File 1

Supplementary File 2

Supplementary File 3

Supplementary File 4

Supplementary Figure 1

Supplementary Figure 2

Supplementary Figure 3

Supplementary Figure 4

Supplementary Figure 5

Supplementary Figure 6

Supplementary Figure Legends
