# Supplementary material for: CHIMGEN: a Chinese imaging genetics cohort to enhance cross-ethnic and cross-geographic brain research
Source: Mol Psychiatry. 2019 Dec 11;25(3):517–29. doi: 10.1038/s41380-019-0627-6 (PMC7042768; doi:10.1038/s41380-019-0627-6)
Supplement: Supplementary file 1 [file 41380_2019_627_MOESM1_ESM.pdf]

CHIMGEN Consortium information

The members of the CHIMGEN Consortium are as follows:

Department of Radiology and Tianjin Key Laboratory of Functional Imaging, Tianjin Medical University General Hospital

Chunshui Yu; Quan Zhang; Wen Qin; Feng Liu; Junping Wang; Qiang Xu; Jiayuan Xu; Xue Zhang; Xinjun Suo; Jilian Fu; Congcong Yuan; Yuan Ji; Hui Xue; Tianying Gao; Junpeng Liu; Yanjun Li; Xi Guo; Lixue Xu; Jiajia Zhu; Huaigui Liu; Fangshi Zhao; Jie Sun; Yongjie Xu; Huanhuan Cai; Jie Tang; Yaodan Zhang; Yongqin Xiong; Xianting Sun; Nannan Pan; Xue Zhang (Junior); Jiayang Yang; Nana Liu; Ya Wen; Dan Zhu; Bingjie Wu; Wenshuang Zhu; Qingqing Diao; Yujuan Cao; Bingbing Yang; Lining Guo; Yingying Xie; Jiahui Lin; Zhimin Li; Yan Zhang; Kaizhong Xue; Zirui Wang; Junlin Shen

Collaborative Innovation Center of Tianjin for Medical Epigenetics, Tianjin Key Laboratory of Medical Epigenetics, School of Basic Medical Sciences, Tianjin Medical University

Mulin Jun Li; Shijie Zhang

School of Medical Imaging, Tianjin Medical University

Meng Liang; Xuejun Zhang; Hao Ding; Qian Su

Department of Magnetic Resonance Imaging, The First Affiliated Hospital of Zhengzhou University

Jingliang Cheng; Caihong Wang; Peifang Miao; Fuhong Duan; Yafei Guo; Weijian Wang

Department of Radiology, Zhengzhou University People's Hospital and Henan Provincial People's Hospital

Meiyun Wang; Dapeng Shi; Lun Ma; Yan Bai; Min Guan; Wei Wei

Department of Medical Imaging, The Second Hospital of Hebei Medical University

Zuojun Geng; Yuzhao Wang; Yaikai Wu; Xuran Feng; Ling Li; Duo Gao

Department of Radiology, Tongji Hospital, Tongji Medical College, Huazhong University of Science and Technology

Wenzhen Zhu; Tian Tian

Department of Radiology, Drum Tower Hospital, Medical School of Nanjing University

Bing Zhang; Zhao Qing; Sichu Wu; Junxia Wang; Yi Sun; Yang He

Department of Radiology, Xiangya Hospital, Central South University

Weihua Liao; Shuai Yang; Youming Zhang

Department of Medical Imaging, The First Affiliated Hospital of Guangzhou University of Chinese Medicine

Shijun Qiu; Yi Liang; Yujie Liu; Hui Zeng; Jingxian Chen

Department of Radiology, The First Hospital of Shanxi Medical University

Hui Zhang; Xiaochun Wang; Ying Lei

Department of Radiology, The Second Affiliated Hospital of Zhejiang University, School of Medicine

Xiaojun Xu; Jingjing Xu; Xiaojun Guan

Department of Radiology, The First Affiliated Hospital of Anhui Medical University

Yongqiang Yu; Xiaohu Li; Xiaoshu Li

Department of Radiology, Yantai Yuhuangding Hospital

Bo Gao; Gang Zhang; Kang Yuan

Department of Radiology, Tianjin Huanhu Hospital

Tong Han; Jun Guo; Hao Lu

Department of Radiology, Huashan Hosptial, Fudan University

Zhenwei Yao; Yue Wu

Functional and Molecular Imaging Key Lab of Shaanxi Province & Department of Radiology, Tangdu Hospital, the Military Medical University of PLA Airforce (Fourth Military Medical University)

Guangbin Cui; Wen Wang; Linfeng Yan; Yang Yang; Jin Zhang

Department of Radiology, Hainan General Hospital

Feng Chen; Yuankai Lin; Hui Juan Chen

Department of Radiology, Beijing Tongren Hospital, Capital Medical University

Junfang Xian; Qian Wang; Xiaoxia Qu; Ying Wang

Department of Radiology, The First Affiliated Hospital of Wenzhou Medical University

Jiance Li; Yunjun Yang; Nengzhi Xia

Department of Magnetic Resonance, Lanzhou University Second Hospital

Jing Zhang; Guangyao Liu; Laiyang Ma

Department of Psychology, University of Chinese Academy of Sciences

Xi-Nian Zuo; Zhe Zhang; Yin-Shan Wang; Quan Zhou

Department of Radiology, Qilu Hospital of Shandong University

Dawei Wang; Li Hu; Jizhen Li

Department of Radiology, Tianjin First Center Hospital

Wen Shen; Miaomiao Long; Lihua Liu

Department of Radiology, The First Affiliated Hospital of Dalian Medical University

Yanwei Miao; Weiwei Wang; Yujing Zhou

Department of Radiology, Pingjin Hospital, Logistics University of Chinese People's Armed Police Forces

Fei Yuan; Quan Zhang

Department of Radiology, the Center for Medical Imaging, West China Hospital of Sichuan University;

Su Lui

Department of Radiology, The Second Affiliated Hospital and Yuying Children's Hospital of Wenzhou Medical University

Zhihan Yan; Yuchuan Fu; Yi Lu

CAS Key Laboratory of Brain Function and Disease, University of Science and Technology of China

Xiaochu Zhang; Rujing Zha; Ying Li; Lizhuang Yang; Ying Chen; Ling Zuo

Department of Radiology, The Affiliated Hospital of Xuzhou Medical University;

Kai Xu; Haitao Ge; Peng Xu; Cailuan Lu; Chen Wu; Xiaoying Yang

Department of Medical Imaging, Jinling Hospital, Medical School of Nanjing University

Long Jiang Zhang; Li Juan Zheng; Li Lin; Yun Fei Wang; Han Zhang; Xin Yuan Zhang

Department of Radiology, Tianjin Medical University Cancer Institute and Hospital, National Clinical Research Center for Cancer, Tianjin's Clinical Research Center for Cancer, Key Laboratory of Cancer Prevention and Therapy

Zhaoxiang Ye; Peng Zhang; Wei Li
