# Supplementary material for: CHIMGEN: a Chinese imaging genetics cohort to enhance cross-ethnic and cross-geographic brain research
Source: Mol Psychiatry. 2019 Dec 11;25(3):517–29. doi: 10.1038/s41380-019-0627-6 (PMC7042768; doi:10.1038/s41380-019-0627-6)
Supplement: Supplementary file 2 [file 41380_2019_627_MOESM2_ESM.pdf]

Standard Operating Procedures

(SOPs)

The CHIMGEN consortium

Content

Chapter 1 Overview…………..………………………………………...……………3

Chapter 2 Screening……………..……………………………………………...………9

Chapter 3 Behavioral and environmental assessments..…………………….....…..…26

Chapter 4 Quantitative environmental assessments..………………………..………50

Chapter 5 Blood samples………...………………......…………..……………..………57

Chapter 6 Genotyping..……………...……………….…….…….……………………71

Chapter 7 Neuroimaging data.………………….…………………….…………….77

Chapter 1 Overview

1. Background

With the availability of the large-sample neuroimaging genetics cohorts launched by Europe and North America, such as the Alzheimer Disease Neuroimaging Initiative (ADNI), Imaging Genetics (IMAGEN), Enhancing Neuroimaging Genetics through Meta-Analysis (ENIGMA), Human Connectome Project (HCP), UK Biobank (UKBB) and Adolescent Brain Cognitive Development (ABCD) study, researchers have started to use genome-wide association studies (GWAS) to unbiasedly identify genetic variants associated with neuroimaging phenotypes. However, few non-Caucasian participants have been included in these cohorts. It has been suggested that there are great ethnic differences in allele frequencies of single nucleotide polymorphisms (SNPs), linkage disequilibrium (LD) and polygenic risk scores (PRS), genetic susceptibilities for neuropsychiatric disorders, and structural and functional properties of the human brain. Although environmental factors may result in ethnic differences in neuroimaging phenotypes, genetic factors are thought to be the main contributors because the human brain architecture has a rather high heritability. Nevertheless, the common and specific genetic variants associated with inter-individual differences in brain structural and functional properties between different ethnic populations remain largely unknown, because there are no available large non-Caucasian cohorts of neuroimaging genetics data. From this perspective, a large sample of neuroimaging genetics data of Chinese will provide an opportunity to investigate common and specific genetic variants associated with neuroimaging phenotypes between Chinese and Caucasian participants.

In addition to genetic factors contributing to inter-individual differences in neuroimaging phenotypes, in the past four decades, Chinese people have experienced dramatic changes in both natural and socioeconomic environments, which may provide an opportunity to identify major environmental factors associated with structural and functional architectures of the human brain. In most of previous studies,

environmental information was collected by questionnaires or self-reports that are highly subjective. Therefore, more objective and quantitative environmental assessments are needed to identify environmental factors that are associated with neuroimaging phenotypes. For example, the precise residential location in each year from birth to present can be recorded for each participant, and consequently longitudinal natural and socioeconomic environmental data of each participant can be obtained from remote sensing satellite images and national survey databases. These objective and quantitative environmental assessments can provide valuable insights on what, when and how environmental factors affect neuroimaging phenotypes on a large scale in space and time.

2. The CHIMGEN study

In 2014, we began to design the Chinese Imaging Genetics (CHIMGEN) study, which aimed to create a large-sample Chinese neuroimaging genetics cohort, including genomic, environmental, neuroimaging and behavioral data, to enhance cross-ethnic and cross-geographic brain research. Before the start of the project, investigators were trained to understand the concept of neuroimaging genetics, research aims, inclusion and exclusion criteria, experimental pipeline, and standard operating procedures (SOPs) for acquiring genetic, environmental, neuroimaging and behavioral data. The experiment was performed in the order of the following procedures: screening, behavioral and environmental assessments, brain magnetic resonance imaging (MRI) scans, and blood sample collection.

In the screening session, all participants were recruited by advertisement and satisfied the inclusion criteria of Chinese Han, aged 18-30 years and right handedness. Participants were excluded if they met any of the following criteria: abnormal color discrimination, regular smoker, pregnancy, MRI contraindications, and a history of alcohol or drug abuse, neuropsychiatric or severe somatic disorder, currently any medication, sedative-hypnotic medication within a month or any medication for major neuropsychiatric disorders. Participants were also excluded if their relatives had been included in the study or had a history of psychiatric disorders. The mini-international

neuropsychiatric interview (MINI), the Chinese edition of the Edinburgh handedness inventory and the alcohol use disorders identification test (AUDIT) were used in the process of including and excluding participants. Only qualified participants would receive blood sample collection, brain MRI examination, behavioral and environmental assessments.

In the session of behavioral and environmental assessments, we evaluated verbal memory using the California verbal learning test (CVLT-II), spatial memory using the Rey-Osterrieth (RO) complex figure test (ROCFT), working memory using the N-back test, executive function using the Go/No-Go task, fair decision making using the ultimatum game, information processing speed using the symbol digit modalities test (SDMT), and perspective taking using the ball tossing game. In addition, we also used the state and trait anxiety inventory (STAI) and the Beck depression inventory (BDI-II) to estimate anxiety and depression and used the tridimensional personality questionnaire (TPQ) to assess personality. As for environmental assessments, the environmental questionnaires were used to assess urbanization and early life adversity. Moreover, we recorded the precise residential location of each participant in each year from birth to present which could be used to obtain hundreds of longitudinal natural and socioeconomic environmental assessments for each participant based on remote sensing satellite images and national survey databases.

In neuroimaging session, brain MRI data were acquired by 3.0-Tesla scanners from General Electrics, Siemens and Philips. The acquisition parameters were designed by experienced technicians and radiologists to ensure relatively consistent imaging quality across different scanners. The high-resolution structural magnetic resonance imaging (sMRI), diffusion tensor imaging (DTI) and resting-state functional MRI (rs-fMRI) data were acquired in all centers, and diffusion kurtosis imaging (DKI) and arterial spin labeling (ASL) data were acquired in more than a half of the centers. These MRI modalities were designed to assess different structural and functional properties of the human brain. The sMRI was used to assess volume, thickness and surface area of the cerebral cortex; rs-fMRI was used to assess spontaneous neural

activity, functional connectivity and functional networks; ASL was used to assess cerebral blood flow; DTI was used to assess white matter integrity, anatomical connections and anatomical networks; and DKI was used to assess non-Gaussian diffusion. To reduce the influence of differences in MRI scanners from different manufactures, we encouraged centers to acquire data using the MR 750 scanner of General Electrics for its relatively high image quality and popularity in China at that time.

In blood sample collection and processing session, blood samples were first collected from each participant and isolated into white blood cell and plasma, and then moved to Tianjin Medical University General Hospital (TMUGH) via professional cold-chain transportation to avoid DNA degeneration. The isolated blood samples from each participant were stored separately in five ultralow temperature refrigerators to avoid accidental loss of data. Then, DNA was extracted using a semiautomatic procedure with quality control of DNA concentration and purification. A high-throughput genotyping chip designed for Asian population (Illumina Asian screening array chip) with 700,000 SNPs will be used for genome-wide genotyping with rigorous quality control.

All raw data are stored in both individual center and the leading center (i.e., the TMUGH). All behavioral, environmental and neuroimaging data were preprocessed in TMUGH with the same pipelines.

3. Experimental design

3.1 Sample size

One of the most important objectives of the CHIMGEN study is to identify genetic variants associated with neuroimaging phenotypes using GWAS analyses. The 7000 subjects collected during the first stage of the CHIMGEN study will be divided into a discovery sample (n = 5000) and a validation sample (n = 2000). A recent GWAS analysis from UK biobank for over three-thousand neuroimaging phenotypes has revealed that about 50% neuroimaging phenotypes have SNP heritability more than 0.2. According to GCTA-GREML power calculator, the least number of participants is

4,430 for a statistical power of 0.8. A sample of 5000 subjects is able to identify significant associations for neuroimaging phenotypes with a SNP heritability of 0.2 with a power of 0.89. Therefore, a discovery sample of 5,000 subjects is sufficient to reliably identify genetic variants associated with neuroimaging phenotypes with a moderate heritability.

Reference

Visscher PM, Hemani G, Vinkhuyzen AE, et al. Statistical power to detect genetic (co) variance of complex traits using SNP data in unrelated samples. PLoS Genetics 2014; 10(4): e1004269.

3.2 Experimental procedures

The streamline of the experimental procedures is shown in Figure 1.1, the CVLT module is shown in Figure 1.2, and the RO module is shown in Figure 1.3.

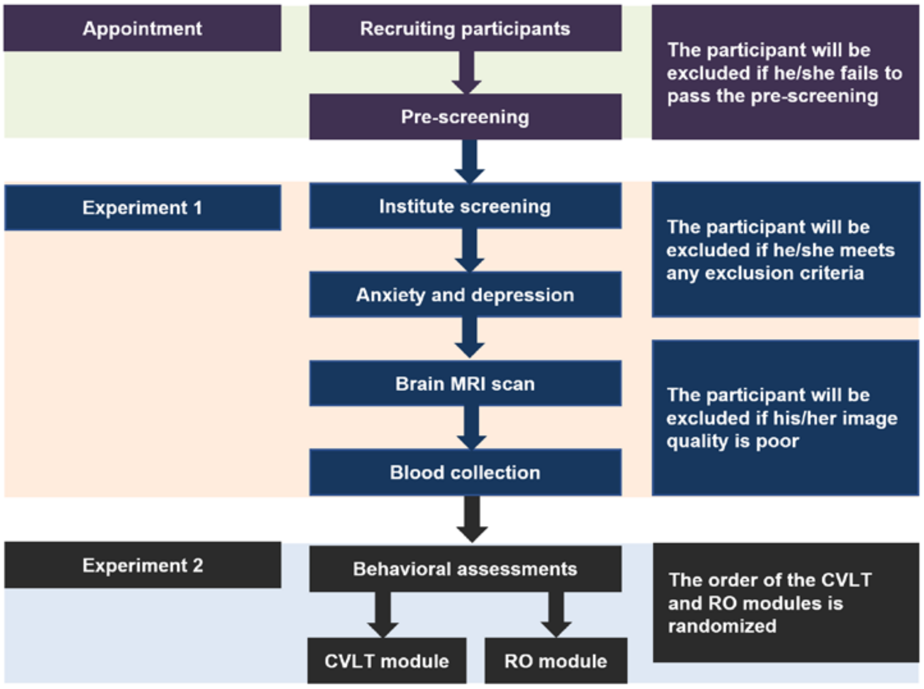

Figure 1.1 Streamline of experimental procedures

3.3 Notes

(1) The interval between Experiment 1 and Experiment 2 is no more than 14 days.

(2) The order of the CVLT module and RO module is randomized.

(3) Because the states of anxiety and depression are time-dependent, the STAI and

BDI-II should be assessed 5-8 minutes before the MRI scan.

(4) Blood sample can be collected after the MRI scan or after the behavioral and environmental assessments.

(5) Females should be scanned and evaluated outside their menstrual periods.

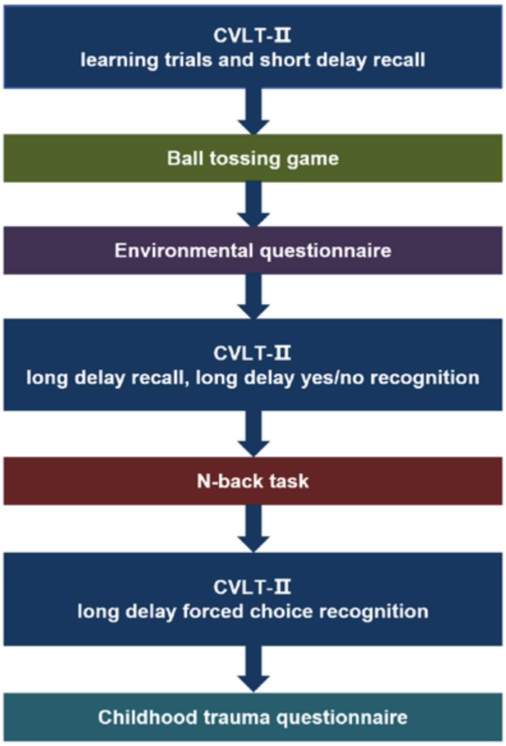

Figure 1.2 CVLT module

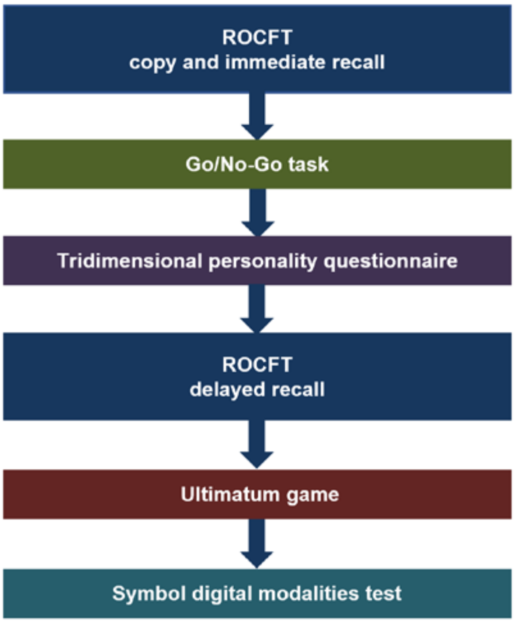

Figure 1.3 RO module

Chapter 2 Screening

1. Pre-screening

1.1 Recruitment

The researchers of the CHIMGEN study post recruitment advertisement (Appendix 2.1), informed consent form (Appendix 2.2) and MRI safety questionnaire (Appendix 2.3) in colleges and communities. When a volunteer contacts the designated researcher, the researcher must complete the following tasks:

(1) The researcher should confirm whether the participant satisfies the inclusion criteria (Chinese Han, age of 18-30 years and right handedness).

(2) The researcher should confirm whether the participant meets any of the exclusion criteria, such as color-blind, over-drinking, neuropsychiatric disorder, severe somatic disorder, a history of drug abuse, or a family member suffering from mental illness, etc.

(3) The researcher should record the information of name, gender, age, contact information, school, grade, and major (for students) or occupation (for non-students) of each participant.

(4) The researcher should explain the details of the experiment, especially informing the participant that:

   (a) The whole experimental process consists of two visits: the participant will receive screening, MRI scanning and blood sample collection in the first visit, and receive behavioral and environmental evaluation in the second visit;

   (b) The interval between the two visits should be no more than 14 days so that the participants can schedule their visits in advance;

   (c) Compensation will be given at the end of the second visit.

(5) The researcher should inform the participant the precautions that need to be taken before and on the day of the two visits.

1.2 Appointment

An appointment should be made for each participant. The time of the appointment

should be arranged so that the participant does not have to wait for a long time after the arrival.

Notes

(1) The experimenter should ensure that the participant has enough time to complete the experiment.

(2) The experimenter should decline the request of watching the experiment from friends or classmates of the participant or any other non-experimental persons.

(3) The experimenter should inform participants that they should have no less than 7 hours sleep at the night before the experiment.

(4) The experimenter should inform participants that they should not do any strenuous exercise or drink strong tea, caffeine-containing and alcoholic beverages on the day of the experiment.

(5) The experimenter should ensure that each female participant is outside her menstruation on the day of the experiment.

(6) The experimenter should ensure that the participant has not attended the same study at other centers.

(7) The experimenter should inform myopic participants to bring glasses.

(8) The experimenter should politely decline any uncooperative volunteers.

1.3 Preparation before experiments

1.3.1 Laboratory preparation

(1) The experimental environment should be clean and quiet.

(2) All experimental facilities should be checked before the experiment.

(3) It is recommended to start the preparation one hour before the experiment.

1.3.2 Experimenter preparation

(1) The experimenter should wear tidily and perform politely.

(2) The experimenter should fully understand the experiment, including the purpose, design, process, instructions and contents of each test.

(3) Two experimenters are required for one test. One experimenter is responsible for introducing the experiment, guiding the participant to complete the experiment,

and monitoring the process of the experiment. The other is responsible for preparing experimental materials, registration, recording, etc.

1.4 Reception

(1) An experimenter will confirm the identity of the participant and guide the participant to complete the experiment.

(2) The experimenter should explain the purpose, procedure, duration and break of the experiment.

(3) Before the experiment, any unrelated persons should leave the laboratory room to create a quiet environment.

(4) If the participant is nervous, the experimenter should explain the safety of the experiment and ask the participant to take a rest to reduce negative emotion.

(5) Both experimenters and participants should turn off or mute their cell phones during the experiment.

2. Institute screening

2.1 Institute screening streamline (Figure 2.1)

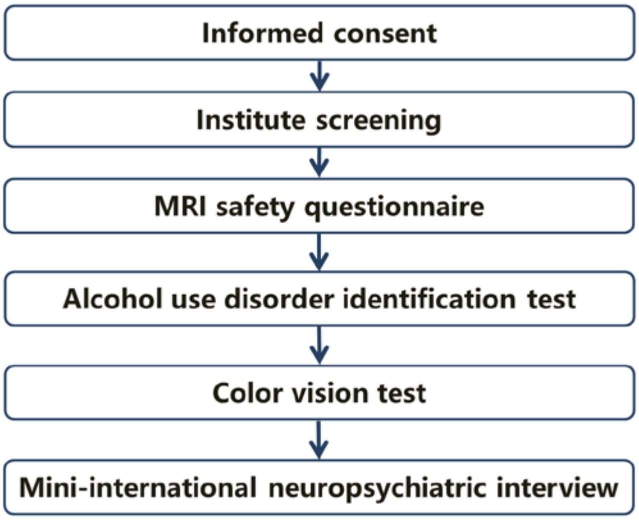

Figure 2.1 Institute screening streamline

Notes

(1) If any requirement is not met, the screening procedure will be terminated.

(2) The participant would be excluded if the mini-international neuropsychiatric interview (MINI) indicates any mental disorder.

2.2 Informed consent form

Before institute screening, each participant should fill in the informed consent form (Appendix 2.2). The experimenter should explain each item of the form to the participant and should ensure that the participant has fully understood the content of the form. The experimenter should answer all questions raised by the participant before the participant signs the form. The main contents of the form include:

(1) Benefits

You and the society will likely benefit from this study. You will receive a free brain magnetic resonance imaging (MRI) examination. If you successfully complete the experiment, you will receive RMB 200 yuan as compensation for your participation.

(2) Risks

The MRI is a safe (without any radiation) examination which has been widely used in clinical practice. However, MRI scanners can produce noise that may make you feel uncomfortable. A small number of persons may feel discomfort due to claustrophobia, in this situation, the participant should inform the experimenter in time to terminate the examination. The collection of blood sample is done by professional nurses and the equipment used throughout the process is safe, non-polluting and disposable.

(3) Privacy protection

All your information will be stored in the individual center and the leading center, and used only for scientific purposes. Only researchers involved in this study, professional academic committees and ethics committees will be allowed to access your records. Any public report about the results of this study will not disclose your personal identity. We will make great effort to protect the privacy of your personal data. You may choose not to participate in this study or to withdraw at any time without discrimination or retaliation. Your participation in this study is fully voluntary. You can keep track of the information related to this study. If you have any questions about this study or about the participants' rights in this study, please do not hesitate to contact us.

2.3 MRI safety questionnaire

Each participant should fill in the MRI safety questionnaire (Appendix 2.3), which is used to determine whether the participant is suitable for a safe MRI scan. The experimenter should ensure that the participant has correctly understood each item, and should carefully answer any question raised by the participant.

2.4 ID number

Each participant will be assigned an ID number (6 digits) to protect his or her privacy. For example, for an ID number 010023, the first two digits (01) indicate the first center and last four digits (0023) indicate the 23rd participant of this center.

2.5 Basic information of participants

Each participant should fill in Table 2.1 which is used to collect the basic information of the participant.

Table 2.1 Basic information

ID number			Recruitment date		
Name		Gender (select with “√”)	Male ( )<br>Female ( )	Birth date	
Ethnicity		Marital status (select with “√”)	Unmarried ( )<br>Divorced ( )	Married ( )<br>Widowed ( )	
Height (cm)		Weight (kg)			
Menstrual cycle (Ladies only)	Menstrual period (days 1st-4th) ( )				
	Follicular maturation stage (5th-14th) ( )				
	Luteal phase (15th-28th) ( )				
Cell phone		E-mail			
Birthplace					
Father’s birthplace		Mother’s birthplace			
College students should fill in the following information					
Degree	Junior college ( ) Full-time undergraduate ( ) Master ( ) PhD ( )				
School		Major			
Grade		Years of education			
Others should fill in the following information					
Career					
Years of education		Highest education			

2.6 Inclusion and exclusion criteria

The inclusion and exclusion criteria are listed in Table 2.2. Each participant should satisfy all the inclusion criteria and not meet any of the exclusion criteria.

Table 2.2 Inclusion and exclusion criteria

If you meet the condition, fill in “√”, if not, fill in “×”	
Age of 18-30 years	
Chinese Han (without any other ethnic ancestors in recent three generations)	
Right handedness (further confirmed by the Chinese handedness questionnaire)	
The total number of cigarettes so far is no more than 20	
Women are neither in pregnancy nor in the menstrual period on the day of the experiment	
Relatives have not participated in this study	
No contraindications for MRI examinations	
No visible brain abnormalities on previous MRI examinations	
No history of alcohol or drug abuse or dependence	
No history of neuropsychiatric diseases (schizophrenia, anxiety, depression, epilepsy, stroke, tumors, and multiple sclerosis, etc.)	
No history of consciousness loss for more than 5 minutes	
No history of brain injury or neurosurgery	
No history of major physical illnesses (heart disease, hypertension, nephritis, diabetes, malignant tumors, hereditary diseases, etc.)	
Currently without any medication (including contraceptives)	
No history of taking drugs (antipsychotics, mood stabilizers, isoniazid, glucocorticoids, stimulants, etc.) that might affect the brain	
No history of using sedative hypnotics (benzodiazepines, barbiturates) within one month	
No family history of neuropsychiatric disorders (three generation relatives)	
No color blindness or any difficulty in color discrimination	
With enough sleep (> 7 hours) at the night before the experiment	
No strenuous exercise or consumption of strong tea, caffeine or alcoholic beverages on the day of the experiment	

2.7 The Chinese handedness questionnaire

(1) Introduction

Based on Annett's hand preference questionnaire and Edinburgh handedness inventory, the Chinese handedness questionnaire was designed to adapt to Chinese culture. The questionnaire consists of 10 questions, including writing, using chopsticks, throwing, tooth brushing, using scissors, striking match, threading needle, holding hammer, holding tennis racket, and washing face using towel.

(2) Instructions for participants

“For each of the following 10 daily activities, please fill in "++" in the corresponding grid if you prefer to use one hand than the other hand, and fill in "+" if there is no a preferred choice.”

(3) Handedness classification

Each participant can be divided into one of the five categories of handedness. Only consistent and inconsistent right handers are included in this study. The criteria for handedness classification are as follows:

(a) If the participant answers left (++) to all questions, he/she is classified as consistent left-handedness.

(b) If the participant answers right (++) to all questions, he/she is classified as consistent right-handedness (Table 2.3).

(c) If the participant answers left (++) to the first six questions and 1-3 of the last four questions, he/she is classified as inconsistent left-handedness.

(d) If the participant answers right (++) to the first six questions and 1-3 of the last four questions, he/she is classified as inconsistent right-handedness (Table 2.3).

(e) Otherwise, the participant is classified ambidexterity.

References

Annett M. A classification of hand preference by association analysis. British journal of psychology 1970; 61(3): 303-321.

Oldfield RC. The assessment and analysis of handedness: the Edinburgh inventory. Neuropsychologia 1971; 9(1): 97-113.

Li X. The distribution of left and right handedness in Chinese people. Acta Psychologica Sinica 1983; 3: 268-276.

Table 2.3 Criteria for consistent and inconsistent right handers

Questions	Right-handers	
	Consistent	Inconsistent
Writing	right (++)	right (++)
Using chopsticks	right(++)	right(++)
Throwing	right (++)	right (++)
Tooth brushing	right (++)	right (++)
Using scissors	right(++)	right(++)
Striking match	right (++)	right (++)
Threading needle	right (++)	right or left (+ or ++)
Holding hammer	right (++)	right or left (+ or ++)
Holding tennis racket	right(++)	right or left (+ or ++)
Washing face using towel	right (++)	right or left (+ or ++)

2.8 Alcohol use disorder identification test

(1) Introduction

The alcohol use disorder identification test (AUDIT) was developed in 1982 and has been used for screening dangerous and harmful drinking behavior. The AUDIT was translated into Chinese in 1999 and its reliability and validity have been validated in Chinese people. That is, a total score of ≥7 can identify participants with dangerous and harmful drinking behavior with excellent sensitivity (99.7%) and specificity (90%). This scale consists of 10 questions (3 about alcohol consumption and frequency, 3 about alcohol dependence, and 4 about problems caused by alcohol use).

(2) Instructions for participants

"This questionnaire is for assessing your alcohol use. Please fill in each question carefully. Please ask if you do not understand any item."

(3) Scoring

The first 8 questions are graded as 5 levels (0, 1, 2, 3, 4), and the last 2 questions are graded as 3 levels (0, 2, 4). Questions 2, 4, 6, and 8 are scored in reverse. In this test, the number of "standard cups" of question 2 and 3 was converted into beer or 56 degrees of liquor according to Chinese culture. The participants with a score of ≥7 should be excluded.

References

Li B, Shen Y, Zhang B, et al. The Test of AUDIT in China. Chinese Mental Health Journal 2003; 1:1-3.

Saunders JB, Aasland OG, Babor TF, et al. Development of the Alcohol Use Disorders Identification Test (AUDIT): WHO Collaborative Project on Early Detection of Persons with Harmful Alcohol Consumption-II. Addiction 1993; 88(6): 791-804.

Zhang C, Yang G, Li Z, et al. Reliability and validity of the Chinese version on Alcohol Use Disorders Identification Test. Chinese Journal of Epidemiology 2017; 8:1064-1067.

2.9 Color vision test

The fifth edition of color blindness test is used to identify participants with color vision deficiencies. The participant should perform the test in a distance of 50-60 cm from the screen and try to find a number, letter, shape or animal on each of the 24 background pictures within 10 s. More than one wrong answer is considered to be color vision impairment.

Reference

Yu Z. Color blindness test chart. 5th Edition Beijing: People's Medical Publishing House, 2005

2.10 Mini-international neuropsychiatric interview

The mini-international neuropsychiatric interview (MINI) is a simple, effective and reliable structured interview tool. The MINI consists of 130 questions and is used for screening participants with mental disorders, including 16 axis I psychiatric disorders and a personality disorder in the fourth edition of Diagnostic and Statistical Manual of Mental Disorders (DSM-IV) and the tenth revision of the International Classification of Diseases for Mental Disorders (ICD-10). Similar to the patient edition of the Structured Clinical Interview for DSM Disorders (SCID-P) and Composite

International Diagnostic Interview Form (CIDI), each diagnosis in MINI is based on a group of questions and most diagnoses can be excluded based on their screening questions. The MINI has been widely used in large-scale clinical epidemiological investigations and multi-center clinical researches because it is time-saving and shows high reliability and validity. The version used in this study is 5.0.0.

References

Sheehan DV, Lecrubier Y, Sheehan KH, et al. The Mini-International Neuropsychiatric Interview (M.I.N.I.): the development and validation of a structured diagnostic psychiatric interview for DSM-IV and ICD-10. Journal of Clinical Psychiatry 1998; 59 (suppl 20): 22-33.

Si T, Shu L, Dang W, et al. Evaluation of the Reliability and Validity of Chinese Version of the M.I.N.I.-International Neuropsychiatric Interview in Patients with Mental Disorders. Chinese Mental Health Journal 2009; 7: 493-497.

Appendix 2.1

Recruitment advertisement

We are conducting a Chinese Imaging Genetics (CHIMGEN) study to identify genetic and environmental factors and their interactions that are associated with neuroimaging and behavioral phenotypes. We sincerely invite you to participate in this study if you are willing to make contributions to medical progress.

1. Experiment content

(1) Behavioral and environmental assessments (paper- and computer-based tests);

(2) Collection of 10ml peripheral blood for genetic examination;

(3) Brain MRI scans.

2. Experiment location

The location should be clearly described. Taking TMUGH as an example, the experiment location is written as: Room 101, Inpatient Building 3, Tianjin Medical University General Hospital, No. 154, Anshan Road, Heping District, Tianjin, China.

3. Number of visits

If you are eligible, you need to pay two visits. The first visit is for screening, MRI scans and blood sample collection. The second visit is for behavioral and environmental assessments. The interval between the two visits is no more than 14 days.

4. Inclusion criteria

To be eligible, you need to meet all the following inclusion criteria:

(1) Age of 18-30 years;

(2) Chinese Han (without any other ethnic ancestors in recent three generations);

(3) Right handedness;

(4) The total number of cigarettes so far is no more than 20;

(5) Women are neither in pregnancy nor in the menstrual period on the day of the experiment;

(6) Relatives have not participated in this study;

(7) No contraindications for MRI;

(8) No visible brain abnormalities on previous MRI examinations;

(9) No history of alcohol or drug abuse or dependence;

(10) No history of neuropsychiatric diseases;

(11) No history of consciousness loss for more than 5 minutes;

(12) No history of brain injury or neurosurgery;

(13) No history of major physical illnesses;

(14) Currently without any medication;

(15) No history of taking drugs that might affect the brain;

(16) No history of using sedative hypnotics within one month;

(17) No family history of neuropsychiatric disorders (three generation relatives);

(18) No difficulty in color discrimination;

(19) With enough sleep (> 7 hours) at the night before the experiment;

(20) No strenuous exercise or consumption of strong tea, caffeine or alcoholic beverages on the day of the experiment.

5. Benefits

(1) Free screening for mental illnesses;

(2) Free brain MRI examination;

(3) RMB 200 yuan will be paid to participants who successfully complete the experiment.

6. Duration of the experiment

It takes about 1.5 hours for the first visit and about 2.5 hours for the second visit.

7. Contact number

The cell phone numbers of the designated researchers.

Appendix 2.2

Informed consent form

Dear participants,

We invite you to participate in the Chinese Imaging Genetics (CHIMGEN) study. Before you decide whether to accept the invitation, please read the following information carefully, which will help you understand the purpose and procedures of this study, as well as the benefits and possible risks of participating in this study. You can discuss with your relatives and friends to help you decide whether to participate in this study. If you have any questions, please do not hesitate to contact the researchers of this study.

1. Introduction

1.1 Aims

This study aims to identify genetic and environmental factors and their interactions that are associated with neuroimaging and behavioral phenotypes.

1.2 Background

This study will be conducted in about 30 centers including hospitals, universities and research institutes in China. It is anticipated that about 10,000 volunteers will participate in this study, which has been approved by the Ethics Committee of Tianjin Medical University General Hospital (TMUGH).

1.3 Process

If you agree to participate in this study, you will need to pay two visits. Screening, MRI examination and blood sample collection will be done on the first visit. Behavioral and environmental assessments will be conducted on the second visit. The interval between the two visits should be no more than 14 days.

1.4 Benefits

You and other people may benefit from the scientific findings derived from this study. The benefits for you include a free head MRI examination, a free screening for mental disorders and a reward of RMB 200 yuan after the successful completion of all tests.

1.5 Risks

There are no known risks for you to perform behavioral and environmental assessments. The magnetic resonance imaging (MRI) examination is a safe clinical imaging method without ionizing radiation. However, the noise produced by MRI scanners may make you feel uncomfortable. A few people may feel extremely uncomfortable due to claustrophobia. In this case, you can inform the experimenter immediately using an alarm ball to stop the MRI examination. Regarding blood sample collection, we guarantee that professional nurses will conduct the blood sample collection and the equipment used in the whole experiment is safe and pollution-free to avoid any risks.

Participants with contraindications for MRI examination are not allowed to participate in this study, including participants who have received pacemaker implantation, metal implants or foreign bodies, and those with severe diseases or claustrophobia.

1.6 Responsibility

Please do not disclose the details of the tests to other people as it may bias their test results if they also choose to participate in this study.

1.7 Privacy protection

All information and results about you will be stored safely in the center from which you are recruited and the leading center (i.e., TMUGH), and will be used only for purposes of scientific research. Only the relevant researchers and the members of the academic committee and the ethics committee will be allowed to access your test records. Any public report on the results of this study will not disclose your personal identity. We will take every measure to protect the privacy of your personal data. You may choose not to participate in this study or withdraw at any time after notifying the researcher without any discrimination or retaliation. Your participation in this study is entirely voluntary. You may request information related to this study at any time. If you have questions about this study or the rights and interests of participants, please do not hesitate to contact the researchers.

2. Agreement statement

I have carefully read the above information of this study. I understand that I have the right for obtaining further information of the study from researchers and for raising questions about this study. All my questions have been answered satisfactorily. I understand the risks and benefits of participating in this study. I understand that the participation is voluntary. I confirm that I have had sufficient time to consider the participation in this study and I understand that:

(1) I can ask researchers for more information at any time.

(2) I can withdraw from this study at any time without discrimination or retaliation.

(3) I agree that the health administration supervision department, ethics committee or professional academic committee can access my data.

After careful consideration, I decide to participate in this study.

Name: Date: Telephone number:

Appendix 2.3

MRI safety questionnaire

This form must be filled before MRI examination. If you have any questions, please do not hesitate to ask the researchers of this study. Please fill in "√" if you have it or "×" if you do not have it.

Name		Gender	Male ( ) female ( )	Age (years)	
**If you meet any of the following items, please do not enter the scanning room!**					
1	Pacemakers				
2	Cardiac defibrillators				
3	Metal clip after intracerebral aneurysm surgery				
4	A drug infusion device implanted into the body				
5	Bone growth stimulator				
6	Nerve stimulator				
7	Any other type of stimulator				
8	Hearing-aid, cochlear implants				
9	Metal foreign object in the eye				
10	Any other electronic device implanted in the body				
**If you meet any of the following items, please report!**					
1	Any type of surgical clip or material in the body				
2	Artificial heart valve				
3	Inferior vena cava filter				
4	Endovascular embolization ring				
5	Middle ear implant				
6	Penile prosthesis				
7	Ocular prosthesis				
8	Bullet or shrapnel				
9	Metal sutures				

10	Eyeliner or tattoos	
11	Renal shunt	
12	After cardiac shunt surgery	
13	An artificial limb or joint	
14	Orthopedic postoperative fixation device (needle, nail, screw, etc.)	
15	Denture, denture holder and dental implants	
16	Intrauterine device (IUD)	
17	Hair gel, cosmetics	
If yes, please provide detailed information:		
18	Do you have a history of surgery	
If yes, please provide the type of surgery:		
19	Pregnancy	

To the best of my knowledge, I confirm that the above information is correct.

Participant signature___________________

Date___________________

Experimenter's signature____________________

Date____________________

Chapter 3 Behavioral and environmental assessments

1. Introduction

To avoid fatigue and to ensure the quality of assessments, the behavioral and environmental assessments are divided into two separate modules. The first is the California verbal learning test (CVLT-II) module (Table 3.1) and the second is the Rey-Osterrieth (RO) complex figure test module (Table 3.2).

Table 3.1 The CVLT-II module (about 70 minutes)

**1. Episodic memory 1 (25 min)**<br>CVLT-II learning trials and short delay recall (25 min)
**2. Recall interval (20 min)**<br>(1) Social cognition: ball tossing game (16 min)<br>(2) Environmental assessments: environmental questionnaire (4 min)
**3. Episodic memory 1 (10 min)**<br>CVLT-II long delay recall, long delay yes/no recognition (10 min)
**4. Recall interval (10 min)**<br>Working memory: N-back task (10 min)
**5. Episodic memory 1 (5 min)**<br>CVLT-II long delay forced choice recognition (5 min)
**6. Environmental assessments (3min)**<br>Childhood trauma questionnaire (3min)

Notes

(1) Gray box represents the fixed recall interval. If the required assessments within the interval are completed in advance, the participant should perform a mathematical task “100 – 7 = ?” to prevent the participant from recalling.

(2) The state-trait anxiety inventory (STAI) and Beck depression inventory-II (BDI-II) should be assessed just before the MRI scan as the anxiety and depression states are time-dependent.

(3) If there is no enough time to complete the questionnaire during the interval, the questionnaire can be completed at the end of this module.

Table 3.2 The RO module (about 70 minutes)

**1. Visual spatial capability (10 min)**<br>ROCFT copy and immediate recall (10 min)
**2. Recall interval (25 min)**<br>(1) Executive function: Go/No-Go task (12 min)<br>(2) Personality: tridimensional personality questionnaire (13 min)
**3. Episodic memory 2 (10 min)**<br>ROCFT delayed recall (10 min)
**4. Fair decision making (15min)**<br>Ultimatum game (15min)
**5. Information processing speed (3min)**<br>Symbol digital modalities test (SDMT) (3min)

2. Tridimensional personality questionnaire

(1) Introduction

The biosocial theory divides personality into three independent dimensions linking to different neurotransmitters: novelty-seeking (NS) to dopamine (DA); harm-avoidance (HA) to serotonin (5-HT); and reward dependence (RD) to norepinephrine (NA). Based on this theory, the tridimensional personality questionnaire (TPQ) was designed to include 3 dimensions and 12 factors, totally 100 questions. It takes about 10 minutes to answer all questions. In this study, we use the fourth edition of the questionnaire (Chinese version). The validity and reliability have been confirmed.

(2) Procedures

Items in the TPQ describe personal attitudes, opinions, interests and feelings and there are no correct or incorrect answers. During the test, participants should read each item carefully, and choose the one that best suits their actual situation or answer with "yes" or "no". Participants cannot spend much time thinking how to answer the question and they can answer questions even without thorough thinking.

(3) Notes

During the test, if the participant has doubts about an item, the experimenter should explain to the participant in detail, but do not directly tell the participant the choice. We delete items 61 and 71 because they have been reported to be lacking of

specificity. Besides, the 49th item was moved from the factor RD2 to the factor HA4.

References

Cloninger CR. Tridimensional Personality Questionnaire (TPQ), version 4. Washington University Medical School, St Louis, MO, 1987.

Cloninger CR, Przybeck TR, Svrakic DM, et al. The Tridimensional Personality Questionnaire: U.S. normative data. Psychological Reports 1991; 69(3): 1047-1057.

Duan M, Yang Y, Li B. Validity and Reliability of Tri-dimensional Personality Questionnaire. Chinese Mental Health Journal 2006; 9: 610-612.

3. Mood assessments

3.1 State-trait anxiety inventory

(1) Introduction

The state-trait anxiety inventory (STAI) is a common tool to differentiate between short-term anxiety state and anxious personality trait. The STAI has been widely used to assess anxiety state and trait in many kinds of populations including college students. The first edition was published in 1970s and was translated into Chinese in 1988. The STAI is a self-reporting scale consisting of 40 descriptive questions that can be divided into two categories:

(a) State anxiety inventory (S-AI)

The S-AI includes Questions 1-20, which describe unpleasant emotional experiences, usually transient, such as stress, fear, anxiety and nervousness, accompanied by hyperactivity of the autonomic nervous system.

(b) Trait anxiety inventory (T-AI)

The T-AI includes Questions 21-40, which describe relatively stable, anxious tendency that is a personality trait with individual differences.

(2) Procedures

(a) S-AI (Questions 1-20)

Instructions given to participants are: “Listed below are some of the statements people often use to describe themselves. Please read each statement and then pick a number on the right to indicate your most appropriate feeling at present. There is no right or wrong answer. Do not spend too much time on any question, but the answer should be the most appropriate feeling you are having at this moment.”

(b) T-AI (Questions 21-40)

Before testing, the researcher should explain the differences between the first 20

questions and the second 20 questions, emphasizing that the second 20 questions are about your feelings in general rather than at this moment. Instructions given to participants are: “Listed below are some statements that people often use to describe themselves. Please read each statement and then pick a number on the right to indicate how you feel in general. There is no right or wrong answer. Do not spend too much time on any question, but the answer should be what you usually feel.”

(c) Notes

The experimenter should ensure that the participant has understood the difference between S-AI and T-AI questions and can accurately answer these questions according to the instruction.

(3) Scoring

This questionnaire is a 4-point Likert scale (1: not at all; 2: some; 3: moderate; and 4: obvious). Participant should pick the most appropriate level according to their own experience. The individual’s score is calculated as the cumulative scores of STAI, with a minimum of 20 points and a maximum of 80 points. It should be noted that all positive emotion items (Questions 1, 2, 5, 8, 10, 11, 15, 16, 19, 20, 21, 23, 24, 26, 27, 30, 33, 34, 36, and 39) are scored in reverse order. The higher STAI score indicates the higher level of anxiety.

References

Shen Y. Psychiatry, Second Edition, Beijing: People's Medical Publishing House, 1988: 243-263.

Spielberger CD, Gorsuch RL, Lushene RE, et al. Manual for the State-Trait Anxiety Inventory (Form Y1 - Y2). Palo Alto Ca Consulting Psychologists Press, 1983.

Zheng X, Shu L, Zhang A, et al. Test report of state-trait anxiety inventory in Changchun. Chinese Mental Health Journal 1993; 7(2): 60-62.

3.2 Beck depression inventory-II

(1) Introduction

The Beck depression inventory (BDI) is the most commonly used self-scoring scale for depressive symptoms. The first edition (BDI-I) was introduced in 1964. The BDI-II was introduced in 1996 to adapt to the diagnostic criteria of depressive disorders in DSM-IV. The BDI-II has been translated into Chinese and showed good reliability and validity in Chinese population. The Chinese version of the BDI-II

consists of 21 items and each item has 4 statements indicating different levels of a symptom.

(2) Procedures

(a) Instructions for participants: “There are 21 sets of statements in this questionnaire. Please read each statement carefully and select the one most accurately describing your feelings in the last two weeks (including today). For a given set of statements, only one statement can be selected. If more than one statement is right for you, choose the most severe one.”

(b) The test requires the participant to select the number in front of the sentence that best suits his or her situation based on the feelings of the last two weeks.

(c) During the test, the participant may feel that none of the four options describe his/her feelings of the past two weeks. In this case, the participant should choose the one closer than others.

(3) Scoring

The BDI-II includes 21 items, each item with a score of 0-3. Therefore, the total score of 21 items ranges from 0 to 63. A total score of 0-13 indicates no depression; 14-19 indicates mild depression; 20-28 indicates moderate depression; and 29-63 indicates severe depression.

References

Beck AT, Steer RA, Brown GK. Manual for the beck depression inventory-II. 1996.

Wang Z, Yuan C, Huang J, et al. Reliability and validity of the Chinese version of Beck Depression Inventory-II among depression patients. Chinese Mental Health Journal 2011; 6: 476-480.

Yang W, Wu D, Peng F. Application of Chinese version of Beck Depression Inventory-II to Chinese first-year college students. Chinese Journal of Clinical Psychology 2012; 20(6): 762-764.

4. Paper-based cognitive assessments

4.1 California verbal learning test-II

(1) Introduction

The second edition of the California verbal learning test (CVLT-II) was introduced in 2000. In contrast to other memory tests, the CVLT adopts a process-oriented approach to provide different quantitative and qualitative profiles of learning and memory impairments. The difficulty of the word list in CVLT-II was reduced to be better

applied in a larger normal population. We used the Chinese version of CVLT-II. The CVLT-II was standardized on a normative sample ranging from 16 to 89 years that was consistent with the demographic characteristics of the CHIMGEN study.

(2) Procedures

For the word list A, the experimenter reads the words in one word per second in Mandarin and asks the participant to learn five continuous times and to recall after each learning time. The word list A consists of 16 words that can be divided equally into 4 semantic categories (furniture, vegetable, vehicle and animal). The words are randomly presented and are not arranged according to semantic categories. The word list B is used as interference and consists of 16 words of two categories (vegetable and animal) from the word list A and two different categories of musical instrument and house part name. Participants are required to learn word list B once before short delay free recall of the word list A. After 20 minutes of non-language testing, a long delay free recall, cued recall, and a yes/no recognition test for word list A are performed. The forced choice recognition test should be performed 10 minutes later from the long delay yes/no recognition test.

(3) Notes

(a) If the participant asks the experimenter whether he/she recalls it correctly during the recall interval, the experimenter should answer: “Please do not worry about whether it is correct. Please answer it truthfully”.

(b) The recalled words by participants must be recorded by the experimenter. If a word is recalled twice, it should also be recorded in the list in order.

(c) The experimenter should control the recall and interval periods. If the participant cannot recall it for a long time, ask the participant whether to proceed to the next step. Do not extend the time indefinitely.

References

Delis DC, Freeland J, Kramer JH, et al. Integrating clinical assessment with cognitive neuroscience: construct validation of the California Verbal Learning Test. Journal of consulting and clinical psychology 1988; 56(1): 123-130.

Delis DC, Kramer JH, Kaplan E, et al. CVLT, California Verbal Learning Test: Adult Version: Manual. Psychological Corporation, 1987.

Donders J. A Confirmatory Factor Analysis of the California Verbal Learning Test–Second Edition (CVLT-II) in the Standardization Sample. Assessment 2008;

15(2): 123-131.

Zhang N, Li YJ, Fu Y, et al. Cognitive impairment in Chinese neuromyelitis optica. Multiple Sclerosis Journal 2015; 1352458515576982.

4.2 Rey-Osterrieth complex figure test (ROCFT)

(1) Introduction

The Rey's complex figure test was designed in 1941 to assess visuospatial capability and visual memory in patients with brain injury. In 1944, Osterrieth standardized the testing procedures with his scoring system (ROCFT) and provided the norm for children and adults. The ROCFT has become a widely used neuropsychological test to assess visuospatial capability and non-verbal memory.

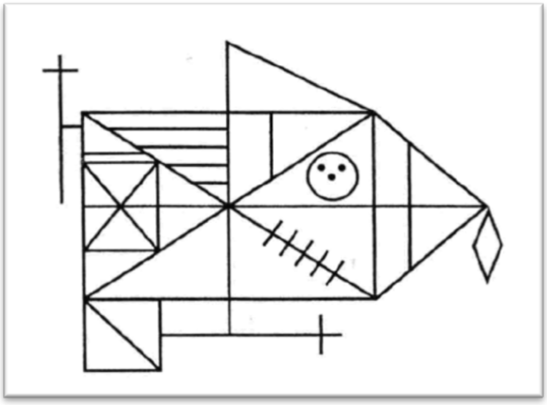

Figure 3.1 The ROCFT figure

(2) Procedures

The assessment materials include red and black pens, timer, ROCFT figure (Figure 3.1) and the scoring manual. Before the test, the experimenter should inform the participant that he/she will redraw the figure in a later recall section.

Step 1: Copy

(a) There is no draft. The participant can mark the wrong part with a red pen.

(b) The experimenter should put the card with the ROCFT figure and the blank test paper horizontally on the table in front of the participant.

(c) The participant is instructed to copy the figure as carefully and accurately as possible without a time limit.

(d) The timing begins after the instruction. If the participant tries to rotate the card or the blank test paper before drawing, the experimenter should return it to the horizontal position. However, if the participant rotates the card after having started copying, there is no need to intervene and just record it.

(e) When the participant completes the task, the experimenter should record the time spent in copying and remove the ROCFT figure and the picture drawn by the participant.

(f) The experimenter cannot tell the participant that he/she will redraw the figure in later recall section at this time.

Step 2: Immediate recall

(a) There is no draft. The participant can mark the wrong part with a red pen.

(b) The experimenter should put a blank test paper horizontally in front of the participant immediately after the end of copy step.

(c) After placing the test paper on the table, the participant will be given the following instruction: "Next, please redraw the figure from memory. You have enough time to draw as carefully and accurately as possible." Then start the timer.

(d) When the participant completes this task, the experimenter should record the time spent and remove the figure drawn by the participant.

Step 3: Delayed recall

(a) There is no draft. The participant can mark the wrong part with a red pen.

(b) After waiting for 25 minutes, the experimenter puts a blank test paper horizontally in front of the participant.

(c) The participant will be given the instruction: "I asked you to copy a figure a little while ago. Now please redraw the figure from your memory again. You have enough time to draw as carefully and accurately as possible." Then start the timer.

(d) When the participant completes this task, the experimenter should record the time spent and remove the figure drawn by the participant.

(3) Assessment time

(a) The total test time is 40-60 minutes.

(b) The time for copy is usually within 5-10 minutes.

(c) The time for immediate recall is usually within 5-10 minutes.

(d) The time for delayed recall is usually within 5-10 minutes.

(e) The interval between immediate and delayed recall is 25 minutes.

(4) Scoring

The ROCFT scoring is susceptible to inter-rater variability, so all ROCFT scoring is completed by a trained and fixed group of raters to achieve highly consistent results. The ROCFT score is calculated based on scoring guideline for each element of the

ROCFT.

References

Guo Q, Lui C. Application of Rey-Osterrieth complex figure test in Chinese normal old people. Chinese Journal of Clinical Psychology 2000; 8(4): 205-207.

Loring DW, Martin RC, Meador KJ, et al. Psychometric construction of the Rey-Osterrieth complex figure: Methodological considerations and interrater reliability. Archives of Clinical Neuropsychology 1990; 5(1):1-14.

4.3 Symbol digit modalities test

(1) Introduction

The symbol digit modalities test (SDMT) is used to assess attention segmentation, visual scanning, tracking and motion speed. The SDMT used in this study was selected from the MCCB Chinese Norm Manual. The keys in the test contain 9 different abstract symbols, each corresponds to a number. The participant is asked to see the keys, to write the number corresponding to each symbol as fast as possible within 90 s.

(2) Procedures

The test is divided into the practice and the formal test. The practice is designed to help the participant understand the experiment and remember symbols. In the practice, the experimenter can observe on the side. If the participant is obviously confused and wrong, the experimenter can explain and correct the error immediately.

(a) Instructions for practice

"Please look at these squares *(the experimenter should point to the diagram on the table with his or her finger)*, you can see a symbol in each square of the upper row. In the square below the symbol *(point with finger)*, there is a corresponding number. According to this paired relationship between the symbol and the number, please fill in the number in each blank square that is corresponding to the above symbol (Figure 3.2). For example, the second symbol corresponds to the number 5 and then you should fill number 5 in the blank square below this symbol. If you make a mistake, please do not amend it and just fill in the correct answer besides the wrong answer. Now, please fill in the remaining blank squares in order until the thick black line for practice."

(b) Instructions for the formal test

"When I say start, please fill in the numbers as soon as possible in the manner that

you have practiced. The sooner is the better. The formal test will begin after the black thick line and the time limit is 90 s."

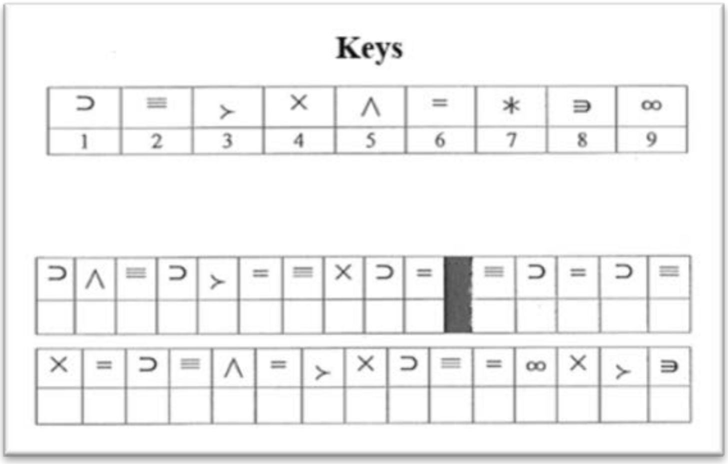

Figure 3.2 SDMT

(3) Scoring

The sum of correctly filled numbers in the formal test within 90 s is the final score and the highest score is 110 points. The number of incorrectly filled numbers should be recorded but should not be included in the calculation of the final score.

Reference

Guo Q. Neuropsychological assessment, Shanghai Science and Technology Press, 2013.

Yu X. MCCB Chinese Norm Manual, Peking University Medical Press, 2014.

5. Computer-based assessments

The computer-based assessments are designed and conducted using the Eprime 2.0. Each test consists of two parts: the practice and the formal test. Each center should prepare 2 fixed computers: an in-use one and a spare one. The spare computer will be used when the in-use one does not work. The experimenter should carefully examine the working state of the used computer and software before the experiment. The participant should sit with a comfortable position at 50-60 cm in front of the screen, and the preset resolution of the computer is 1024×768. The raw data of each test of each participant should be stored in different folders.

5.1 Go/No-Go task

(1) Introduction

The Go/No-Go task is used to assess the ability of behavioral inhibition. In the experiment, the letter X or Y will appear on the screen (Figure 3.3). In "Go" condition, the current letter is different from the previous letter and the participant needs to

respond quickly by pressing the button. In “No-Go” condition, the current letter is the same as the previous letter (10% of all trials) and the participant cannot press the button. If one pressed the button, it would be counted as an error.

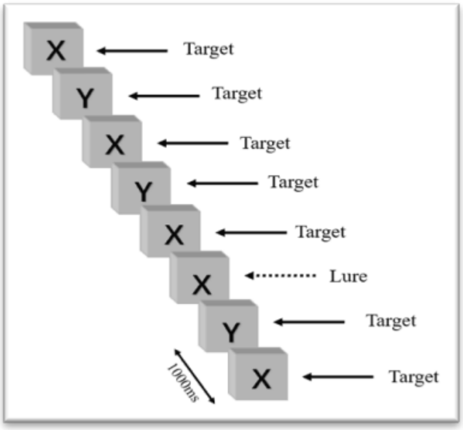

Figure 3.3 Diagram of Go/No-Go task

(2) Procedures

The task consists of the practice and the formal test. There are 20 trials (15 “Go” trials and 5 “No-Go” trials) in the practice. If the participant responds correctly in 3 “No-Go” trials, he or she can shift to the formal test. Otherwise, the participant needs to restart the practice. The formal test is divided into two groups with 210 trials in each group and 30 s break between the two groups. It takes about 12 min for the practice and the formal test. The letter is presented at a frequency of 1 Hz. In “Go” conditions, participants should respond within 900 ms.

(3) Outputs

The results of this task are rendered in the following format (Table 3.3).

Table 3.3 Output of Go/No-Go task

	CRRT	CR	FA	FART	NR
Go					
No-Go					

Abbreviations: CR, number of correct reactions; CRRT, the mean reaction time of correct reactions; FA, false alarm (number of wrong reactions); FART, the mean reaction time of wrong reactions; and NR, number of no reactions.

Reference

Kaufman JN, Ross TJ, Stein EA, et al. Cingulate hypoactivity in cocaine users during a GO-NOGO task as revealed by event-related functional magnetic resonance imaging. Journal

of Neuroscience 2003; 23(21): 7839-7843.

5.2 N-back task

(1) Introduction

The letter N-back is used to assess working memory, the ability of an individual to temporarily maintain and manipulate information during cognitive tasks. In the task, the participant is presented with a sequence of stimuli and the participant should figure out whether the current stimulus matches the one from n steps earlier in the sequence. The load factor n can be adjusted to make the task more or less difficult. For example, the 3-back task needs the participant to compare consistency between the current stimulus and the stimulus from 3 steps earlier in the sequence. The correct rate of the 1-back task is also used to assess the attitude of participants and only the ones with the correct rate above 75% can perform the formal test. The participant should be informed that the first letter of 1-back and the first three letters of 3-back need not to respond and the first letter is the target stimulus. Due to the lack of inter-individual discrimination of 2-back task in healthy young adults in our preliminary analysis, the formal test is performed in the order of 1-back and 3-back. There are only 1 block for 1-back and 1 block for 3-back, and 60 trials for each block with a total of 120 trials. The presentation time of each stimulus is 200 ms and the inter-stimulus interval is 1800 ms.

(2) Procedures

This experiment includes 1-back and 3-back tasks. The experimenter should introduce the task for the participant. It will take about 10 min for the practice and the formal test. The specific procedures are as follows:

(a) After clicking ‘Run’, the following information will be present.

Hello, welcome to the test.<br>The test has two parts: 1-back and 3-back.<br>Please press space bar to read instructions, and then to practice.

(b) After pressing the space bar, the following information will be present.

1-back<br>Please press space bar to continue.

(c) After pressing the space bar, the following information will be present.

You will see a capital letter on the screen, and only one letter at a time.<br>When the current letter is the same as the previous one, press "←" key.<br>When the current letter is different from the previous one, press "→" key.<br>Note: Do not press any button when the first letter appears.<br>Please press the button as accurately and quickly as possible.<br>Please press space bar to practice.

(d) After pressing the space bar to practice, the following information will be present.

This is the practice for you to understand the test procedure.<br>Please put you right index finger on the "←" key and right middle finger on the "→" key.<br>Press space bar to continue.

(e) Formal test: After practicing 1-back and 3-back, the formal test is performed.

(3) Outputs

The results of the task will be rendered in the following format (Table 3.4).

Table 3.4 Output of N-back task

	NTCRRT	THRT	CR	H	M	FA	NR	TRT	TCR
1-back									
3-back									

Abbreviations: CR, the number of correct rejections; FA, false alarms (one should have pressed the "→" key but pressed the "←" key); H, the number of correct hits; M, the number of missing reactions (one should have pressed the "←" key but pressed the "→" key); NR, the number of no reactions; NTCRRT, the mean reaction time of the non-target correct rejections; TCR, the total correct rate; THRT, the mean reaction time of the target hits; and TRT, the mean reaction time of the total correct reactions.

References

Braver TS, Cohen JD, Nystrom LE, et al. A parametric study of prefrontal cortex involvement in human working memory. Neuroimage 1997; 5(1):49-62.

Owen AM, Mcmillan KM, Laird AR, et al. N-back working memory paradigm: a meta-analysis of normative functional neuroimaging studies. Human Brain Mapping 2005; 25(1): 46-59.

Zhang X, Yu JT, Li J, et al. Bridging integrator 1 (BIN1) genotype effects on working memory, hippocampal volume, and functional connectivity in young healthy individuals. Neuropsychopharmacology 2015; 40(7): 1794-1803.

5.3 Ultimatum Game

(1) Introduction

The ultimatum game (UG) is used to investigate fairness. In the task, two persons (A and B) allocate a sum of money (10 yuan). Person A is the proposer to raise an allocation plan (such as 6 yuan for himself and 4 yuan for Person B). Person B is the judge, who can accept (both persons divide the money according to this plan) or reject the plan (both cannot get the money; or only Person A gets the money); or Person C is the judge, but this person does not participate in the allocation. Both Person A and Person B are virtual characters. To make the participant convince that someone really allocates the money, the participant is asked to propose several allocation plans before the test and is informed that the plan will be used for others, and that the proposal he/she received is also proposed by others. Moreover, we will take a random sample from the participant's decision as his final reward.

(2) Procedures

I. Experimental schemes

The ultimatum game in this study includes three situations:

Situation 1: Participate in the allocation with punishment

In this situation, the participant acts as Person B to choose to accept or reject the plan proposed by Person A. If Person B accepts the plan, Person A and Person B will divide the money according to this plan. If Person B rejects the plan, neither Person A nor Person B gets the money.

Situation 2: Participate in the allocation without punishment

In this situation, the participant acts as Person B to choose to accept or reject the plan proposed by Person A. If Person B accepts the plan, Person A and Person B will divide the money according to this plan. If Person B rejects the plan, Person B cannot get any money, but Person A can get the money according to the plan.

Situation 3: Judge but not participate in the allocation

In this situation, the participant acts as Person C to choose to accept or reject the plan proposed by Person A. If Person C accepts the plan, Person A and Person B will divide the money according to this plan. If Person C rejects the plan, neither Person A nor Person B gets the money.

The game includes the practice and the formal test and the total time is about 13 min.

II. Specific procedures

(a) Instructions for participants

"There are 36 rounds in each situation, and you have a maximum of 3 seconds to think. When all the tests are completed, we will randomly pick a round of tests in situation 1 and situation 2 and pay you according to your choice. For example, you will receive 3 yuan after the experiment if you choose to accept the allocation plan of 7 yuan for the proposer and 3 yuan for you."

The participant can read instructions by clicking 'Run' button and pressing the space bar. The following contents will be present one by one:

Hello, welcome to the ultimatum game!<br>The test includes three situations: (1) participate in the allocation with punishment; (2) participate in the allocation without punishment; (3) judge but not participate in the allocation.<br>Press space bar to read the instructions and practice

Situation 1: Participate in the allocation with punishment<br>Please press space bar to continue

Now you and another person allocate a sum of money, and the allocation plan is proposed by that person. You can accept the plan and allocate money according to the plan, or reject it, then neither of you gets the money.<br>You have a maximum of 3 seconds to consider for each decision.<br>Please press space bar to continue

Situation 2: Participate in the allocation without punishment<br>Please press space bar to continue

Now you and another person allocate a sum of money, and the allocation plan is proposed by that person. You can accept and allocate money according to the plan, or reject it, then that person can get the money according to the plan, and you cannot get the money.<br>You have a maximum of 3 seconds to consider your decision.<br>Please press space bar to continue

Situation 3: Judge but not participate in the allocation<br>Please press space bar to continue

Now two other participants allocate a sum of money, and the allocation plan is proposed by one of them. You can accept the plan and they allocate money according to the plan, or you can reject it, then neither of them gets the money.<br>You have a maximum of 3 seconds to consider your decision.<br>Please press space bar to continue

Now, this is the practice section for you to understand the test procedure.<br>Please put right index finger on the “1” bar and right middle finger on the “2” bar.<br>Press space bar to continue.

(b) Practice

After the participant read instructions, he/she can start to practice by pressing the space bar. The following contents will be present one by one:

Situation 1: Participate in the allocation with punishment<br>Please press space bar to continue

Proposer You<br>6 yuan 4 yuan<br>Press “1” for accept<br>Press “2” for reject

Situation 2: Participate in the allocation without punishment<br>Please press space bar to continue

Proposer You<br>7 yuan 3 yuan<br>Press “1” for accept<br>Press “2” for reject

Situation 3: Judge but not participate in the allocation<br>Please press space bar to continue

Proposer Responder<br>6 yuan 4 yuan<br>Press “1” for accept<br>Press “2” for reject

(c) Allocation plans

After the practice, the participant needs to propose 5 allocation plans in the form of allocation ratios (1:9, 2:8, 3:7, 4:6, 5:5, 6:4, 7:3, 8:2, 9:1) and total allocation amount (5/10/15/20 yuan) (Table 3.5). For example, the ratio is 5:5 and the total amount is 10

yuan. The experimenter need to inform the participant that the 5 allocation plans will be used to other participants, and the allocation plans in the formal test are also proposed by other participants.

Table 3.5. Proposal for allocation plans

	Plan 1	Plan 2	Plan 3	Plan 4	Plan 5
Amount					
Ratio					

(d) The formal test

In the formal test, the three situations are carried out separately. Each situation consists of one block, and each block consists of 36 stimuli.

(e) Payment to the participant

At the end of the test, the program will automatically take a random round from situation 1 and situation 2 and present the participant's specific choice and additional rewards for this round of assignment. The experimenter will pay the corresponding rewards to the participant based on this result.

According to your choice, you get 4 yuan

(f) Records

The acceptance ratio and average reaction time under the different allocation ratio will be recorded.

(4) Outputs

The results of the task will be rendered in the following format (Table 3.6).

Table 3.6 Output of the ultimatum game

	**Allocation ratio**	**Average reaction time**	**Acceptance rate**
**Participate in the allocation**	1:9		
	2:8		
	3:7		
	4:6		
	5:5		
	6:4		
	7:3		
	8:2		
	9:1		

**Not participate in the allocation**	1:9		
	2:8		
	3:7		
	4:6		
	5:5		
	6:4		
	7:3		
	8:2		
	9:1		

References

Corradi-Dell'Acqua C, Civai C, Rumiati R I, et al. Disentangling self-and fairness-related neural mechanisms involved in the ultimatum game: an fMRI study. Social cognitive and affective neuroscience 2013; 8(4): 424-431.

Güth W, Schmittberger R, Schwarze B. An experimental analysis of ultimatum bargaining. Journal of economic behavior & organization 1982; 3(4): 367-388.

5.4 Ball-tossing game

(1) Introduction

The ball-tossing game is used to assess the ability of perspective taking linking to distinguish self-other representations. The test is a 2 × 2 factorial design, with agency (active vs. passive) and perspective taking (first-person vs. third-person perspective) as the two factors.

(2) Procedures

(a) Experimental design

The experimental design is shown in Figure 3.4. In the game, three virtual characters represented by different colors (red, green and blue) will appear in the triangle, and the participant needs to act as the **blue** one to complete the active or passive tasks. The definition of active or passive tasks relies on whether the ball is in the hands of the "agent character" (hereinafter referred to as the participant). If the participant has the ball, he needs to pass the ball to the **red** character. If the red character is on his left, he needs to press the "F" button on the keyboard with his left index finger, otherwise he needs to press the "J" button with his right index finger (if the red character is on the right). After the participant reacts, the ball will be thrown to the corresponding character. If the participant doesn't have a ball, he must indicate the ball's position (on his left or right) and press the corresponding button (F or J). If the participant judges

correctly, the ball holder will pass the ball to him, otherwise the ball holder will pass it to the third character. In the first-person perspective, the participant sees from the perspective of the blue character, and the body of the blue character is not displayed on the screen but only one hand stretched in front. In the third-person perspective, the perspective of the participant is different from that of the blue character. At this time, the participant needs to find the blue character on the screen and make his own judgments based on the perspective of the blue character.

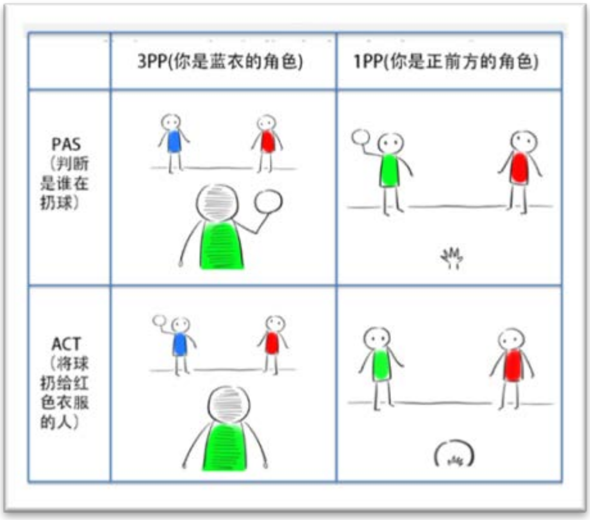

Figure 3.4 Diagram of ball-tossing game

(b) Practice

The test consists of the practice (about 6 min) and the formal test (about 6 min). In the practice, the participant should practice the first- and third-person perspective tasks, separately. For each task, the correct rate should reach more than 75%.

(c) The formal test

The formal test consists of 4 blocks and each block has 18 trials. The first-person (1PP) and third-person (3PP) perspectives are in different blocks in the order of 1PP-3PP-3PP-1PP. The variables such as the location of the participant, the location of the ball-holder, and the relative position of the red character are pseudo-random. In each block, the active test is 6 times and the passive test is 12 times. For a single test, the cross gaze point appears for 0.8s; the characters will appear for 3s, the subject needs to react within 3s, and the response after 3s is not included in the statistics.

(d) Feedback

According to the reaction of the participant, an animation of the ball movement is presented as a feedback (Figure 3.5).

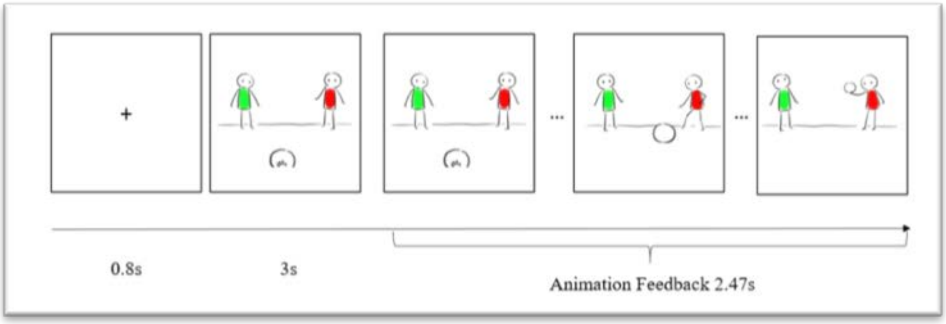

Figure 3.5 Animation diagram of the ball movement

(3) Outputs

The results of the task will be rendered in the following format (Table 3.7).

Table 3.7 Output of ball-tossing game

	RT	ACC	ACT_RT	ACT_ACC	PAS_RT	PAS_ACC
1PP						
3PP						

Abbreviations: ACC, the total correct rate; ACT_ACC, the correct rate in the active state; ACT_RT, the mean reaction time in the active state; PAS_ACC, the correct rate in the passive state; PAS_RT, the mean reaction time in the passive state; PP, personal perspective; and RT, the total mean reaction time.

Reference

David N, Bewernick BH, Cohen MX, et al. Neural representations of self-versus other: visual-spatial perspective taking and agency in a virtual ball-tossing game. Journal of Cognitive Neuroscience 2006; 18(6): 898-910.

6. Paper-based environmental assessments

6.1 Comprehensive environmental questionnaire

(1) Introduction

It is well-known that environmental factors influence on structural and functional properties of the human brain and cognitive functions. Therefore, a comprehensive environmental questionnaire is compiled in this study (Table 3.8).

(2) Notes

(a) This questionnaire contains the information of personal privacy. During the test, the experimenter should not peek at the answers, but should aid if needed.

(b) Several questions are designed to validate the accuracy and correctness of answers for screening questions from the participant. If the answers are inconsistent, the correct information should be confirmed or the item is marked as unreliable.

Table 3.8 Comprehensive environmental questionnaire

Please fill the "blank" or select the appropriate answer with "√"<br>(You can choose more options as long as they are not contradictory)
**Part 1: General information**
1. How old were your parents when you were born?
Father: ______ years old ;   Mother: ______years old
2. How old are you?
____years old
3. What brand of cigarettes have you ever smoked (>20)?
A. Having _______ cigarettes   B. No smoking or no more than 20 cigarettes so far
Are you an only child?
A. Yes   B. No
What season were you born in?
A. Spring (from March to May)   B. Summer (from June to August)<br>C. Autumn (from September to November)   D. Winter (from December to February)
**Part 2: Urbanization and migration**
1. Where was your household registration before the age of 18?<br>(Specific to the village for rural area and to the district and county for urban area; the change in household registration within the same district should not be counted)
1.____________________<br>2.____________________<br>(Do not fill the second one if your household registration has not been changed)
2. What type of household registration did you have before the age of 18?
A. Rural<br>B. Non-rural<br>C. Conversion from rural to non-rural. How old were you at that time? _____ years old<br>D. Conversion from non-rural to rural. How old were you at that time? _____ years old<br>E. In other cases, please specify ________________________
3. Where and how long had you lived in before the age of 18?<br>(Specific to the village for rural area and to the district and county for urban area; please fill in each area and years for multiple areas you lived in; less than 6 months need not to be counted, 8 months are counted as 8/12, and 1 year and 9 months are counted as 1+9/12)

Area 1: ______________________________

Years: ___. Area Type: 1. Rural 2. Town 3. Urban

Area 2: ______________________________

Years: ___. Area Type: 1. Rural 2. Town 3. Urban

Area 3: ______________________________

Years: ___. Area Type: 1. Rural 2. Town 3. Urban

Area 4: ______________________________

Years: ___. Area Type: 1. Rural 2. Town 3. Urban

Urbanization score: __________

(Urbanization score = 3 × years in urban + 2 × years in town + 1 × years in rural area; the score ranges from 18 to 54 before the age of 18)

4. Where and how long have you lived since the age of 18?

Area 1: ______________________________

Years: ___Area Type: 1. Country 2. Town 3. Urban

Area 2: ______________________________

Years: ___ Area Type: 1. Rural 2. Town 3. Urban

Area 3: ______________________________

Years: ___ Area Type: 1. Rural 2. Town 3. Urban

Area 4: ______________________________

Years: ___ Area Type: 1. Rural 2. Town 3. Urban

Part 3: Early events (before the age of 16 years)

1. Childhood trauma

See the Childhood Trauma Questionnaire - Short Edition

2. Were your parents divorced during your early life?

A. Never divorced

B. Yes, I lived mainly with my mother at that time. How old were you? _______ years old

C. Yes, I lived mainly with my father at that time. How old were you? _______ years old

D. Yes, I lived with both parents at that time. How old were you? _______ years old

E. Yes, they are not separated and I lived with them. How old were you? _______ years old

F. Yes, I lived with neither of the parents. How old were you? _______ years old

G. For other situations, please indicate ______________________

3. Did your natural parents die in the early years?

A. Parents are alive

B. My natural father died. How old were you at that time? ____ years old

C. My natural mother died. How old were you at that time? ____ years old

D. In other cases, please specify ______________________

Part 4: Living and medication habits
1. Which hand do you prefer to use chopsticks?
A. Left hand<br>B. Right hand<br>C. Both hands
2. Have you hurt yourself or someone else because of drinking?
A. No<br>B. Yes, in recent one year<br>C. Yes, but not in recent one year
3. Have you ever used sedative-hypnotics or over-the-counter sleep aids in the past one month?
A. No B. Yes
Do you have a regular use of drugs and symptoms will be worse when you stop to use?
A. No B. Yes

References

Chan KY, Zhao FF, Meng S, et al. Urbanization and the prevalence of schizophrenia in China between 1990 and 2010. World Psychiatry 2015; 14(2): 251-252.

Dye C. Health and Urban Living. Science 2008; 319(5864): 766-769.

Florian L, Peter K, Leila H, et al. City living and urban upbringing affect neural social stress processing in humans. Nature 2011; 474(7352): 498-501.

Mortensen PB, Pedersen CB, Westergaard T, et al. Effects of family history and place and season of birth on the risk of schizophrenia. New England Journal of Medicine 1999; 340(8): 603-608.

Paananen R, Ristikari T, Merikukka M, et al. Social determinants of mental health: a Finnish nationwide follow-up study on mental disorders. Journal of Epidemiology & Community Health 2013; 67(12): 1025-1031.

6.2 Childhood Trauma Questionnaire

(1) Introduction

The Childhood Trauma Questionnaire (CTQ) is used to assess childhood abuse experiences. The questionnaire consists of 28 items and 5 subscales: emotional abuse, physical abuse, sexual abuse, emotional neglect and physical neglect. The CTQ has been translated into Chinese and has shown good reliability and validity in Chinese.

(2) Procedures

(a) Instructions for participants

"This questionnaire surveys the life experience before the age of 16. Please choose the answer that suits best your situation based on your experience at that time. If you are not very clear, please try to choose the closest one. If you have any questions about questionnaire, please feel free to ask me for explanation. You do not have to worry about privacy leaks. We promise that your answers will remain confidential."

(b) Notes

The questionnaire includes personal privacy. During the test, the experimenter should not watch the participant filling in the questionnaire, but if the participant needs explanation, he/she should be able to provide timely assistance.

(3) Scoring (underlines represent reverse scoring)

(a) Emotional abuse: 3, 8, 14, 18, 25

(b) Physical abuse: 1, 9, 11, 12, 15

(c) Sexual abuse: **2**, 20, 21, 23, 24

(d) Emotional neglect: **5**, **7**, **13**, **19**, **28**

(e) Physical neglect: l, **2**, 4, 6, **26**

This questionnaire is a 5-point Likert scale (1, 2, 3, 4, 5, respectively represents never, occasionally, sometimes, often and always). ***The 2nd, 5th, 7th, 13th, 19th, 26th and 28th items need to be scored in reverse***. The scores of each subscale range from 5 to 25 points, with a total score of 25-125 points.

According to the scale manual, if the score of any subscale **exceeds the mid-to-severe traumatic exposure threshold**, he/she will be considered to have undergone childhood trauma of the corresponding type. In the western population, the threshold of each subscale of mid-to-severe trauma exposure is: emotional abuse ≥ 13 points, emotional neglect ≥ 15 points, sexual abuse ≥ 8 points, physical abuse ≥ 10 points, and physical neglect ≥ 10 points, but these thresholds have not been verified in the Chinese population so far.

References

Bernstein DP, Fink L. Childhood trauma questionnaire: A retrospective self-report: Manual. Psychological Corporation, 1998.

Zhao X, Zhang Y, Li L, et al. Reliability and validity of the Chinese version of childhood trauma questionnaire. Chinese Journal of Clinical Rehabilitation 2005, 9(20): 105-107.

Chapter 4 Quantitative environmental assessments

1. Geographic information collection

1.1 Geographic questionnaire

The geographic questionnaire is designed on an online questionnaire survey platform and is used to obtain personal information and the annual residential addresses of the participant since birth (Appendix 4.1). Personal information should be anonymized to protect the privacy of the participant, and the addresses should be transformed into the precise coordinates based on the Amap coordinate system (Figure 4.1).

In addition to the home address of each participant, we also record school or work address of the participant to truly reflect the impact of the environment on the participant. Specifically, the living address during the daytime is taken as the main address and the address at night as the second address. Take a non-resident student as an example, the school address is the main address and the home address is the second address. Both addresses could be used together or separately.

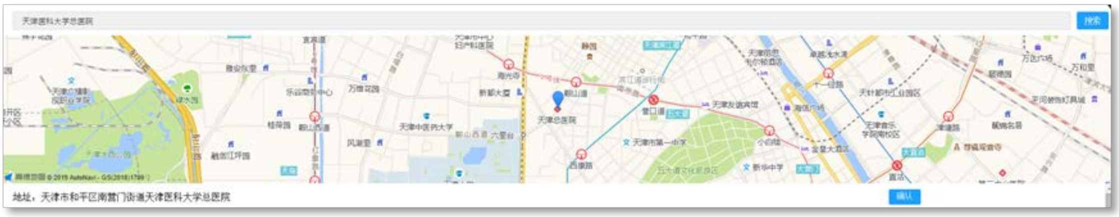

Figure 4.1 Address bar of the geographic information questionnaire

1.2 Geographic information collection

Participants should fully understand the purpose and procedures of geographic information collection before they fill in the questionnaire. The participants can use location-based apps on smartphones to enter the main page of the questionnaire by scanning the QR code, and fill the questionnaire (Figure 4.2). When filling in the address information, participants should search for the address of the corresponding time period on the electronic map to ensure that the address indicated by the blue cursor can accurately match their real address.

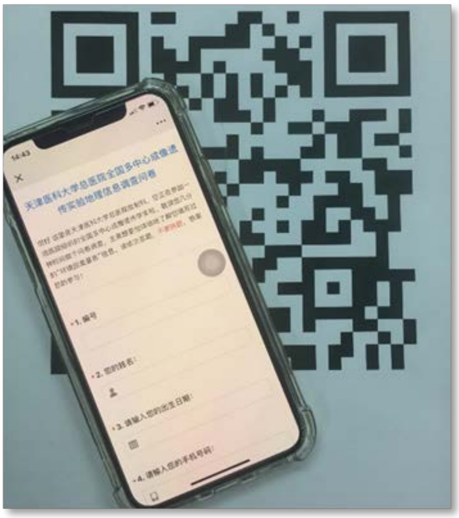

Figure 4.2 Geographic information page of the questionnaire

1.3 Geographic information processing

After the participant clicks the submit button, the platform will automatically receive and store the information, including the text of the participant's annual address (the main and second addresses) and the latitude and longitude coordinates based on Amap coordinate system. Subsequently, all coordinates were converted to match Google Earth coordinate system by using the GPSspg×Geocoding toolkit to simplify the whole process of environmental data extraction.

2. Environmental data processing

2.1 Environmental data retrieval

(1) Remote sensing data

Remote sensing data are collected from three databases of the Google Earth Engine, Social Economic Data and Applications Centre, and Resource Watch. These databases provide abundant remote sensing images and geographic data. The representative measures are shown in Appendix 4.2.

(2) National survey database

We obtained environmental data from the 1987-2016 China City Statistical Yearbooks, which are annual statistical publications reflecting the socioeconomic development of China cities. The representative socioeconomic measures are listed in Appendix 4.3.

Appendix 4.1

Geographic information questionnaire

ID		Name	
Date of birth		Cell phone number	

How many residential addresses (including home, rented house and apartment) did you live in since birth?

The continuous living time must be more than half a year. Please enter a single number from 1 to 10.

The changes of your residential addresses from birth to present.

Please provide all residential addresses and the years of beginning and ending (ages).

Address 1______________________________; Beginning year_______( ); Ending year_______( )

Address 2______________________________; Beginning year_______( ); Ending year_______( )

Address 3______________________________; Beginning year_______( ); Ending year_______( )

Address 4______________________________; Beginning year_______( ); Ending year_______( )

Address 5______________________________; Beginning year_______( ); Ending year_______( )

Did you live in school when you were a primary school student?

You should enter yes or no. If yes, the primary school address will be taken as the main address of that time period, and the second address will not be recorded; if no, the primary school address will be taken as the main address of the time period, and the home address as the second address.

Yes ( ); No ( )

How many primary schools did you study in?

The time for each school must be more than half a year. Please enter a single number from 1 to 5

The primary school addresses

Please provide all primary school addresses and the years of beginning and ending (ages).

Address 1______________________________; Beginning year_______( ); Ending year_______( )

Address 2______________________________; Beginning year_______( ); Ending year_______( )

Address 3______________________________; Beginning year_______( ); Ending year_______( )

Address 4______________________________; Beginning year_______( ); Ending year_______( )

Address 5______________________________; Beginning year_______( ); Ending year_______( )

Have you ever studied in a junior high school?

You should enter yes or no. If yes, please fill the next question; if no, please turn to the last two questions.

Yes ( ); No ( )

Did you live in school when you were a junior high school student?

You should enter yes or no. If yes, the junior high school address will be taken as the main address of the time period, and the second address will not be recorded; if no, the junior high school address will

be taken as the main address of the time period, and the home address as the second address.

Yes (   ); No (   )

How many junior high schools did you study in?

The time for each school must be more than half a year. Please enter a single number from 1 to 5

The junior high school addresses

Please provide all junior high school addresses and the years of beginning and ending (ages).

Address 1______________________________; Beginning year________( ); Ending year________( )

Address 2______________________________; Beginning year________( ); Ending year________( )

Address 3______________________________; Beginning year________( ); Ending year________( )

Address 4______________________________; Beginning year________( ); Ending year________( )

Address 5______________________________; Beginning year________( ); Ending year________( )

Have you ever studied in a senior high school or a technical secondary school?

You should enter yes or no. If yes, please fill the next question; if no, please turn to the last two questions.

Yes (   ); No (   )

Did you live in school when you were a senior high school (technical secondary school) student?

You should enter yes or no. If yes, the senior high school (technical secondary school) address will be taken as the main address of the time period, and the second address will be not recorded; if no, the senior high school (technical secondary school) address will be taken as the main address of the time period, and the home address as the second address.

Yes (   ); No (   )

How many senior high schools (technical secondary schools) did you study in?

The time for each school must be more than half a year. Please enter a single number from 1 to 5

The senior high school or technical secondary school addresses

Please provide all school addresses and the years of beginning and ending (ages).

Address 1______________________________; Beginning year________( ); Ending year________( )

Address 2______________________________; Beginning year________( ); Ending year________( )

Address 3______________________________; Beginning year________( ); Ending year________( )

Address 4______________________________; Beginning year________( ); Ending year________( )

Address 5______________________________; Beginning year________( ); Ending year________( )

Have you ever studied in a university (junior college, higher vocational college)?

You should enter yes or no. If yes, please fill the next question; if no, please turn to the last two ones.

Yes (   ); No (   )

The university, junior college or higher vocational college addresses

If you are still studying in the school, the ending time will be filled in the current date.

Please provide the address and the years of beginning and ending (ages).

Address______________________________; Beginning year________( ); Ending year________( )

Have you ever been a postgraduate?

You should enter yes or no. If yes, please fill the next question; if no, please turn to the last two questions.
Yes ( ); No ( )
**The university address**<br>If you are still studying in the school, the ending time will be filled in the current date.<br>Please provide the address and the years of beginning and ending (ages).
Address______________________________; Beginning year_______( ); Ending year______( )
**Have you ever been a doctoral student?**<br>You should enter yes or no. If yes, please fill the next question; if no, please turn to the last two questions.
Yes ( ); No ( )
**The university address**<br>If you are still studying in the school, the ending time will be filled in the current date.<br>Please provide the address and the years of beginning and ending (ages).
Address______________________________; Beginning year_______( ); Ending year______( )
**How many different work addresses did you have after graduation?**<br>The time span of work must be more than half a year. If the work address is changeable, then choose the option of 'work address changeable'. If you don't have a job after graduation, you will choose the option of 'unemployment'.<br>Please enter a single number from 1 to 10. If you select any option of 1-10, we shall take the work address as the primary address of the time span. If your work address is changeable or you are unemployed, the home address of the time span will be taken as the primary address, and the secondary address will not be recorded.
No. of work addresses ( ); Work address changeable ( ); Unemployment ( )
**The changes of your work addresses after graduation.**<br>Please provide all work addresses and the years of beginning and ending (ages).
Address 1______________________________; Beginning year_______( ); Ending year______( )<br>Address 2______________________________; Beginning year_______( ); Ending year______( )<br>Address 3______________________________; Beginning year_______( ); Ending year______( )<br>Address 4______________________________; Beginning year_______( ); Ending year______( )<br>Address 5______________________________; Beginning year_______( ); Ending year______( )

Appendix 4.2

Representative environmental measures from remote sensing data

Representative environmental measures	Fields
Normalized difference vegetation index (NDVI)	Landform and topography
Normalized difference snow index (NDSI)	Landform and topography
Normalized difference water index (NDWI)	Landform and topography
Land cover	Landform and topography
Elevation	Landform and topography
Roads	Urbanization
Population grid	Urbanization
Population density	Urbanization
Built-up grid	Urbanization
Settlement grid	Urbanization
Nighttime lights	Urbanization
Surface temperature	Climate
Air temperature	Climate
Air relative humidity	Climate
Soil moisture	Climate
Total cloud cover	Climate
Solar radiation	Climate
Wind speed	Climate
Standard precipitation index	Climate
Aridity	Climate
Vapor pressure	Climate
PM 10	Air quality
PM 2.5	Air quality
SO_2	Air quality
CO	Air quality
NO_2	Air quality
O_3	Air quality

Appendix 4.3

Representative environmental measures from national survey data

Representative environmental measures	Fields
Per capita gross domestic product (GDP)	Economy
Per capita volume of freight transportation	Economy
Per capita volume of passenger transportation	Economy
Per capita volume of freight transportation	Economy
Per capita industrial electricity consumption	Economy
Population density	Urbanization
Per capita area of paved roads	Urbanization
Green coverage rate in built-up areas (%)	Urbanization
Per capita expenditure	Living condition
Per capita income	Living condition
Per capita savings	Living condition
Per capita private cars	Living condition
Per capita cell phones	Living condition
Per capita internet customers	Living condition
Per capita living areas	Living condition
Hospitals per 10000 people	Healthcare
Hospital beds per 10000 people	Healthcare
Doctors per 10000 people	Healthcare
Per capita expenditure for education	Education
Primary schools, middle schools and universities per 10000 people	Education
Teachers per 10000 students in these schools	Education
Average students per school	Education
Per capita expenditure for science and technology	Science and technology
Number of applied invention patents per 10000 people	Science and technology
Number of authorized invention patents per 10000 people	Science and technology

Chapter 5 Blood samples

1. Blood sample collection

1.1 Blood sample collection with EDTA tubes

1.1.1 Materials

(1) Ethylene diamine tetra-acetic acid (EDTA) tube and lavender lid (5ml×2, A and B); (2) butterfly needle; (3) vacutainer needle holder; (4) small plasters; (5) tourniquet; (6) alcohol wipes; (7) cotton wool; (8) sterile gloves; (9) sharps bin; and (10) ice packs (optional).

Notes: The collected blood samples should be centrifuged within 2 hours at room temperature (20-25℃), and the samples after centrifugation should be placed in an ultra-low temperature environment (-80℃) in time. If long-distance transportation is required, the samples can be temporarily placed in a 4℃ environment (refrigerated box and ice pack) and should be centrifuged within 4-6 hours.

1.1.2 Procedures for collection

(1) The experimenter labels the EDTA tubes with the date, participant's ID and name.

(2) After checking the labelling information on EDTA tubes, blood samples are collected, and then EDTA tubes should be gently inverted at least 10 times.

(3) The quality of the blood sample is checked by observing the color of the sample. The transparent yellow indicates bilirubin; milky white and turbid indicates chylomicron; clear red indicates hemolysis; and blood clot means coagulation. In these situations, the blood sample should be re-collected.

(5) For the qualified blood samples, the experimenter records the name, participant's ID, gender, date and time of blood collection on the "blood sample registration form" for subsequent operation and management.

1.1.3 Centrifugation

(1) Centrifuge parameters: a horizontal rotor at 2000 rpm for 20 min at 20-25℃.

(2) After centrifugation, there are three layers in the EDTA tube: plasma (upper layer), buffy coat (white blood cells, middle layer) and red blood cells (bottom layer)

(Figure 5.1).

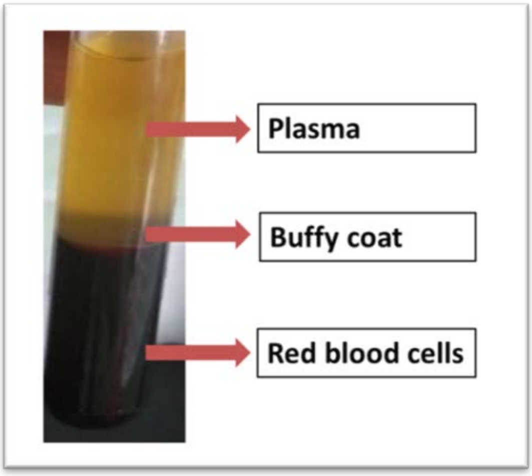

Figure 5.1 Three layers in EDTA tube after centrifugation

1.1.4 Labeling

(1) Materials: Sterile screw-cap cryotubes (0.5ml×12) and blood sample labels × 12 (8 for plasma, 4 for white blood cells).

(2) Twelve cryotubes are prepared for each participant.

(3) The experimenter should label the sterile screw-cap cryotubes after checking the participant's ID (Figure 5.2).

Note: The ID of the tube body should be the same as that of the lid.

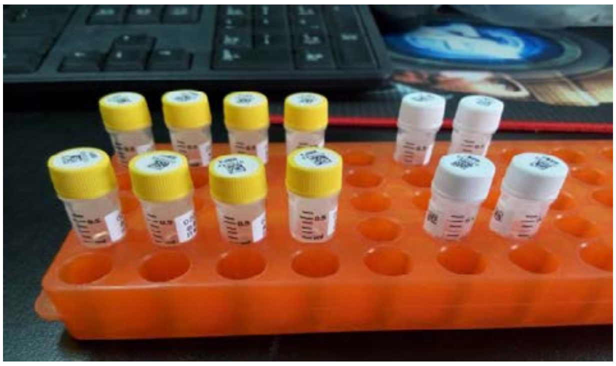

Figure 5.2 Labeled cryotubes

1.1.5 Isolation

(1) Materials: Test tube rack, cryotubes (0.5ml×12), and disposable straw (3ml).

(2) The following steps should be conducted for one 5ml EDTA tube.

(a) The experimenter wears medical sterile gloves and mask.

(b) Before isolating, the experimenter should confirm that the tubes are labeled and

are in an appropriate position. The type of the sample can be distinguished by the color of lid (Figure 5.3). Please place the cryotubes on the test tube rack in order and orient the scale surface to the experimenter.

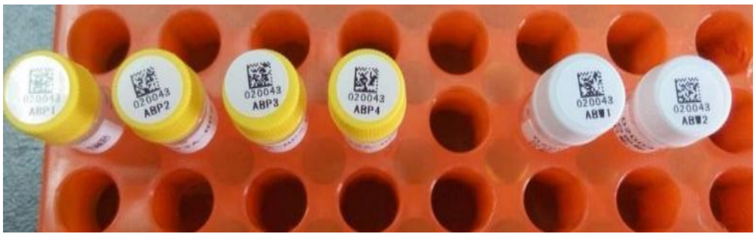

Figure 5.3 Cryotubes on the test tube rack in order

(c) The blood sample will be taken out of the centrifuge and placed on the test tube rack. Shaking should be avoided during the process.

(d) Please slowly rotate and remove the EDTA tube lid to avoid damage of blood layers caused by excessive force.

(e) The upper layer of plasma will be drawn and isolated into 4 yellow-lid cryotubes (0.5ml). Please keep a small amount of plasma to prevent the underlying buffy coat from being drawn. Pinch the straw before drawing to prevent damage of the buffy coat (Figure 5.4). After the straw entering the EDTA tube, it can only be sucked out, must not drop back.

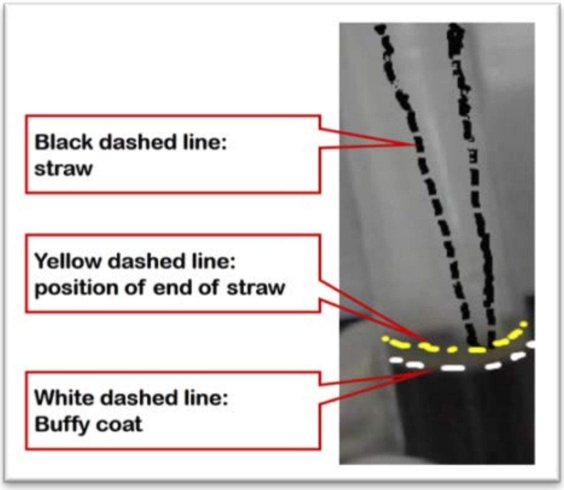

Figure 5.4 Correct position of the straw tip

(f) The experimenter will collect the buffy coat with straw moving around and assign it into 2 white-lid cryotubes (0.5 ml) drop by drop (Figure 5.5). Please collect a

small amount of red blood cells to ensure that the buffy coat layer is completely collected.

(g) Please discard the used tubes into bio-waste bags to avoid environmental pollution and cross-contamination.

(h) The experimenter will repeat the above procedures for another EDTA tube.

(i) Please check whether the labels of 12 cryotubes are consistent.

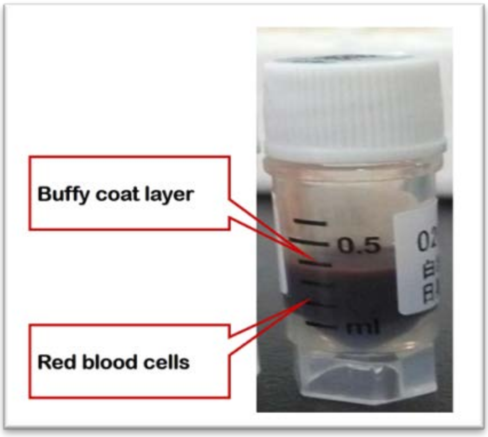

Figure 5.5 White-lid cryotube

Notes

(a) The experimenter should confirm the informed consent to protect the rights of participants.

(b) The experimenter should carefully check the labelling information to ensure the consistency between labels and cryotubes.

(c) Blood sample collection should be performed after behavioral assessment and MRI data collection.

(d) The straw is disposable which must not be reused.

(e) Please pay attention to the safety protection during the collection and processing of blood samples to avoid pollution.

(f) The waste tubes should be treated as medical waste.

(g) The appropriate disinfectants should be used to clean spilled specimens.

(h) Empty stomach is not necessary for the participant.

(i) Please complete the management system even if collection or isolation is failed.

(j) Please confirm if the buffy coat is successfully isolated.

1.2 Blood sample collection with RNA tubes

1.2.1 Materials

(1) PAXgene® blood RNA tube; (2) a "discard tube" if the PAXgene® Blood RNA tube is the only tube being drawn; (3) labels; (4) butterfly needle and vacutainer needle holder; (5) small plasters; (6) tourniquet; (7) alcohol wipes; (8) cotton wool; (9) sterile gloves; and (10) sharps bin.

1.2.2 Sample collection

(1) The PAXgene® blood RNA tube should be stored at 18-25°C prior to use and should be labeled with the participant's ID (Figure 5.6).

(2) The PAXgene® blood RNA tube should be drawn at the end of the venous blood collection. If the PAXgene® blood RNA tube is the only tube to be drawn, 1-2 ml blood sample should be drawn into a "discard tube" prior to drawing blood into the PAXgene® blood RNA tube.

(3) Please set aside enough time for a complete blood collection. Please ensure that the blood has completely flowing into the tube before removing it from the holder.

(4) Please gently invert the PAXgene® RNA tube 8-10 times after blood collection.

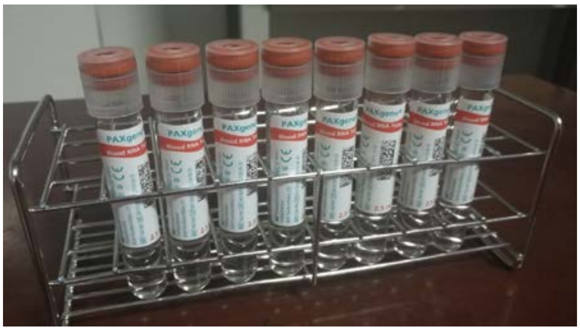

Figure 5.6 PAXgene® blood RNA tubes

2. Storage

2.1 The EDTA tubes

2.1.1 Materials

(1) Ultra-low temperature freezer (-86°C)

(2) Freeze-proofing gloves

(3) Colored cryopreservation boxes and waterproof labels

2.1.2 Operation procedures

(1) The experimenter should label the colored cryopreservation boxes with the center ID, sample type, box number and date.

(2) Please place all cryotubes upright in a cryopreservation boxes (10×10) with different colored boxes for plasma (yellow) and buffy coats (white) (Figure 5.7).

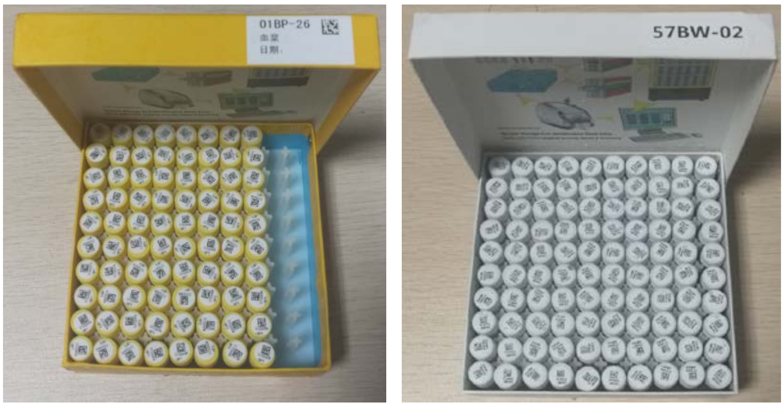

Figure 5.7 10×10 cryopreservation boxes

(3) Please put the boxes into respective locations in freezers at -80°C (Figure 5.8).

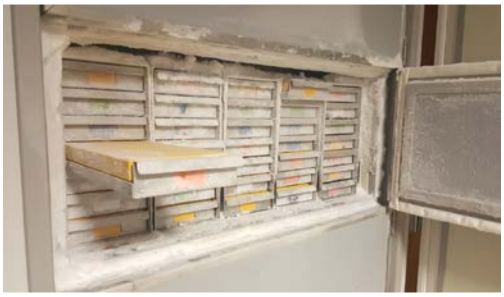

Figure 5.8 Ultralow temperature freezer

(4) The experimenter should complete the management information of the blood sample (Figure 5.9).

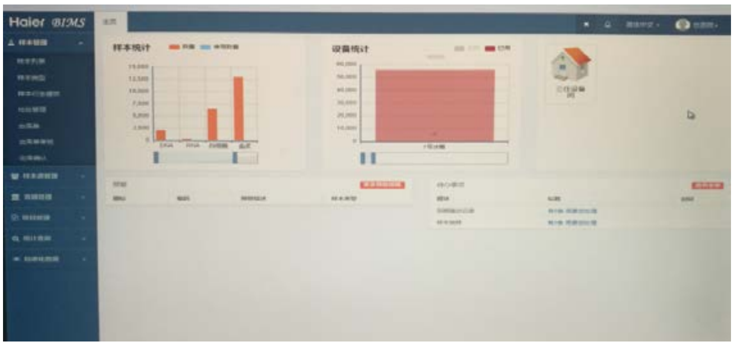

Figure 5.9 Biobank management system

Notes

(a) The cryotubes of the same participant should be placed in correct cells and boxes and stored in separate freezers.

(b) Please complete the management information of blood sample in time.

(c) During the storage of the sample, the experimenter should pay attention to the temperature of the freezer, and act quickly when moving the box to ensure the safety temperature of the sample.

(d) Avoid moving the biological samples in and out for many times.

2.2 The RNA tube

(1) The PAXgene® Blood RNA tube should be placed upright at room temperature (18-25°C) for 2-72 hours before transferring to freezer at -20°C.

(2) The PAXgene® Blood RNA tube should be temporally placed in freezer at -20°C for 24 hours.

(3) The PAXgene® blood RNA tubes should be long-term stored in freezers at -80°C (Figure 5.10).

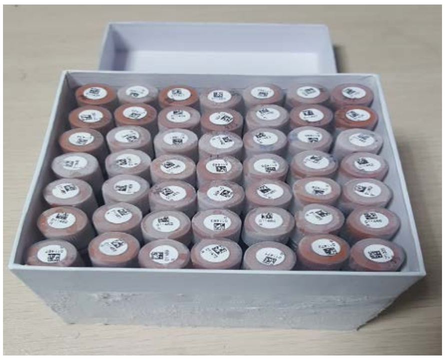

Figure 5.10 RNA tubes in a cryopreservation box

3. Transportation

The blood samples will be finally transported to the TMUGH by a professional biomedical cold chain logistics company for centralized managements and DNA extraction.

3.1 Pipeline

The pipeline for the transportation of the blood samples from other centers to the TMUGH is shown in Figure 5.11.

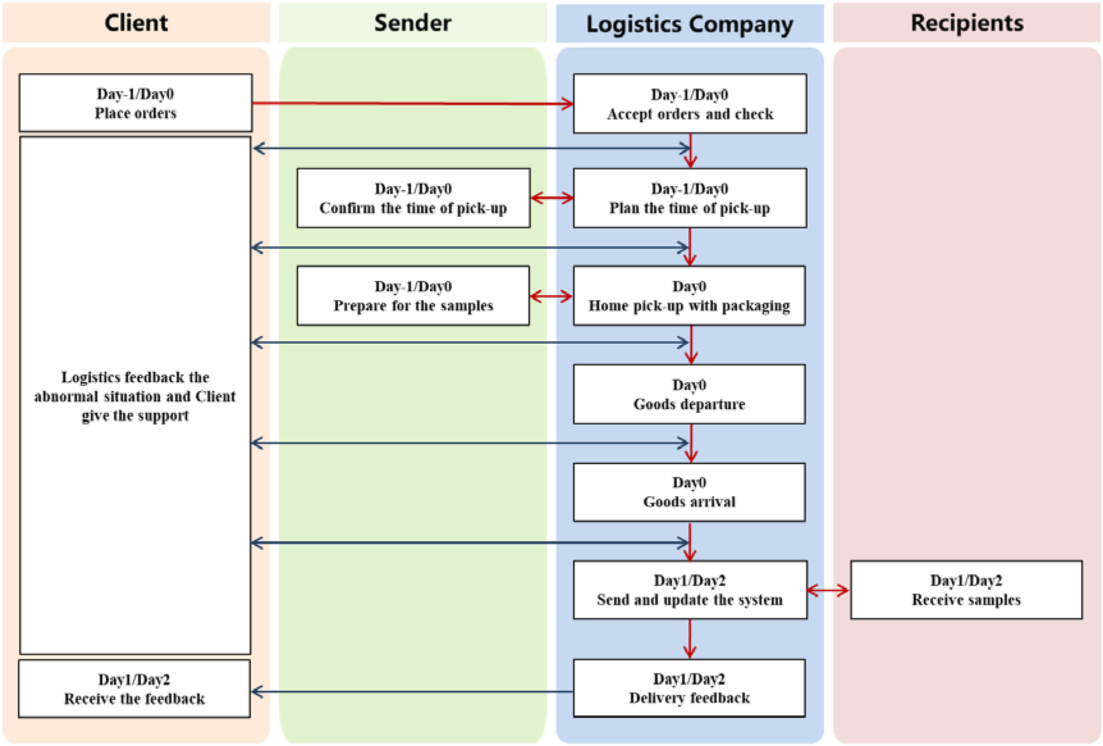

Figure 5.11 Pipeline for blood sample transportation

Note: Day-1 is the day before pickup; Day0 is the day of pickup; Day1 is the day after pickup; Day2 is two days after pickup. The double arrow line represents the interactive communications; the single arrow line represents the direction.

3.2 Key points

3.2.1 Key points for pickup

(1) The researchers of the branch center should prepare the blood samples for transportation.

(2) The staff of the logistics company should prepare the relevant packaging materials (including the packing box, refrigerant, waybill, gloves, thermometer, foam rack, and filler) after receiving the pick-up orders.

(3) According to the appointment time, the staff of the logistics company should contact the researcher of the branch center 30 min before picking up.

(4) The staff of the logistics company should arrive on time to check the prepared packaging of goods and labels and to assist the researcher to perform packaging and turn on the thermometer.

(5) The staff of the logistics company should complete the photo recording. Please load and unload the package with care.

(6) They should complete handing over and fill in the waybill and related documents.

(7) After the pickup is completed, the goods status and information will be reported to the project customer service staff.

3.2.2 Key points for sending and receiving

(1) The staff of the logistics company should check the packaging situation as soon as the goods are picked up and notify the consignee of the TMUGH one hour in advance to prepare the reception.

(2) Please load and unload with care during the whole process.

(3) The staff of the logistics company should assist the consignee to check the temperature at the reception, to read the temperature data, and to turn off the thermometer in time.

(4) The consignee of the TMUGH should give a feedback to the branch center after the handover is completed.

3.2.3 Accident handling

We have established a team composed of the general manager, quality control manager, dispatch manager and other related personnel to deal with accident.

(1) Loss of goods

The team should analyze the possible location of the loss, and discuss about the solution to find back. The dispatch manager should feedback the information of the goods to the client.

(2) Broken packaging

The team will check the cargo safety situation and feedback to the quality control department for biological samples and the dispatching department immediately, the quality control department gives a solution, the manager of the dispatching department will feedback problems to the customer in time.

(3) Abnormal temperature

The team will analyze causes for temperature anomalies and provide solutions. The dispatching department should timely report the situation of the goods to the customer, and the operation department should wait for the customer's instruction for further action.

(4) Transportation delay

The staff of the logistics company should notify the dispatch manager and quality control manager and report reasons for the delay. The goods should be delivered to the designated location in a timely manner and the delay information should be fed back to the customer.

3.2.4 Emergency plan

We have made an emergency plan covering the whole process of the transportation. For any sudden problems in transportation, the emergency plan is activated according to the manual.

3.2.5 Safety training

(1) Operational training

The process of packaging, pick-up, dispatch and accident handling must be completed by a professionally trained logistics specialist.

(2) Personnel safety

Logistics specialists are required to wear medical safety gloves during operation.

(3) Professionalism

The staffs should wear uniform dress, speak politely, and operate professionally.

(4) Quality monitoring

Regular quality assessments are used to eliminate potential safety hazards.

3.3 Sample packaging program

3.3.1 Preparation of dry ice and cargo

Enough granular dry ice should be prepared and the cryopreservation box should be fastened by rubber bands (Figure 5.12).

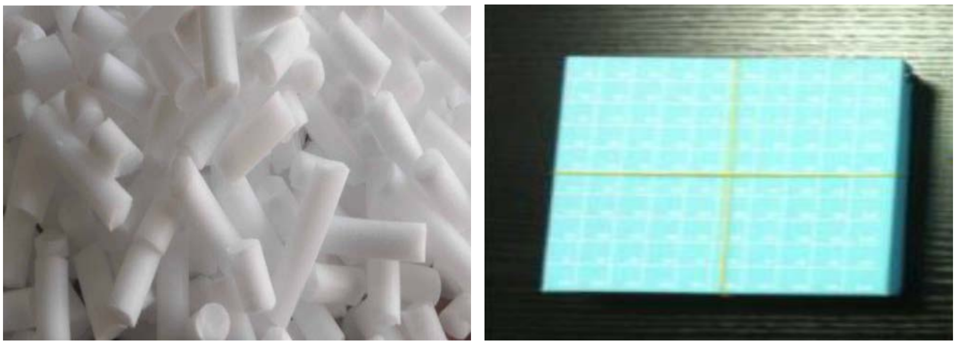

Figure 5.12 Granular dry ice and fastened cryopreservation box

3.3.2 Packaging work

The staffs put the fastened cryopreservation box into the insulated cold chain box and pour dry ice of more than 5 kg at a constant speed (Figure 5.13). The probe of the thermometer is inserted from the probe hole into the dry ice in the box, and the thermometer is placed in a fixed position (Figure 5.14). Be sure to wear gloves and do not touch dry ice directly to prevent frostbite.

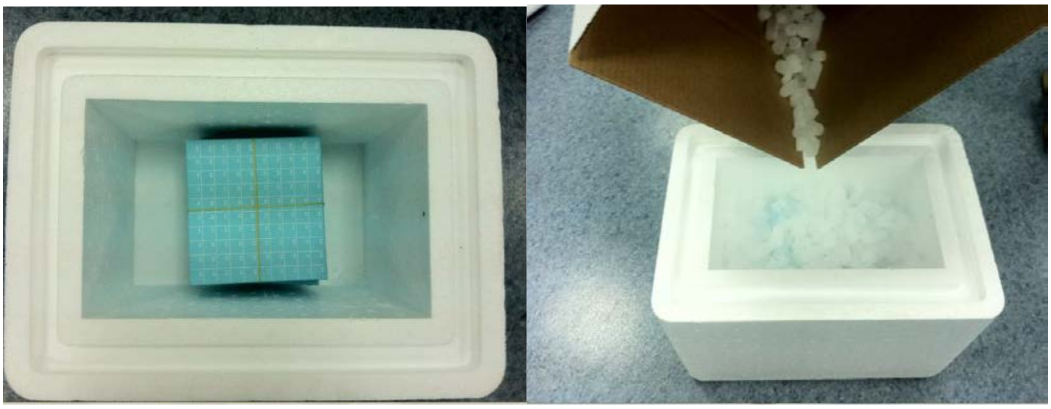

Figure 5.13 Packaged box

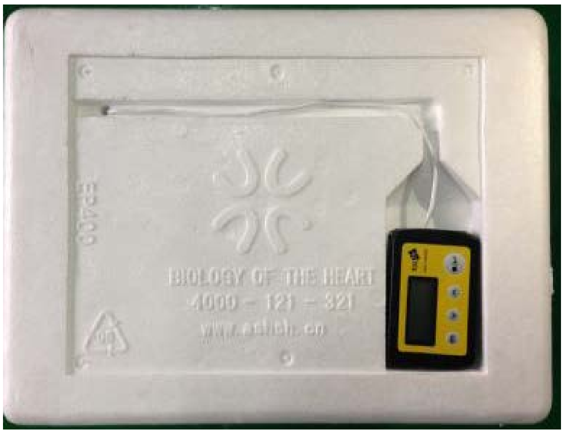

Figure 5.14 Position of the thermometer probe

3.3.3 Sealing work

The insulation box is sealed with tape; the transport order and the sample transit receipt are packed in the self-sealing bag and are placed on the EP box and glued with tape. Please check and ensure that the samples, documents, and waybills are complete, and seal the outer carton with sealing tape.

3.4 Data feedback and traceability

3.4.1 Data feedback

(1) The temperature data could be read on site, and the temperature data should be uploaded to the biological management system within 72 hours.

(2) As VIP customers, we have the permission to enter the internal system and can self-check and download these data in batches (Appendix 5.1).

(3) After we have signed the waybill and taken photos on the spot, they will be uploaded to the system on the same day.

(4) After the delivery is completed, the customer service personnel should promptly feedback the information to the client and other relevant personnel.

3.4.2 Traceability

For the transportation-related waybills and temperature records, the electronic files are kept for 5 years, and the paper files are kept for 1 year.

4. DNA and RNA extraction and storage

4.1 DNA extraction and storage

4.1.1 DNA extraction

(1) Materials and equipment for DNA extraction

QIANGEN® nuclearase free water; AXYGENE® kit; nanodrop 2000; agarose gel (1%); electrophoresis; EP tubes; and centrifuge.

(2) DNA extraction steps

(a) Add 500ul Buffer AP1 to an empty 1.5 ml EP tube.

(b) Add 250ul of anticoagulated whole blood.

(c) Add 100ul Buffer AP2, vortex for 10s, centrifuge at 12,000 × g for 10min.

(d) Carefully move the supernatant into the preparation tube and place the preparation

tube in a 2 ml centrifuge tube, centrifuge and wash the tube with Buffer W1A and Buffer W2, and finally elute the genomic DNA with nuclease-free ultrapure water.

(e) After DNA extraction, the information of the center, quantity, time, and signature of the sampler should be recorded.

4.1.2 Storage

The extracted DNA-containing EP tube is marked with a marker pen and returned to its corresponding position in ultralow temperature freezer.

4.2 RNA

The blood samples for RNA sequencing are only collected for 700 participants who have participated in this study recently. After a short time (within 6 months) of storage in the PAXgene® Blood RNA tube at -80°C, the extraction and sequencing of RNA will be performed by a professional sequencing company.

Appendix 5.1

Shipment information of a representative case

Basic information	
Name	
Note	
Model	WS-711LPRO
Serial number	A0111017
Recording interval	00:10:00
Recording time	2 days 05:40:00
Alarm upper limit	Temperature: 70°C
Alarm lower limit	Temperature: -86°C
Starting time	2014-12-03 11:51:04
Stop time	2014-12-05 17:31:04
**Recording time**	
Record maximum	Temperature: -41.5°C
Record minimum	Temperature: -79°C
Record average	Temperature: -78°C
Upper limit alarm	No trigger
Lower limit alarm	No trigger
**Recording curve**	

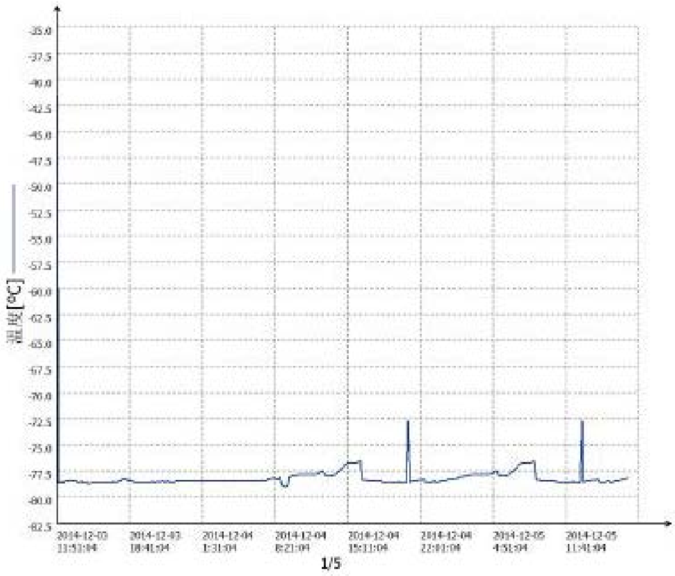

Chapter 6 Genotyping

1. Infinium® ASA

1.1 Introduction

The Illumina Infinium Asian Screening Array (ASA) is an advanced whole genome genotyping array that is designed according to data from whole genome sequencing (WGS) of over 9,000 samples, including six East Asian populations. The ASA covers the maximum low-frequency variants in East Asian populations and provides a cost-effective solution for genetic studies, variant screening, and precision medicine research in these populations. The ASA used the iScan system, integrated analysis software and Infinium high-throughput screening (HTS) assay to provide optimized modules for genome-wide association studies (GWAS). These modules include common, low-frequency and rare variants, and those associated with biological functions, human diseases, pharmacogenomics, human lymphocyte antigen (HLA), and loss of function (LOF) exome in East Asian populations. Specifically, there are over 650,000 markers, including 446,040 genome-wide backbone markers, 122,303 clinical research markers, 48,364 Asian discovery panel (ADP) markers, 7,982 quality control markers, and 100,000 custom markers.

1.2 Array markers

(1) Genome-wide backbone markers (n = 446,040)

(a) Maximized imputation accuracy and genomic coverage

(b) 9,000 Asian whole genomes with 15x/30x coverage

(2) Clinical research markers (n = 122,303)

(a) Known disease markers

(b) Pharmacogenomics' markers

(c) HLA markers

(d) LOF exome markers

(e) ASA KOL submissions

(3) ADP markers (n = 48,364)

(a) Phenotype agnostic markers

(b) Asian specific LOF exome markers

(4) Quality control markers (n = 7,982)

(a) Markers for sample tracking and stratification

(b) Asian focused panel of AIMS

1.2.1 Genome-wide backbone markers

Genome-wide backbone markers were selected using cross-population algorithm for maximizing imputation accuracy and genomic coverage by using the largest whole genome reference with high-performance imputation tag SNPs and best coverage of the low-frequency variants in Asian populations. This module includes 241,126 common markers (MAF ≥ 5%), 159,169 low-frequency markers (MAF = 1-5%), 45,745 rare markers (MAF ≤ 1%), and 24,854 markers outside of the 1000 genomes project phase1 (KGP1). The module also provides over 70,000 novel markers from ASA consortia to enable discoveries in East Asia.

1.2.2 Clinical research markers

This module provides markers associated with human diseases, including 37,955 common markers, 13,663 low-frequency markers, 28,181 rare markers, and 42,504 markers outside of the KGP1.

(1) ClinVar known and actionable information

Markers in this category represent a list of pathogenic or likely pathogenic variants. More than 35,000 variants are clinically annotated and over 13,000 markers are detected in 7,175 diseases, including cancer, cardiovascular diseases, neurological diseases, etc. Additionally, more than 18,000 variants are chosen from the gene list of the American College of Medical Genetics and Genomics (ACMG).

(2) Pharmacogenomic variants

These markers include 4,456 annotated variants in PharmGKB (July 2018), of which 1,638 variants are associated with ADME phenotypes and 706 variants are assigned to star allele haplotypes. Furthermore, 13,905 variants from ADME4 and CPIC were chosen for imputation and overall gene coverage.

(3) Clinical exome

This category was designed for capturing potentially important LOF exome variants to discover functional variants from the exomes of the clinically relevant genes. These markers include 8,624 exome variants present in East Asian populations in the genome aggregation database, and 87,850 unique exome variants.

(4) Potentially associated variants from the GWAS literature

There were 13,926 variants from the GWAS literature (VaDE/NHGRI), including GWAS significant variants for common diseases in Asian populations.

(5) HLA and MHC

This category was designed for immune system research. According to over 11,000 HLA Asian reference samples, 7,179 MHC imputation tags were chosen based on iterative imputation process. Thus, the ASA can yield high imputation accuracy for HLA-classic and non-classic alleles.

1.2.3 ADP markers

This category contains 19,820 common markers, 2,901 low-frequency markers, 19,390 rare markers, and 6,253 markers outside of the KGP1.

(1) Research inspired variants for discovery

This category consists of 20,000 markers that were discovered with targeted Illumina arrays, including Infinium Immuno Array (10,388 markers), Infinium NeuroX Consortium Array (8,701 markers), Infinium Drug Dev Array (6,985 markers), Infinium Psych Array (2,616 markers), and LOF exome in East Asians (19,912 markers).

(2) A priori LOF variants

These markers include over 20,000 variants that can predict loss of function in the WGS samples.

1.2.4 Quality control markers

This category consists of 7,982 variants that can be used for sample tracking, quality control and stratification, including ancestry markers (2,562), sex markers (2,444), blood markers (1,859), mitochondrial markers (1,042), human linkage markers (833),

pseudo autosomal region (676), fingerprinting markers (428), forensic seq markers (5).

1.2.5 Sex chromosome coverage

This category consists of over 25,000 variants on X, Y and MT, including clinical and GWAS associated variants, sex determination markers, and Y- and MT-haplotype calling markers.

1.2.6 Custom markers

This category includes about 50,000 variants customized by Broad Institute and about 40,000 variants customized by Asian research collaboration.

References

Infinium® Asian Screening Array (ASA):

https://support.illumina.com.cn/array/array_kits/infinium-asian-global-screening-array.html

The 1000GenomesProject: www.1000genomes.org

ClinVar Database: www.ncbi.nlm.nih.gov/clinvar.

ACMG recommendations for reporting of incidental findings in clinical exome and genome sequencing: www.ncbi.nlm.nih.gov/clinvar/docs/acmg/.

PharmaADME Gene List: www.pharmaadme.org.

2. Genotyping

2.1 Overview

The genome sequencing of the CHIMGEN study is performed by a professional sequencing company. The whole process is described as follows:

2.2 Genome sequencing company and facilities

The professional sequencing company has established a high-quality gene sequencing platform and a high-performance computing platform, which effectively supports the needs of big data analysis and storage. The company has advanced devices including the NovaSeq, PacBio Sequel, HiSeq X, HiSeq 4000/2500/2000, Life Ion Proton, iScan System Integrated chip barcode scanning, and DNA chip identification scanning analysis system.

2.3 Sample transfer and reception

2.3.1 Sample transfer

The sample tubes will be transferred from the TMUGH to the company by cold chain transportation. The tubes should be closed during the transportation and placed vertically upwards. In addition, the vibration should be avoided.

2.3.2 Sample reception

The samples will be collected and checked by the source sample group of the company. The samples were checked at 4 °C. The standard includes:

(1) There is no empty or damaged tube.

(2) The tubes should be sealed and have clear and complete labels without shedding.

(3) Each sample should correspond to a unique ID and is placed according to the arrangement of the company.

(4) After the inventory is completed, the sample is stored in -20 °C refrigerators.

2.4 Sample storage

The sample tubes are stored in freezers at -20 °C before genotyping. The freezers are managed by person in charge, and the blood samples are forbidden to be taken out to prevent sample loss.

2.5 Sample normalization

2.5.1 Preparation

After quality control, the concentration of all samples will be uniformly adjusted to about 50ng/μl. In addition to the hands-on sample, about 1μg of each sample was taken and stored in 96-plate for subsequent genotyping. The remaining samples are stored in one tube per sample.

2.5.2 Experimental procedures

The Illumina Infinium HTS Assay is used for genotyping. The main procedures include amplifying DNA, fragmenting the amplified DNA samples, precipitating DNA, resuspending DNA, hybridizing to BeadChip, washing BeadChip, extending and staining BeadChip, and imaging BeadChip.

2.5.3 Blind test

The blind test repetition rate should be more than 99%. If the detection rate of any

sample is less than 97%, the sample needs to be genotyped again. Moreover, samples from the ASA chip that contains unqualified sample should be re-genotyped again to ensure the call rate.

2.5.4 Remaining samples

The remaining samples will be recorded and stored in freezers at -20 °C in the company, and the sample location should be recorded in the management system of the company. The samples will be saved until they are sequenced again or returned to the TMUGH. If the samples need to be returned, the sample list and the address should be submitted to the company by the customer. The samples should be counted by the sample return group of the company and transported to the sample receiving address by cold-chain transportation.

2.5.5 Data delivery

The returned data include the raw chip data, plink format data, and CNV results.

3. Imputation

Imputation aims to predict genotypes that are not directly genotyped with a reference panel. The imputation is typically divided into two steps: pre-phasing and imputation. In this study, pre-phasing will be carried out using SHAPEIT2 in chunks of 5,000 kb with an overlap of 250 kb between chunks. Then, imputation will be done with the IMPUTE2 with merged database of 1000 Genomes project phase 3 and hundreds of sequenced Chinese Han individuals (provided by the company) as the imputation reference panels.

References

Anderson CA, Pettersson FH, Clarke GM, et al. Data quality control in genetic case-control association studies. Nature protocols 2010; 5(9): 1564-1573.

SHAPEIT2: https://mathgen.stats.ox.ac.uk/genetics_software/shapeit/shapeit.html

IMPUTE2: http://mathgen.stats.ox.ac.uk/impute/impute_v2.html

Chapter 7 Neuroimaging data

1. Brain MRI data collection

1.1 Overview

The brain MRI data were acquired by 3.0-Tesla scanners. The high-resolution T1-weighted structural magnetic resonance imaging (sMRI), diffusion tensor imaging (DTI) and resting-state functional MRI (rs-fMRI) were acquired in all centers, and diffusion kurtosis imaging (DKI) and arterial spin labeling (ASL) were acquired in more than a half of the centers. After the completion of MRI safety questionnaire, each participant was scanned in the order of the following sequences (Table 7.1).

Table 7.1 Order and timing of scanning sequences (TMUGH)

Scan sequences	Duration (s)
1. Localizer	7
2. Shimming (optional)	5
3. T2-weighted imaging (T2WI)	78
4. Resting-state functional magnetic resonance imaging (rs-fMRI)	370
5. Three-dimensional T1-weighted structural MRI (sMRI)	259
6. Diffusion tensor imaging (DTI)	420
7. Diffusion kurtosis imaging (DKI) (optional)	336
8. Three-dimensional arterial spin labeling (ASL) (optional)	293
Total time	1768

1.2 MR scanners

Brain MRI data were acquired by 3.0-Tesla scanners from General Electrics, Siemens and Philips. To reduce the influence of MRI scanners from different manufactures, we encouraged centers to acquire data using the MR 750 scanner of General Electrics for its relatively high image quality and popularity in China at that time. The numbers and types of MR scanners used in this study are showed in Figure 7.1.

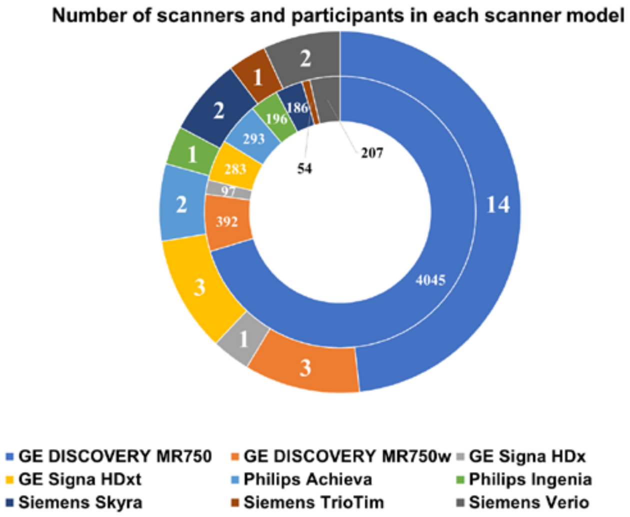

Figure 7.1 Number of scanners and participants in each scanner model

1.3 Standard parameters for each MR scanner

The standard parameters of the five sequences for different MR scanners are shown in Table 7.2, Table 7.3, Table 7.4, Table 7.5, and Table 7.6.

2. MRI data transfer and storage

The DICOM images of MRI data, behavioral and environmental data should be saved in a folder named by each participant's ID (Figure 7.2). This directory should include the following subfolders: (1) 3DT1 stores structural MRI data; (2) REST stores resting-state functional MRI data; (3) DTI stores diffusion tensor imaging data; (4) DKI stores diffusion kurtosis imaging data if the DKI data are acquired; (5) ASL stores arterial spin labelling data if the ASL data are acquired; and (6) COGN stores raw behavioral and environmental assessment data.

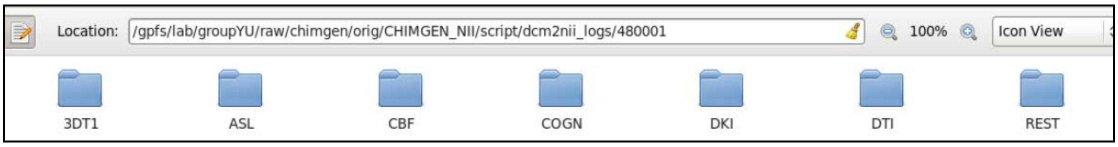

Figure 7.2 Subdirectories of subject 480001

For each participant, the researcher should carefully check the quality and integrity of each type of data. For each research center, when the data collection of all participants is completed, these data should be saved and sent via mobile hard disk drives (HDD) from each center to the TMUGH.

Table 7.2 Standard MRI parameters of T1 weighted structural MRI for different MR scanners

Scanners	Sequences	Matrix	Slices	FOV (mm)	ST (mm)	Resolution (mm)	Gap (mm)	TR (ms)	TE (ms)	TI (ms)	FA (º)	PAT (AF)
GE Discovery MR750	BRAVO Sagittal	256×256	188	256×256	1	1.0×1.0×1.0	0	8.16	3.18	450	12	2
GE Discovery MR750w	BRAVO Sagittal	256×256	188	256×256	1	1.0×1.0×1.0	0	6.93	2.53	450	12	2
GE Signa HDx	BRAVO Sagittal	256×256	188	256×256	1	1.0×1.0×1.0	0	8.85	3.49	450	12	2
GE Signa HDxt	BRAVO Sagittal	256×256	188	256×256	1	1.0×1.0×1.0	0	7.79	2.98	450	12	2
Philips Achieva	TFE Sagittal	256×256	188	256×256	1	1.0×1.0×1.0	0	8.16	3.73	1100	12	2
Philips Ingenia	TFE Sagittal	256×256	188	256×256	1	1.0×1.0×1.0	0	7.27	3.33	900	12	2
Siemens Skyra	MPRAGE Sagittal	256×256	192	256×256	1	1.0×1.0×1.0	0	2000	2.98	900	9	2
Siemens TrioTim	MPRAGE Sagittal	256×256	192	256×256	1	1.0×1.0×1.0	0	2000	2.26	900	12	2
Siemens Verio	MPRAGE Sagittal	256×256	192	256×256	1	1.0×1.0×1.0	0	2000	2.34	900	9	2

Note: AF, acceleration factor; FA, flip angle; FOV, field of view; PAT, parallel acquisition technique; ST, slice thickness; TE, echo time; TI, inversion time; TR, repetition time.

Table 7.3 Standard MRI parameters of resting-state functional MRI for different MR scanners

Scanners	Sequences	Matrix	Slices	FOV (mm)	ST (mm)	Gap (mm)	Resolution (mm)	TR (ms)	TE (ms)	FA (°)	Volumes	PAT (AF)
GE Discovery MR750	GRE-EPI Axial	64×64	36	220×220	3	1	3.4×3.4×4.0	2000	30	90	180	2
GE Discovery MR750w	GRE-EPI Axial	64×64	36	220×220	3	1	3.4×3.4×4.0	2000	30	90	180	2
GE Signa HDx	GRE-EPI Axial	64×64	36	220×220	3	1	3.4×3.4×4.0	2000	30	90	180	2
GE Signa HDxt	GRE-EPI Axial	64×64	36	220×220	3	1	3.4×3.4×4.0	2000	30	90	180	2
Philips Ingenia	GRE-EPI Axial	64×64	36	220×220	3	1	3.4×3.4×4.0	2000	30	90	180	2
Philips Achieva	GRE-EPI Axial	64×64	36	220×220	3	1	3.4×3.4×4.0	2000	30	90	180	2
Siemens Skyra	GRE-EPI Axial	64×64	36	220×220	3	1	3.4×3.4×4.0	2000	30	90	180	2
Siemens TrioTim	GRE-EPI Axial	64×64	36	220×220	3	1	3.4×3.4×4.0	2000	30	90	180	2
Siemens Verio	GRE-EPI Axial	64×64	36	220×220	3	1	3.4×3.4×4.0	2000	30	90	180	2

Note: AF, acceleration factor; FA, flip angle; FOV, field of view; PAT, parallel acquisition technique; ST, slice thickness; TE, echo time; TI, inversion time; TR, repetition time.

Table 7.4 Standard MRI parameters of diffusion tensor imaging for different MR scanners

Scanners	Sequences	Matrix*	Slices	FOV (mm)	ST (mm)	Gap (mm)	Resolution (mm)	TR (ms)	TE (ms)	FA (°)	B=0	B=1000	PAT (AF)
GE Discovery MR750	SE-EPI Axial	128×128	50	256×256	3	0	2.0×2.0×3.0	6000	65	90	5	64	2
GE Discovery MR750w	SE-EPI Axial	128×128	50	256×256	3	0	2.0×2.0×3.0	10000	74	90	5	64	2
GE Signa HDx	SE-EPI Axial	128×128	50	256×256	3	0	2.0×2.0×3.0	12500	72	90	5	64	2
GE Signa HDxt	SE-EPI Axial	128×128	50	256×256	3	0	2.0×2.0×3.0	11500	72	90	5	64	2
Philips Ingenia	SE-EPI Axial	128×128	50	256×256	3	0	2.0×2.0×3.0	7950	111	90	1	32	2
Philips Achieva	SE-EPI Axial	128×128	50	256×256	3	0	2.0×2.0×3.0	6800	91	90	1	32	2
Siemens Skyra	SE-EPI Axial	128×128	50	256×256	3	0	2.0×2.0×3.0	7900	84	90	1	64	2
Siemens TrioTim	SE-EPI Axial	128×128	50	256×256	3	0	2.0×2.0×3.0	6800	91	90	1	64	2
Siemens Verio	SE-EPI Axial	128×128	50	256×256	3	0	2.0×2.0×3.0	6400	98	90	1	64	2

Note: AF, acceleration factor; FA, flip angle; FOV, field of view; PAT, parallel acquisition technique; ST, slice thickness; TE, echo time; TI, inversion time; TR, repetition time.

*For GE scanner, the default Recon matrix is the twice of scan matrix (256 × 256 Recon matrix)

Table 7.5 Standard MRI parameters of DKI for different MR scanners

Scanners	Sequences	Matrix	Slices	FOV (mm)	ST (mm)	Gap (mm)	Resolution (mm)	TR (ms)	TE (ms)	FA (°)	B=0	B=1000	B=2000	PAT(AF)
GE Discovery MR750	SE-EPI Axial	128×128	50	256×256	3	0	2.0×2.0×3.0	6000	71	90	5	25	25	2
GE Discovery MR750w	SE-EPI Axial	128×128	50	256×256	3	0	2.0×2.0×3.0	11000	88	90	5	25	25	2
GE Signa HDx	SE-EPI Axial	128×128	50	256×256	3	0	2.0×2.0×3.0	14000	84	90	5	25	25	2
GE Signa HDxt	SE-EPI Axial	128×128	50	256×256	3	0	2.0×2.0×3.0	14000	84	90	5	25	25	2
Philips Ingenia	SE-EPI Axial	128×128	50	256×256	3	0	2.0×2.0×3.0	13123	89	90	1	20	20	2
Siemens Skyra	SE-EPI Axial	128×128	50	256×256	3	0	2.0×2.0×3.0	7600	90	90	1	20	20	2
Siemens Verio	SE-EPI Axial	128×128	50	256×256	3	0	2.0×2.0×3.0	9600	96	90	1	20	20	2

Note: AF, acceleration factor; DKI, diffusion kurtosis imaging; FA, flip angle; FOV, field of view; PAT, parallel acquisition technique; ST, slice thickness; TE, echo time; TI, inversion time; TR, repetition time.

Table 7.6 Standard MRI parameters of ASL for different MR scanners

Scanners	Sequences	Matrix	Slices	FOV (mm)	ST (mm)	Gap (mm)	Resolution (mm)	TR (ms)	TE (ms)	TI (ms)	FA (º)
GE Discovery MR750	PC-ASL Spiral FSE	128×128	50	240×240	3	0	1.88×1.88×3.00	5046	11.09	2025	111
GE Discovery MR750w	PC-ASL Spiral FSE	128×128	50	240×240	3	0	1.88×1.88×3.00	5070	11.48	2025	111
GE Signa HDxt	PC-ASL Spiral FSE	128×128	50	240×240	3	0	1.88×1.88×3.00	5007	10.44	2025	155

Note: ASL, arterial spin labeling; FOV, field of view; ST, slice thickness; TE, echo time; TI, inversion time; TR, repetition time.
