# Supplementary material for: CHIMGEN: a Chinese imaging genetics cohort to enhance cross-ethnic and cross-geographic brain research
Source: Mol Psychiatry. 2019 Dec 11;25(3):517–29. doi: 10.1038/s41380-019-0627-6 (PMC7042768; doi:10.1038/s41380-019-0627-6)
Supplement: Supplementary file 3 [file 41380_2019_627_MOESM3_ESM.pdf]

QUALITY CONTROL

The CHIMGEN consortium

Content

Chapter 1 Personal information……………………………..………………………2

Chapter 2 Behavioral assessments………………………..………………………4

Chapter 3 Environmental assessments...…….………….…………………..……10

Chapter 4 Blood samples……………………..…….……………………………16

Chapter 5 Genome-wide association study….…….…..…………………………20

Chapter 6 Neuroimaging assessments…….…………..…………………………25

Chapter 1 Personal information

The accuracy of personal information is important for this study. Some personal data may be wrong for various reasons. In this study, several strategies were used to validate the correctness of personal information.

1. Gender

In rare cases, the recorded gender information is incorrect. The gender information of each participant was confirmed in the following processes:

(1) The experimenters checked gender information during the whole process of the experiment, including recruitment, screening, behavioral and environmental assessments, MRI examination and blood sample collection.

(2) The experimenters confirmed the consistency of gender information recorded in different questionnaires.

(3) The experimenters confirmed the participant's gender by examining the genetic sequence of the sex chromosomes.

2. Age

In rare cases, the participant's age is incorrect. The age information was confirmed in the following processes:

(1) The experimenters checked the ID card of each participant to confirm the correctness of his/her age information.

(2) The experimenters checked the consistency of age information recorded in different questionnaires.

3. Ethnicity

Sometimes, the participant's ethnicity is incorrect. The ethnicity information was confirmed in the following processes:

(1) The experimenter confirmed the participant's ethnicity by asking the ethnicities of their parents.

(2) Based on genomic data, the principal component analysis (PCA) was used to capture population structure of the CHIMGEN cohort. This procedure could

identify participants who do not have an ancestry of Chinese Han.

4. Handedness

The handedness of each participant was confirmed by both self-report (in screening and in environmental questionnaire) and the Chinese handedness questionnaire.

5. Smoking

In rare cases, some participants may conceal the information about smoking. This information was confirmed in the following processes:

(1) During recruitment, the experimenter asked each participant whether he/she had smoked more than 20 cigarettes.

(2) In the environmental questionnaire, the participant should answer what brand of cigarettes he/she had ever smoked more than 20.

6. Alcohol drinking

In rare cases, some participants may conceal the information about alcohol drinking. The information was confirmed in the following processes:

(1) The alcohol use disorder identification test (AUDIT) was used to identify participants with excessive alcohol drinking.

(2) In the environmental questionnaire, we confirmed this information by asking the participant *"Have you hurt yourself or someone else because of drinking?"*

7. Medications

In rare cases, some participants may conceal or forget their medication information. The medication information was confirmed in the following processes:

(1) During screening, each participant was asked to provide detailed information of his/her medications.

(2) In the environmental questionnaire, the experimenter confirmed this information by asking the participant two questions about his/her medications:

"Have you ever used sedative-hypnotics or sleep aids in the past one month?"

"Do you have a regular use of drugs or your symptoms will be worse when you stop using?"

Chapter 2 Behavioral assessments

The behavioural data are important components of the CHIMGEN study. Quality control was conducted during the whole process of behavioural assessments. Specific quality control strategies for behavioural assessments included:

1. Quality control before assessments

(1) Tool selection

Selecting reasonable and reliable measurement tools are the prerequisites for obtaining high quality behavioural data. During the experimental design, we selected the behavioural measurement tools with confirmed reliability and validity. Details can be found in the chapter of behavioural and environmental assessments in the standard operating procedures (SOPs).

(2) SOP compiling

The researchers compiled a detailed SOP for each behavioural assessment. In Tianjin Medical University General Hospital (TMUGH), preliminary experiments were performed to identify matters needing extra attention for each behavioural assessment and provided the precautions in the SOP for all centres.

(3) Training workshop

Before the initiation of the study, several training workshops were organized to ensure that experimenters understand the background, concept, purpose, procedure, scoring and precautions of each behavioural assessment. The preliminary experiments showed high consistency of these assessments between experimenters.

(4) Operating system and software

For computer-based assessments, the operating system and software versions were the same between centres to reduce the influence of the non-experimental factors.

2. Quality control during assessments

(1) Environment requirements

Each research centre should prepare at least one evaluation room with a quiet and tidy environment. During behavioural assessment, all unrelated persons should leave the room to reduce disturbances to the participant.

(2) Experimenters

The experimenters should have participated in at least one of our training workshops. Generally, each behavioural assessment was done by 1-2 fixed experimenter(s) in each centre (< 200 participants) to ensure the consistency of the assessment within the centre. In the large centres (> 200 participants), the number of experimenters for each assessment were increased correspondingly.

(3) Instructions for participants

Standard instructions for participants were used in each behavioural assessment. The instructions were explained clearly to the participant to ensure that he or she had fully understood the requirement of each assessment.

(4) Monitoring

The experimenters monitored the attitude of each participant during each behavioural assessment. Participants who were not serious during the assessments were noted and the corresponding data were excluded from the final analyses. The experimenter should answer questions raised by the participant and correct any misunderstandings.

3. Quality control after assessments

3.1 Paper-based assessments

The original records of paper-based assessments should be safely stored in each centre. A PDF version of the behavioural assessments was generated by each centre and then sent to TUMGH. Researchers in TMUGH transformed these original data into a suitable electronic format and calculated the scores of these assessments. Data of each behavioural assessment were processed by a few fixed researchers. The quality control for the process included the following procedures:

(1) Entering electronic data

Two researchers independently transformed the behavioural data of each participant into an editable tabular format. A third researcher checked the data consistency between the two researchers and corrected any inconsistencies.

(2) Excluding unreliable assessments

According to the records of each centre, the behavioural data of participants who were not serious during assessment were excluded. Moreover, items that were not completed as required were excluded too.

(3) Missing values

The researchers should carefully record the missing values of each behavioural assessment in its corresponding electronic table. For each participant, the data of the behavioural assessments with missing values were excluded.

(4) Scoring

The scoring for each behavioural measure was independently completed by two researchers. If the scores were inconsistent between them, they should work together to identify the reason and provide consistent scores.

(5) Unreliable values

For each behavioural assessment, unreliable values were defined as those outside the numeric range of each questionnaire. The behavioural data with unreliable values were excluded.

(6) Data distribution

For each behavioural measure, a histogram was plotted to show data distribution and its normality was tested and recorded.

(7) Consistency testing

Several researchers participated in the time-consuming behavioural scoring. The inter-rater reliability was tested using the intra-class correlation coefficient (ICC). An ICC of 0.75-0.90 indicates good reliability and an ICC of >0.90 indicates excellent reliability. For example, the scoring of the Rey-Osterrieth complex figure test (ROCFT) is operator-dependent. In 20 randomly selected participants, the ROCFT scoring was performed by 19 researchers, and the ICC was estimated using 2-way

mixed-effects model. The ICC values of copy, immediate recall and delayed recall were 0.762 (Figure 2.1), 0.949 (Figure 2.2) and 0.931 (Figure 2.3), respectively. These ICC values were indicative of good or excellent reliability. The relatively low ICC value of copy may be related to the small fluctuation range of values. Therefore, the 19 researchers scored all ROCFTs.

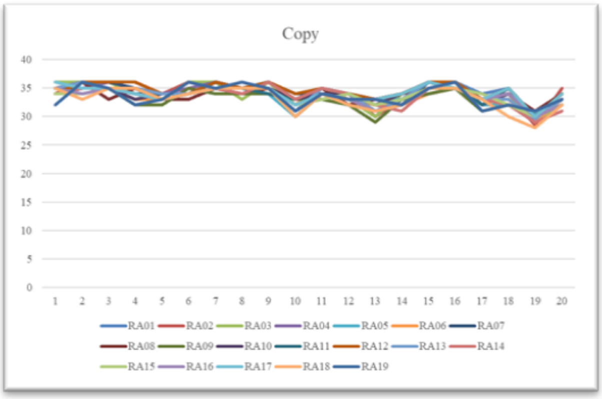

Figure 2.1 Inter-rater reliability for the copy in ROCFT

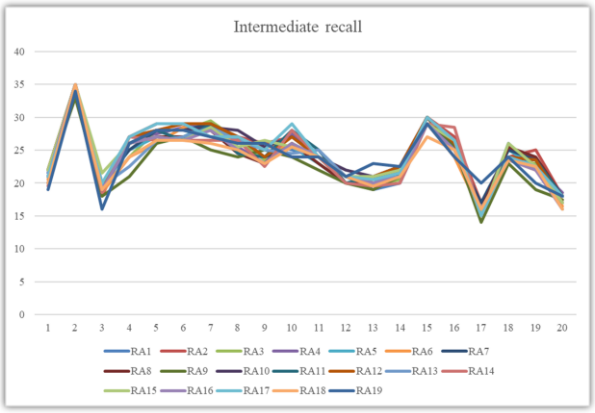

Figure 2.2 Inter-rater reliability for the immediate recall in ROCFT

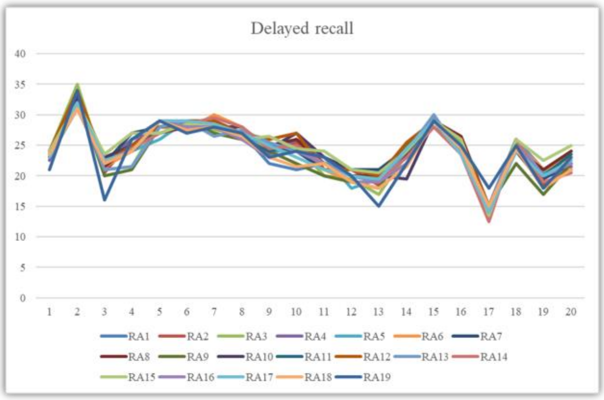

Figure 2.3 Inter-rater reliability for the delayed recall in ROCFT

3.2 Computer-based assessments

The computer-based assessments were stored in electronic formats and need not to be transformed. The quality control included the following procedures:

(1) Excluding unreliable assessments

According to the records of each centre, the data of the computer-based assessments of the participants who were not serious during assessment were excluded.

(2) Missing values

The researchers carefully recorded the missing values of each computer-based assessment in its corresponding electronic table. The assessment data with missing values were excluded.

(3) Data distribution

For each behavioural measure, a histogram was plotted to show data distribution and its normality was tested and recorded.

4. Preliminary results

The first stage of quality control of the CHIMGEN data included 5819 participants and 4,885 of them were genotyped. After excluding 23 participants with metal artefact, 1 with brain atrophy, 1 with excessively large ventricle and 41 with unqualified genetic data, the remaindering 5753 participants (4844 with and 909 without genotyping data) were included in the quality control of behavioural data. The quality control results for behavioural assessments are shown in Figure 2.4.

In the 5753 participants, 5723 (99.48%) had qualified BDI-II, 5722 (99.46%) had qualified STAI, 5728 (99.57%) had qualified TPQ, 5688 (98.87%) had qualified CVLT-II, 5619 (97.67%) had qualified SDMT, 5640 (98.04%) had qualified ROCFT, 5578 (96.96%) had qualified N-back, 5536 (96.23%) had qualified Go/No-Go, 5616 (97.62%) had qualified ball tossing game, and 5639 (98.02%) had qualified UG. Generally, at least 96% participants passed the quality control of each behavioural assessment.

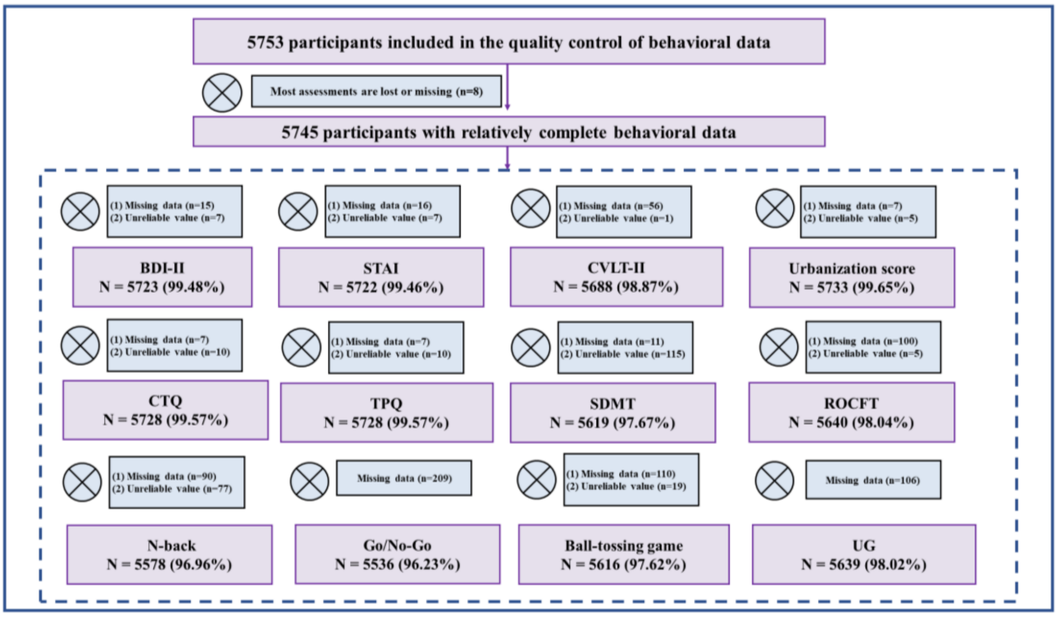

Figure 2.4 Quality control of behavioural assessments

References

Cicchetti, Domenic V. Guidelines, criteria, and rules of thumb for evaluating normed and standardized assessment instruments in psychology. Psychological Assessment 1994; 6(4): 284-290.

Loring D W, Martin R C, Meador K J, et al. Psychometric construction of the Rey-Osterrieth complex figure: Methodological considerations and interrater reliability. Archives of Clinical Neuropsychology 1990; 5(1):1-14.

Koo TK, Li MY. A guideline of selecting and reporting intraclass correlation coefficients for reliability research. Journal of Chiropractic Medicine 2016; 15(2):155-163.

Chapter 3 Environmental assessments

1. Paper-based environmental assessments

The paper-based environmental assessments included a comprehensive environmental questionnaire and a childhood trauma questionnaire (CTQ). The comprehensive environmental questionnaire included the items to compute the urbanization score, the items to validate the childhood trauma questionnaire and the items to validate the personal information. Quality control was conducted during the whole process of the paper-based environmental assessments. Specific quality control strategies included:

1.1 Quality control before assessments

(1) Items selection

During the design of the comprehensive environmental questionnaire, each item was designed for a clear purpose. Items used to calculate the urbanization score have shown high reliability and validity. Items used to validate personal information were designed for those variables that are very important for this study.

(2) SOP compiling

The researchers compiled a detailed SOP for the two environmental assessments. In TMUGH, preliminary experiments were performed to identify matters needing extra attention for each environmental assessment and provided these precautions in the SOP for all centres.

(3) Training workshop

Before the initiation of the study, several training workshops were organized to ensure that experimenters understand the background, concept, purpose, procedure, scoring and precautions of each environmental assessment. Preliminary experiments showed high consistency of these assessments between experimenters.

1.2 Quality control during assessments

(1) Environment requirement

Each research centre should prepare at least one evaluation room with a quiet and tidy environment. During assessment, all unrelated persons should leave the room to reduce disturbances to the participant.

(2) Experimenters

The experimenters should have participated in at least one of our training workshops. Generally, each assessment was done by 1-2 fixed experimenter(s) in each centre (< 200 participants) to ensure the consistency of the assessment within centre. In the large centres (> 200 participants), the number of experimenters for each assessment were increased correspondingly.

(3) Instructions for participants

Standard instructions for participants were used in each environmental assessment. The instructions were explained clearly to the participant to ensure that he or she has fully understood the requirement of each assessment.

(4) Monitoring

The experimenter should monitor the attitude of each participant during each environmental assessment. Participants who were not serious during the assessments were noted and the corresponding data were excluded. The experimenter should answer all questions raised by the participant and correct any misunderstandings.

1.3 Quality control after assessments

The original records of the paper-based environmental assessments should be safely stored in each centre. A PDF version of the paper-based environmental assessments was generated by each centre and then sent to TUMGH. Researchers in TMUGH transformed these original data into a suitable electronic format and calculated the scores of these assessments. Data of each behavioural assessment were processed by a few fixed researchers. The quality control for the process included the following procedures:

(1) Entering electronic data

Two researchers independently transformed the paper-based environmental data of each participant into an editable tabular format. A third researcher checked the data consistency between them and corrected any inconsistencies.

(2) Excluding unreliable assessments

According to the records of each centre, the paper-based environmental data of participants who were not serious during assessment were excluded. Moreover, items that were not completed as required were excluded too.

(3) Missing values

The researchers carefully recorded the missing values of each environmental assessment in its corresponding electronic table. The data of the environmental assessments with missing values were excluded.

(4) Scoring

The scoring for each environmental measure was independently completed by two researchers. If the scores were inconsistent between them, they should work together to identify the reason and provide consistent scores.

(5) Unreliable values

For each environmental assessment, unreliable values were defined as those outside the numeric range of each questionnaire. The environmental assessment data with unreliable values were excluded.

(6) Data distribution

For each environmental measure, a histogram was plotted to show data distribution and its normality was tested and recorded.

1.4 Preliminary results

The same as the quality control of behavioural data, 5753 participants (4844 with and 909 without genotyping data) were included in the quality control of the paper-based environmental data. The quality control results are shown in Figure 2.4. In the 5753 participants, 5733 (99.65%) had qualified urbanization data and 5728 (99.57%) had qualified CTQ data.

2. Lifelong geographic information

Collecting lifelong residential locations of each participant is critically important for calculating longitudinal environmental measures based on remote sensing satellite images and national survey databases. The following strategies were used to ensure the accuracy of the geographic information:

(1) Information integrity

In addition to the home address of each participant, we also recorded the school or work address of the participant to truly reflect the impact of the environmental factors on the participant. Specifically, the living address during the daytime was taken as the main address and the address at night as the secondary address. In the statistics, we can analyze these data separately or integrate these two datasets into a comprehensive measure with different weights.

(2) Precision

Besides providing the address information, the participant provided the precise latitude and longitude coordinates of the annual residential address by searching and marking the position on an electronic map.

(3) Validation

Researchers should use the address information in the comprehensive environmental questionnaire to validate the coordinate information provided by the participant.

3. Remote sensing data

A total of 847 datasets were obtained from the three databases (382 from Google Earth Engine, 244 from Resource Watch, 221 from Socioeconomic Data and Applications Centre). After excluding datasets without China data, with low spatial resolution or without data after 1985, we acquired 552 remote sensing datasets, including 293 datasets from the GEE database, 140 from the Resource Watch database, and 119 from the Socioeconomic Data and Applications Centre database (Figure 3.1).

It should be noted that the obtained 552 remote sensing datasets were derived from the prescreening and thus further quality control should be applied to screen qualified remote sensing measures for a specific research purpose. For example, if we want to identify the sensitive periods during which environmental factors show the most

significant influence on the brain, satellite measures should meet the following requirements:

(1) The satellite measures should be available. Only satellite measures that are freely available were selected.

(2) The satellite measures should be reliable. Only satellite measures that have been validated in previous studies were selected.

(3) The satellite measures should have sufficient temporal resolution (< 1 year).

(4) The satellite measures should have sufficient spatial resolution (less than 10 km).

(5) The satellite measures should have sufficient temporal coverage. These measures should cover the lifetimes of most participants. For example, as most participants of the CHIMGEN study were born after 1990, only satellite measures that are available for most years after 1990 are suitable for the study purpose. Those measures that are available only for a few years of data were excluded.

(6) The satellite measures should have sufficient spatial coverage (including the mainland of China).

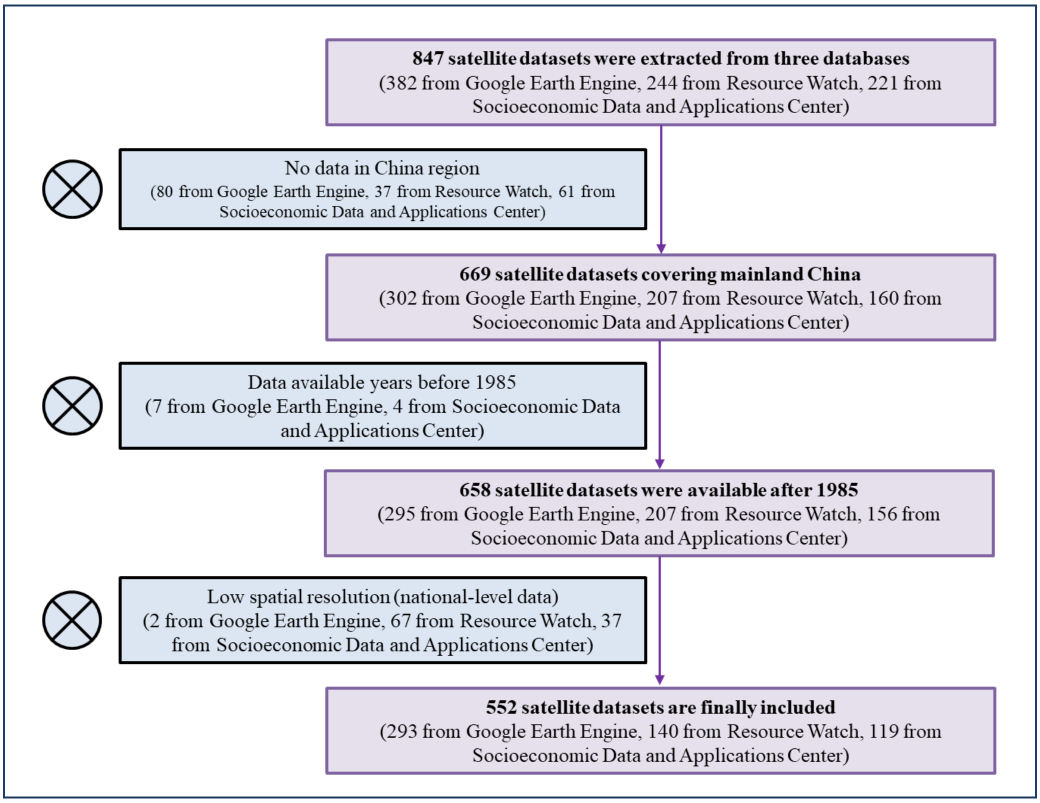

Figure 3.1 Preliminary quality control of satellite datasets

4. National survey databases

Based on the annual residential coordinates of each participant since birth, many lifelong socioeconomic measures of the participant were obtained from national survey databases. Similar to the remote sensing measures, the quality control procedures were also dependent on the specific research purposes. Also taking the same scientific question (i.e., sensitive periods during which environmental factors show the most significant influence on the brain) as an example, socioeconomic measures should satisfy the following requirements:

(1) The measures should be freely available.

(2) The measures should be reliable and the data should be correct.

(3) The measures should have sufficient temporal resolution (1 years).

(4) The measures should have sufficient spatial resolution (at least at the level of province).

(5) The measures should have sufficient temporal coverage. These measures should cover the lifetime of most participants.

5. Cross validation

Overlapping information may exist for environmental assessments in the CHIMGEN study. For example, the urbanization could be reflected by the urbanization score derived from the comprehensive environmental questionnaire, the global human settlement layer derived from the remote sensing database, and the population density derived from the national survey database. In such cases, the correlation coefficients of these measures from different sources should be greater than 0.5.

Chapter 4 Blood samples

1. Blood sample collection for DNA examination

Blood samples for DNA examinations were collected using the EDTA tubes. The quality control procedures for blood sample collection were as follows:

1.1 Quality control before collection

The blood sample collection is performed after behavioral assessments and MRI examinations. To ensure the correspondence between different types of data for the same participant, the experimenter should carefully label the EDTA tubes with the collection date, participant's ID and name on both the tube body and lid.

1.2 Quality control during collection

(1) Identification check

Before venous puncture, the nurse on duty should confirm the consistency between the real and the labelled information of the participant.

(2) Venous puncture

Venous puncture should be performed by experienced nurses. If the first puncture was failed, a second puncture was performed after obtaining the permission from the participant.

(3) Sterile operation

The nurse on duty should perform sterile operation thoroughly to avoid pollution of blood samples.

(4) Hemolysis prevention

To prevent hemolysis, several strategies are applied:

(a) The nurse should select appropriate needle size;

(b) The nurse should perform a high quality venous puncture;

(c) The participant should avoid excessive fist clenching;

(d) A slow blood flow should be maintained;

(e) The tube should be maintained in an appropriate temperature;

(f) Violent vibration was strictly prohibited.

(5) Checking the quality of the blood sample

The experimenter should carefully check the quality of the blood sample by observing the color of the sample: transparent yellow indicates bilirubin; milky white and turbid indicates chylomicron; clear red indicates hemolysis; blood clot indicates coagulation. In these cases, the blood samples are not qualified and should be re-collected.

1.3 Quality control after collection

(1) Intervals between collection and centrifugation

The collected blood samples should be centrifuged within 2 hours stored at room temperature (20-25°C), and within 4-6 hours stored in a 4 °C refrigerator.

(2) Storage

After centrifugation, the blood samples should be placed in an ultra-low temperature freezer at -80 °C in time.

(3) Transportation

Blood samples should be shipped with sufficient dry ice to avoid samples from thawing during transportation.

(4) ID check

The identification of samples was checked during the whole process. This is especially important when simultaneously processing multiple blood samples.

2. Blood collection for RNA examination

Blood samples for RNA examinations were collected using the PAXgene® blood RNA tubes. The quality control procedures for blood collection were as follows:

2.1 Quality control before collection

(1) PAXgene® blood RNA tubes cannot be re-used.

(2) Expired PAXgene® blood RNA tubes cannot be used.

(3) Unused PAXgene® blood RNA tubes should be stored at 18-25℃.

(4) All tubes should be clearly labeled with the sample collection date and the participant's ID.

2.2 Quality control during collection

(1) The RNA tube should be the last one to be used when multiple tubes of blood

samples need to be collected.

(2) If the RNA tube was the only tube to be used, the nurse should draw the first 1-2ml blood sample into another tube and then draw blood sample into the PAXgene® blood RNA tube.

(3) The following techniques should be used to prevent backflow:

(a) The participant's arm was placed in a downward position.

(b) The RNA tube should be maintained in a vertical position below the arm during blood collection.

(c) The tourniquet should be released as soon as the blood starts to flow into the tube.

(d) The tube additives cannot touch the end of the needle.

(3) Blood sample cannot be transferred from a syringe to the RNA tube.

(4) The nurse should perform sterile operation thoroughly to avoid pollution of blood samples.

2.3 Quality control after collection

(1) The PAXgene® blood RNA tubes should be placed upright in an iron rack instead of foamed plastic rack to avoid cracking of RNA tubes.

(2) The PAXgene® blood RNA tubes should be kept at room temperature (18-25°C) for 2-72 hours before transferring to freezer at -20°C for 24 hours.

(3) The PAXgene® blood RNA tubes should be long-term stored in freezers at -80°C

3. Blood sample transportation

After sample collection, the blood samples were transported to the TMUGH by a professional biomedical cold chain logistics company. Several strategies were applied to ensure the safety and quality of blood samples (details are provided in the SOP).

(1) A professional biomedical cold chain logistics company was in charge of the transportation of blood samples.

(2) We had designed a detailed pipeline for the transportation.

(3) The temperatures of samples were monitored during the whole process.

(4) We had established a team to deal with any accidental problems.

(5) Regular quality assessments were used to eliminate potential safety hazards.

3. DNA extraction

(1) The experimenter should carefully clean and calibrate the Nanodrop2000.

(2) The DNA concentration and purity should be recorded.

(3) The EP tube containing the extracted DNA should be marked with the date, participant's ID and name.

(4) The extracted DNA tube should be sent back to the biobank in time, and must be recorded with the following information: the center name, quantity, return time and personnel signature. If the DNA extraction is failed, this should be recorded and the person in charge should be informed in time.

(5) The identification of the participant should be checked and remain consistent during the whole process.

Chapter 5 Genome-wide association studies

1. Overview

Genotyping with the Illumina Infinium Asian Screening Array (ASA) generates a dataset for each qualified participant. Because of the high homogeneity of racial identity and a proper sample size of the CHIMGEN cohort, we conducted the quality control for the genome-wide association studies (GWAS) in a typical way as in literature. Briefly, genetic quality control was performed both in variant-level and sample-level. In variant-level quality control, we excluded duplicated markers and markers with high allele missing rate or departure from Hardy-Weinberg equilibrium (HWE). In sample-level quality control, we identified and excluded samples with sex mismatching, poor quality samples determined by the combination of heterogeneity and missing rate, related samples with lower call rate, and samples deviating from the population structure indicated by principal component analysis (PCA).

2. Genotyping

The Illumina Infinium ASA was used to identify ~750,000 (~650,000 ASA sites plus ~100,000 customized sites) genetic variants for each participant. In addition to the routine quality control schemes, we also included the following procedures:

(1) Array selection

The ASA was selected for genotyping because it is specially designed for East Asian populations, which is well matched with the CHIMGEN population.

(2) Batches

In the first stage of the CHIMGEN study, 7,000 participants are collected and will be genotyped in two batches (5,000 and 2,000). This strategy would reduce variations from the batch effects.

(3) Blind test

The blind test was designed to ensure the quality of genotyping. The blind test repetition rate should be greater than 99%. If the detection rate was less than 97%, the corresponding sample needed to be genotyped again. Additionally, samples from the

ASA chip that contained unqualified sample should be re-genotyped again to ensure the call rate.

3. Variant-level quality control

The removal of poor quality markers is critical to the success of GWAS because they may be false positive markers and reduce the power to identify true associations with the interested phenotypes. The specific procedures for quality control were as follows:

3.1 Deleting duplicated markers

It is a routine to design some duplicated markers in array to ensure correct genotyping for significant markers and for markers located in regions enriched with AT or GC. There are 16,601 duplicated markers in ASA. We retained one marker for each locus by excluding the duplicated one with higher genotype missing rate among samples.

3.2 Deleting markers with high missing rate

Conventionally, markers with call rates less than 95% are removed from the further analysis. We used the same threshold as in most literatures.

3.3 Deletion of markers deviating from HWE

In GWAS, markers with significant deviation from Hardy-Weinberg equilibrium (HWE) should be deleted because it is an indication of a genotype-calling error or population stratification. The statistical threshold for declaring SNPs deviating from HWE in this study was set to 10^{-5}.

4. Sample-level quality control

The sample-level quality control of the CHIMGEN genetic data consisted of four steps to identify samples with sex mismatching, to find samples with poor genotyping quality, to deal with related samples, and to provide the ancestral background for samples based on genetic information.

4.1 Identification of samples with sex mismatching

We used genotype data from the X chromosome to check for discordance in sex information. The mismatch between inferred and reported sex may be caused by sample mix-up, transgender individuals, or DNA contamination. Theoretically, males

have one copy of X chromosome and should be homozygous for any marker outside the pseudo-autosomal region in the X chromosome. In this study, male samples are expected to have a homozygosity rate of more than 0.8, and female samples are expected to have a homozygosity rate of less than 0.2. Samples with sex mismatching were excluded from further analyses.

4.2 Identification of duplicated and related samples

A basic feature of the standard population-based association studies is that all samples are unrelated. If duplicates and relatives are present, a bias may be introduced in the study because the genotypes within one family will be overrepresented. Although we tried our best to exclude duplicated and related participants during the recruitment, such participants might still exist in the cohort. To identify the duplicated and related individuals, we calculated the identity by descent (IBD) for each pair of individuals. The extended linkage disequilibrium (LD) regions were removed entirely to obtain independent SNPs. The remaining regions were pruned so that no pair of SNPs within a given window (100 markers) was correlated ($r^2 < 0.2$). Any pair of individuals with an IBD value more than 0.1875 was considered as duplicated or related individuals. In this situation, the one with higher call rate was reserved.

4.3 Identification of outliers of genotype missing and heterozygosity

The extreme heterozygosity and high missing rate are indicators of poor sample quality due to DNA contamination or other causes. The heterozygosity was defined by (N-O)/N, where N was the number of non-missing genotypes and O was the observed number of homozygous genotypes for a given sample. Individuals were excluded if the genotype failure rate was greater than 0.03 or the heterozygosity rate was outside the range of ± 3 standard deviations of the mean.

4.4 Identification of samples with divergent ancestry

Population stratification is a major source of bias in population-based GWAS, in which significant associations are generated because of different population origins rather than any genetic effect on the interested phenotypes. Principal component analysis (PCA) was used to capture population structure within the CHIMGEN cohort.

The participants with significant deviation from population were excluded and the top 20 components would be regressed out in the GWAS.

5. Preliminary results

In the first stage, 4893 participants have qualified blood samples. After excluding 7 participants with metal artefact and 1 participant with excessively large ventricle, the remaining 4885 participants were genotyped with the ASA. The quality control results for genetic data are shown in Figure 5.1 and Figure 5.2. In the 4885 participants, 4844 participants (99.16%) had qualified genetic data. PCA showed only one participant obviously deviating from the population.

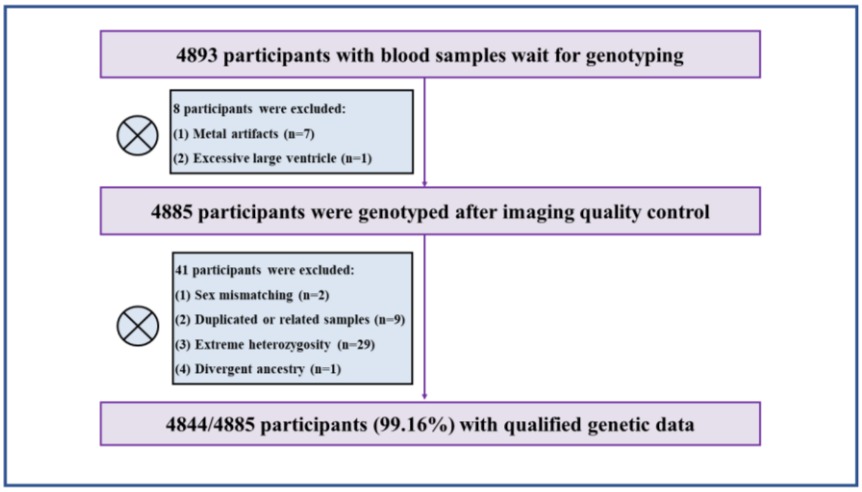

Figure 5.1 Preliminary results of quality control for genetic data

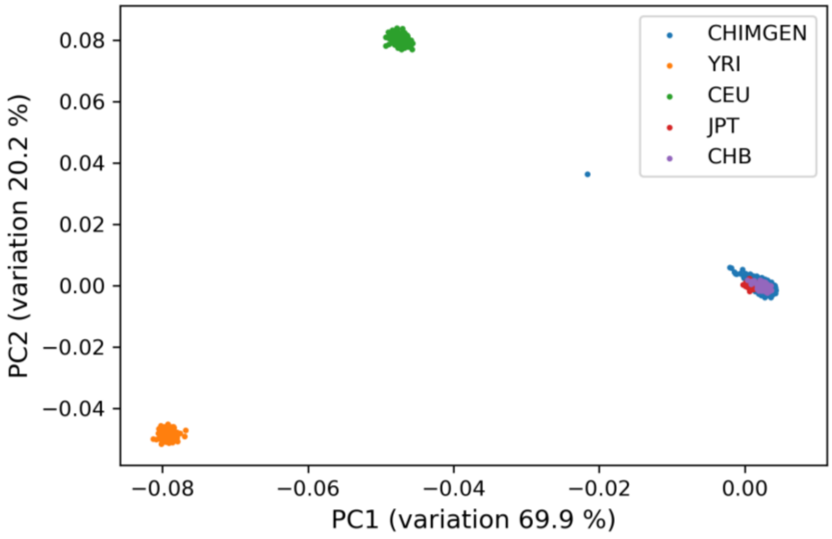

Figure 5.2 Principal component analyses for identifying population stratification.

5. Imputation

In this study, we firstly performed pre-phasing using SHAPEIT2 software in chunks of 5,000 kb with an overlap of 250 kb between chunks. Imputation was conducted with the IMPUTE2 with the merged database of 1000 Genomes project phase 3 and hundreds of sequenced Chinese Han individuals as the reference panel. Here, the use of hundreds of sequenced Chinese Han individuals was to increase the accuracy of imputation.

References

Anderson CA, Pettersson FH, Clarke GM, et al. Data quality control in genetic case-control association studies. Nature protocols 2010; 5(9): 1564-1573.

SHAPEIT2: https://mathgen.stats.ox.ac.uk/genetics_software/shapeit/shapeit.html

IMPUTE2: http://mathgen.stats.ox.ac.uk/impute/impute_v2.html

Chapter 6 Neuroimaging assessments

The brain MRI data are the core data of the CHIMGEN study. Since different MRI scanners were used in this study, the quality control procedures for neuroimaging data are critical for generating reliable results. In this study, the quality control was performed throughout the whole process, including quality control procedures before scanning, during scanning, immediately after scanning, before preprocessing, during preprocessing and during analyses.

1. Quality control before scanning

(1) The selection of MRI scanners

Although the inclusion of all types of scanners could increase the number of centers and the feasibility of this study, this may introduce scanner-related differences in neuroimaging measures. Based on the reliability and availability of MRI scanners, only 3.0 Tesla MRI scanners from GE, Siemens and Philips were included in this study to ensure high image quality. To further reduce the influence of MRI scanners from different manufactures, we encouraged centers to acquire data using the MR 750 scanner from GE for its relatively high image quality and popularity in China.

(2) The selection of sequences

Theoretically, more MRI sequences could provide more information about our brains. However, due to the limit of the endurance of participants, only limited number of sequences can be selected. All centers are required to collect T1-weighted structural MRI (sMRI), diffusion tensor imaging (DTI) and resting-state functional MRI (rs-fMRI) data, and are encouraged to also collect diffusion kurtosis imaging (DKI) and arterial spin labeling (ASL) data if possible.

(3) Imaging parameters for each sequence

Based on our experiences and references, we firstly designed a set of parameters for the GE MR 750 scanner. The imaging quality derived from these parameters has been validated in our previous studies. According to the critical parameters of the MR 750 scanner, the experienced technicians from TMUGH proposed schemes of parameters

for other types of scanners. The researchers of each individual center acquired images from three volunteers using the proposed parameters and sent these images to the TMUGH for quality check. The quality check was done slice by slice and a report on imaging quality was provided to each center. If the images failed the quality check, a proposal of modifications of imaging parameters was also provided to the center to improve imaging quality. This procedure was repeated until each center obtained high-quality neuroimaging data. Detailed methods for quality assessments are provided in the following section “quality control of neuroimaging data before preprocessing”. The finalized scan parameters were stored in a safe place so that these parameters could be easily restored if needed. These optimized scan parameters should be used to acquire MRI data for all participants and should not be changed during the study.

(4) Training workshops

Several training workshops were held before MR data collection. In these workshops, several experienced technicians explained the meaning of each MRI parameter, how to set parameters for each scan sequence as required, how to perform a qualified MRI examination, how to examine the quality of neuroimaging data, and precautions for MRI examinations.

2. Quality control during scanning

(1) Scanner quality control

To ensure the scanner was in a good state, each center was strongly recommended to perform regular quality assessments using phantoms. If problems were identified, the MRI data collection had to stop until the problems were resolved. It should be noted that 27 MR scanners were used to acquire MRI data for the 29 centers because the MRI data of the center 11 were acquired in the center 1 and the MRI data of the center 15 were acquired in the center 2.

For each MR scanner, two phantoms were used to assess the imaging quality of the scanner. Specifically, an American College of Radiology (ACR) MRI phantom was used to assess the functioning of MR scanner, including geometric distortion,

slice positioning and thickness accuracy, high contrast spatial resolution, intensity uniformity, ghosting artefacts and low contrast object detectability, which were done in required slices of the ACR images acquired by ACR T1 and T2 sequences. Detailed methods and criteria please see documentations of the phantom test guidance (https://www.acraccreditation.org/-/media/ACRAccreditation/Documents/MRI/Large PhantomGuidance.pdf?la=en).

A custom phantom was used to evaluate temporal stability during functional MRI data acquisition. We performed all the quality control analyses based on a series of 200 volumes from the middle slice through the phantom. Detailed information please see documentation of Function Biomedical Informatics Research Network (FBIRN) Stability phantom quality assurance procedures (https://www.nitrc.org/frs/download.php/275/fBIRN_phantom_qaProcedures.pdf).

Two healthy volunteers were scanned at all centers to assess the consistency of MRI data acquired by different MR scanners. Voxel-wise spatial correlation matrices are used to assess the consistency of common MRI measures calculated from MRI data of the two subjects acquired by the 27 MR scanners. The effects of scanners on the GMV, ReHo and FA are shown in Figure 6.1. These measures showed high consistency for MRI data acquired by the same type of MR scanner with the same scan parameters; however, there were visible differences for MRI data acquired by different types of MR scanners.

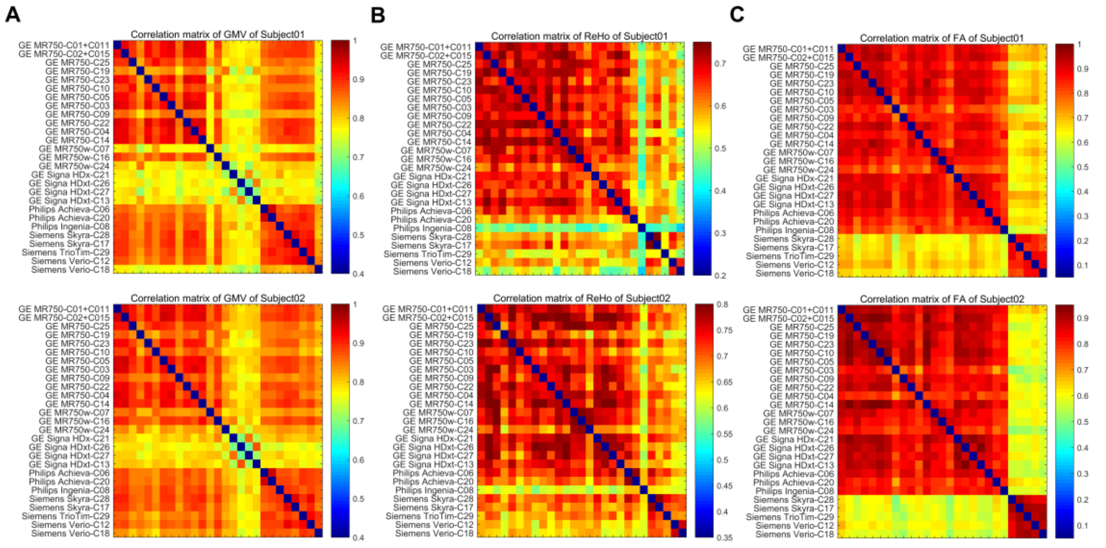

Figure 6.1. Voxel-wise spatial correlation matrices of common MRI measures calculated from MRI data of two human volunteers acquired by different MR scanners (n = 27).

Voxel-wise spatial correlation matrices are used to assess the consistency of grey matter volume (GMV) (A), regional homogeneity (ReHo) (B), and fractional anisotropy (FA) (C) calculated from MRI data of subject 01 (upper row) and subject 02 (lower row) acquired by the 27 MR scanners. X and Y axes indicate the type of MR scanner used in each center. Color bar denotes the correlation coefficient.

(2) Checking the scan parameters

Before each MRI examination, the researcher should check whether the scan parameters have been correctly set. This is important because most scanners are also used for clinical scans using parameters different from the ones designed for this study.

(3) Confirming the identification of the participant

The experimenter should confirm the identification of the participant.

(4) Checking the safety information

The researcher should check the safety information item by item before the participant enters the MRI room.

(5) Preventing participants from head motion

The experimenter should explain the importance of remaining a motion-free state for

a successful MRI examination and ask the participant to maintain a motion-free state during the MRI examination. Moreover, the tight but comfortable foam padding was used to minimize head movement. When the participant cannot endure the whole process of the examination, the researcher can let the participant take a rest at the interval between two sequences.

(6) Reducing the effect of scanner noise

The participant should wear magnetic-compatible earplug or earphone to reduce the effect of scanner noise.

(7) Instructions for participants

Standard instructions for MRI examinations should be given to all participants. For example, the standard instruction for the resting-state fMRI examination is: “Please keep your eyes closed, stay as still as possible, think of nothing in particular, and please do not fall asleep”. A short questionnaire or oral communication should be performed immediately after the examination to ensure that the participant has done as required during the examination. If the participant failed to follow the instruction, the rs-fMRI data should be re-acquired.

(8) Identification of brain abnormalities

The experimenter should check the T2-weighted images (T2WI) and sMRI data to identify potential brain lesions and structural abnormalities. If brain lesions or abnormalities were identified, the experimenter should inform the participant and the participant should be excluded from this study.

(9) Checking imaging quality

The experimenter should check the image quality of each sequence to identify observable artifacts on each imaging sequence. The images with artifacts should be re-acquired.

(10) Checking imaging parameters

In rare situations, imaging parameters, such as the field of view, slice thickness and number of slices, might be changed by accident. The experimenter should check the DICOM header for each imaging modality after acquisition to ensure all imaging

parameters are consistent with the pre-defined settings. The unqualified images should be re-acquired.

(11) Checking head motion

The head motion of the rs-fMRI data should be checked by the realign function of SPM version 12 (https://www.fil.ion.ucl.ac.uk/spm/software/spm12/). If the maximum displacement in any of the three orthogonal directions is more than 3 mm or a maximum rotation is greater than 3.0°, the rs-fMRI data should be re-acquired.

3. Quality control immediately after scanning

(1) Copying data from scanner to disk

The experimenter should copy neuroimaging DICOM data of each participant from the scanner to the mobile disk, and allocate each type of data into an independent sub-folder (Figure 6.2).

(2) Confirming the participant's ID for the neuroimaging data

The experimenter should confirm the participant's ID for the neuroimaging data to avoid allocating neuroimaging data to the wrong person.

(3) Checking the number of images

In rare situations, a few slices might get lost during data copying. The experimenter should check the number of slices for each sequence to ensure the complete data being copied.

(4) Data storage

The neuroimaging data should be stored in at least two independent disks to ensure the data safety. It is strongly recommended that all data should be stored in two

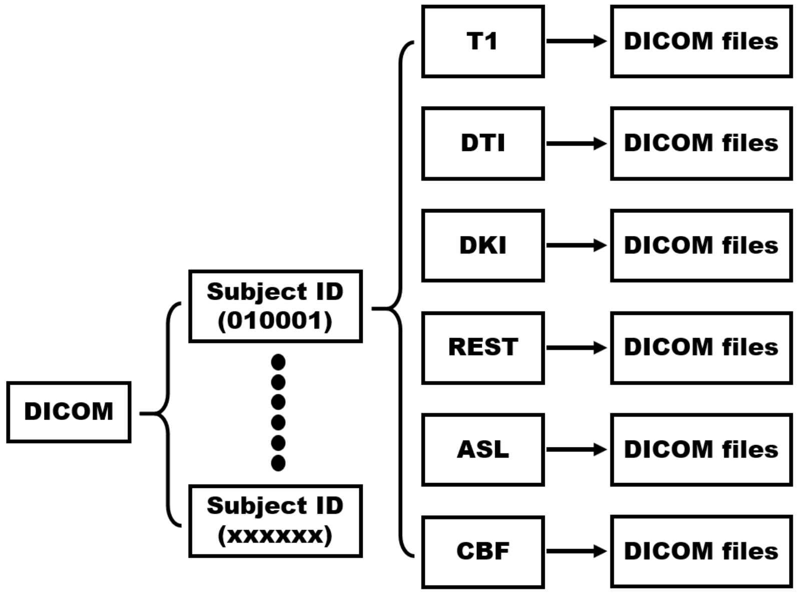

centers located in different cities.

Figure 6.2 Folder structures of DICOM data

The first 2 numbers of the ID encode the center and the last 4 numbers represent the subject identity encoded in order in that center

4. Quality control before preprocessing

4.1 DICOM data sorting

The DICOM data of each participant were allocated into different folders according to the data type before batch preprocessing. When the neuroimaging data are sent to the TMUGH, data files are organized according to the following procedure:

(1) Confirming the correspondence between participants' ID and neuroimaging data

In rare cases, the experimenters might make mistakes in inputting the ID number during MRI acquisition or data allocation. The researchers from the quality control group for neuroimaging data should check the consistency of the participant's ID between the DICOM header file and the folder name. If inconsistent, the data should be returned to the center where it was collected for correction:

(a) If the participant's ID number recorded in the DICOM header file was confirmed to be incorrect, the wrong ID number in the DICOM header file was corrected.

(b) If the participant's ID number recorded in the folder name was confirmed to be incorrect, the neuroimaging data will be re-allocated to the correct subject's folder.

(c) If the reason for the inconsistency cannot be determined, the neuroimaging data of both participants indicated by the DICOM ID and the folder ID will be excluded from the study.

(2) Confirming the consistency between imaging modality and sub-folder

The researchers should check whether neuroimaging data of each imaging modality are allocated to the correct sub-folder, and corrections would be made if any mistakes were identified.

(3) Checking the folder names

All folders should be named according to the pre-defined naming scheme (Figure 6.2). If inconsistency was identified, corrections would be made for the identified folder names.

(4) Checking the completeness of images

In rare situations, a few DICOM images might get lost during copying and transferring. The experimenter should check the number of DICOM images for each imaging modality before DICOM-to-NIFTI transformation. If the number of images was less than the required number for an imaging modality, this imaging modality would be excluded from this study.

4.2 DICOM-to-NIFTI transformation

We have developed a batch program to transform DICOM to NIFTI format based on the dicm2nii software version 2018.08.08 (https://github.com/xiangruili/dicm2nii). This program supports multi-cluster parallel processing and standard output formatting. The standard output format is shown in Figure 6.3.

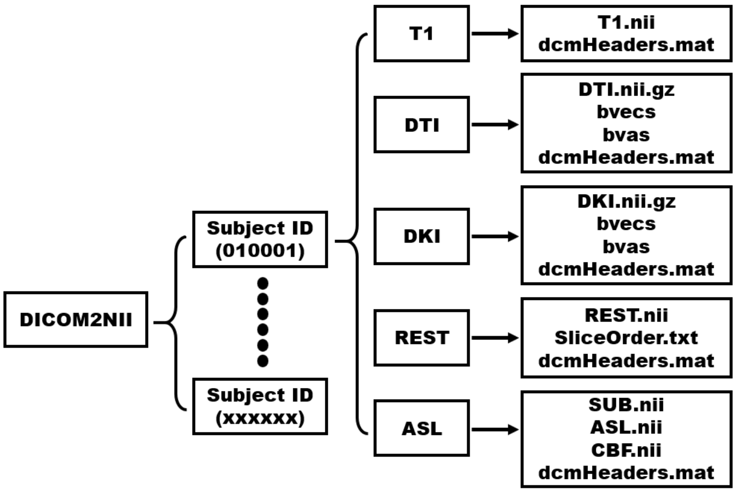

Figure 6.3 Standard output format after DICOM-to-NIFTI transformation.

4.3 Image quality assessments

4.3.1 Brain abnormalities

The experienced radiologists should check the T2WI and structural images to identify possible brain lesions and other abnormalities. If brain abnormalities were confirmed,

the participant should be excluded from this study.

4.3.2 Imaging quality

Imaging quality was evaluated either automatically using scripts or manually, or both, as follows.

(1) Parameter inconsistency

The imaging parameters of each sequence were checked center-by-center based on the information in the dcmHearders.mat file. If the parameters in the header were different with the required ones, the corresponding imaging data were excluded.

(2) Incomplete coverage

If the spatial range of imaging did not cover the entire brain tissue, the corresponding neuroimaging data were excluded from this study. The presence of the aliasing artifact in slices with brain tissue was also considered as incomplete coverage and the corresponding data would be excluded.

(3) Metal artifacts

Although participants with exogenous and endogenous metal materials are prohibited from MRI examinations and metal artifacts are visually checked during the MRI scanning, a few participants still have metal artifacts due to various reasons, such as the fixed denture and collar. The researchers in TMUGH manually checked the transformed imaging data again. If metal artifact was identified in any image, all imaging data of this participant were excluded from the study.

(4) Systematic artifacts

In rare cases, several types of systematic artifacts, such as the zipper noise, spike and RF overflow artifacts, can be seen in the MR images. The researchers should manually check all images and exclude those data with any systematic artifacts from the study.

(5) Head motion artifacts

Head motion artifacts have different manifestation patterns according to the imaging modality, such as the ghost artifact in sMRI and ASL images and the displacement and signal dropout in DTI, DKI and rsfMRI images. The researchers should visually

check the ghost artifacts for the sMRI and ASL data of each participant, and exclude the imaging data with ghost artifacts. For the DTI and DKI data, an “eddy” toolbox implemented in the FSL 5.0.10 (https://fsl.fmrib.ox.ac.uk/fsl/fslwiki/eddy) was used to evaluate and correct image displacement and signal dropout caused by head motion. This toolbox also corrected the eddy-current induced image distortion in these data. The software of the SPM12 (https://www.fil.ion.ucl.ac.uk/spm/software/spm12/) was used to evaluate and correct head motion for the rs-fMRI data. If the maximum displacement in any of the three orthogonal directions was more than 3 mm or a maximum rotation was greater than 3.0°, the rs-fMRI data of this participant should be excluded.

5. Quality control during data preprocessing

We developed a CHIMGEN pipeline to preprocess the multi-model neuroimaging data. The pipeline integrated many popular neuroimaging tools:

(1) dicm2nii (https://github.com/xiangruili/dicm2nii)

(2) FreeSurfer (http://www.freesurfer.net/fswiki)

(3) SPM (https://www.fil.ion.ucl.ac.uk/spm/software/spm12/)

(4) FSL (https://fsl.fmrib.ox.ac.uk/fsl/fslwiki/)

(5) CAT12 (http://www.neuro.uni-jena.de/cat/)

(6) Diffusion toolkit/TrackVis (http://www.trackvis.org)

(7) DKI (https://github.com/NYU-DiffusionMRI/Diffusion-Kurtosis-Imaging)

The integrated pipeline supports multi-cluster parallel computation, and the total computation time can be greatly reduced by using the Tianhe-1 supper-computer (https://www.nscc-tj.cn/). At last, a standardized automatic workflow was developed for each type of the neuroimaging data to increase preprocessing consistency across participants. The specific preprocessing procedures for each type of neuroimaging data were shown as follows:

5.1 Structural MRI data

5.1.1 Voxel-based morphometry (VBM)

The CAT12 software (version r1364, http://dbm.neuro.uni-jena.de/cat) was used for

the VBM analyses with a standard configuration.

(1) Bias correction

Image inhomogeneity caused by B1-field bias was corrected to improve the accuracy of the segmentation of the brain tissues. The bias corrected images had more uniform intensities within each type of the brain tissues.

(2) Segmentation

The bias-corrected structural images were reliably segmented into gray matter (GM), white matter (WM) and cerebrospinal fluid (CSF). The segmentation model was based on an adaptive Maximum A Posterior (MAP) technique without the need for a priori information about tissue probabilities.

(3) Creating population-specific tissue templates

To improve the quality of registration, population-specific tissue probability templates in Montreal Neurological Institute (MNI) space were derived from 5743 CHIMGEN participants by the DARTEL toolbox implemented in SPM12.

(4) Spatial normalization

The segmented images were spatially normalized to the population-specific templates using a two-step DARTEL algorithm and resampled into a resolution of cubic voxels of 1.5×1.5×1.5 mm^3. Modulation was performed on the normalized GM images to preserve the absolute volume of the GM. For detailed information, please refer to the CAT12 website: http://dbm.neuro.uni-jena.de/cat/index.html#VBM.

(5) Smoothing

The resultant images were spatially smoothed with a full width at half maximum (FWHM) Gaussian kernel of 8 mm to compensate for residual anatomical differences between participants.

5.1.2 Surface-based morphometry (SBM)

The FreeSurfer v6.0.0 (http://surfer.nmr.mgh.harvard.edu/) was used for the SBM analyses with the following steps:

(1) Skull stripping

The automated skull-stripping was performed to separate the brain from non-brain

tissues for structural MR images.

(2) Intensity normalization

Intensity normalization was used to correct signal non-uniformity due to variations in the sensitivity of the reception coil and gradient-driven eddy currents.

(3) Tissue segmentation

A series of tissue segmentation procedures were performed on the skull-stripped and intensity-normalized images based on intensity and neighbor constraints to generate the boundary between GM and WM.

(4) Surface reconstruction

A two-dimensional tessellated mesh was constructed based on the boundary between WM and GM to generate the WM surface in each hemisphere, and the WM surface was extended outwards by tracking the gray matter intensity gradient to generate the pial surface. Topology correction was performed to repair topological defects.

(5) Metric reconstruction

Surface-based metrics such as the cortical thickness, surface area, cortical volume and cortical curvature were calculated based on the pial and WM surfaces.

(6) Spherical normalization

Individual surfaces were inflated into a spherical space and registered to a spherical atlas in MNI152 space (the fsaverage atlas). Surface-based metrics for each cortical area were extracted based on the predefined surface atlas after registration to individual spaces using the spherical registration parameters.

(7) Smoothing

The surface-based metrics were smoothed with a Gaussian kernel of 20 mm FWHM.

5.2 DTI

(1) Brain extraction (BET)

The non-brain tissues of the b = 0 images were removed by the brain extraction tool (BET) implemented in FSL.

(2) Motion and distortion correction (EDDY)

An “EDDY_OPENMP” program implemented in the FSL 5.0.10 was used to evaluate

and repair image displacement and signal dropout caused by head motion, and image distortion caused by eddy current.

(3) Tensor metric calculation (DTIFIT)

The linear least square algorithm implemented in the DTIFIT program was used to estimate the diffusion tensor and to calculate diffusion metrics, including the three eigenvalues, fractional anisotropy (FA), and mean diffusivity (MD).

(4) Spatial normalization estimation (BBR+DARTEL)

A two-step procedure was used to estimate the co-registration parameters between individual diffusion space and MNI standard space.

(a) Individual b = 0 images were aligned to the corresponding structural images using the Boundary-Based Registration (BBR) algorithm implemented in FSL.

(b) The BBR parameters were concatenated with the DARTEL deformation field (from individual space to MNI space) generated in VBM analyses.

(c) The merged deformation field was used to register the individual diffusion data into the MNI space, or vice versa.

(5) Deterministic fiber tracking

The HARDI/Q-Ball reconstruction method implemented in the Diffusion toolkit 0.6.4.1 (http://www.trackvis.org) was used for the deterministic fiber tracking to reconstruct the whole brain fibers with an angle threshold of 80 degree and an FA threshold of 0.1.

(6) Probabilistic fiber tracking (BEDPOSTX+PROBTRACKX)

A partial volume model (or ball-stick model) was applied to estimate the diffusion orientation distribution with the following parameters: maximum number of fibers per voxel = 3, burnin period = 1000 and number of iterations = 1250, deconvolution model = sticks with a range of diffusivities. This was realized using the bedpostx program in FSL 5.0.10. Then, probabilistic fiber tracking was performed using the probtrackx program with predefined seeds and the following parameters: number of samples = 5000, and angle threshold = 80 degree.

(7) Metric normalization

The diffusion metrics were normalized into the MNI space using the merged deformation field (BBR+DARTEL) and resampled into a cubic voxel of 2-mm.

(8) Generation of white matter skeleton

A revised TBSS pipeline was used to create the white matter skeleton. In contrast to the standard TBSS pipeline that directly and nonlinearly aligned the individual FA images to the averaged FA template (FMRIB-58) in MNI space using the FNIRT program, the revised pipeline co-registered the individual FA images using the merged deformation field (BBR+DARTEL). Then a mean FA image was created and a mean FA skeleton of the white matter was generated using the center-of-gravity method. Each subject's aligned FA images were then projected onto the mean FA skeleton by filling the mean FA skeleton with FA values from the nearest relevant tract center, which was achieved by searching perpendicular to the local skeleton structure for maximum value.

5.3 DKI

(1) Brain extraction (BET)

The non-brain tissues of the b = 0 images were removed by the brain extraction tool (BET) implemented in FSL.

(2) Motion and distortion correction (EDDY)

An “EDDY_OPENMP” program implemented in the FSL 5.0.10 was used to evaluate and repair image displacement and signal dropout caused by head motion, and image distortion caused by eddy current.

(3) Kurtosis metric calculation

The Marchenko Pastur primary component analysis (MPPCA) was used to reduce noise caused by high b-value in the preprocessed DKI data. The weighted linear least squares estimation algorithm was used to estimate the DKI parameters, which were used to calculate DKI metrics (both tensor and kurtosis metrics). The tensor metrics included the three eigenvalues, FA and MD. The kurtosis metrics included the radial (RK), axial (AK) and mean kurtosis (MK), and white matter microscopic metrics, such as the axonal water fraction (AWF), extra-axonal space (EAS) and intra-axonal

space (IAS). This step was carried out based on the Diffusion Kurtosis Imaging toolbox (https://github.com/NYU-DiffusionMRI/Diffusion-Kurtosis-Imaging)

(4) Spatial normalization estimation (BBR+DARTEL)

A two-step procedure was used to estimate the co-registration parameters between individual diffusion space and MNI standard space.

(a) Individual b = 0 images were aligned to the corresponding structural images using the BBR algorithm implemented in FSL.

(b) The BBR parameters were concatenated with the DARTEL deformation field (from individual space to MNI space) generated in VBM analyses.

(c) The merged deformation field was used to register the individual diffusion data into the MNI space, or vice versa.

(5) Metric normalization

The diffusion kurtosis metrics were normalized into the MNI space using the merged deformation field (BBR+DARTEL) and resampled into a cubic voxel of 2-mm. The normalized kurtosis metrics were also projected onto the white matter skeleton that generated in the DTI pipeline.

5.4 rs-fMRI

(1) Discarding unstable volumes

Five first functional volumes were discarded to allow signal to reach equilibrium and to ensure the participants to adapt to scanning noise. After deletion, all subjects have 175 time points.

(2) Slice timing correction

The remaining volumes were corrected for intra-volume temporal differences using sinc-interpolation.

(3) Head motion correction

Inter-volume head motion correction was performed by a six-parameter rigid-body transformation. Specifically, each volume was first realigned to the first volume and then realigned to the mean of these volumes after the first correction.

(4) Spatial normalization

To improve coregistration, non-brain tissues of the mean corrected functional and structural images were removed. Then, the mean corrected functional images were coregistered to the corresponding structural images using the BBR method. Finally, all motion-corrected functional volumes were spatially normalized to the standard MNI space using deformation fields derived from the VBM analysis and resampled to 3-mm isotropic voxels.

(5) Smoothing

For independent component analysis, the normalized fMRI data were smoothed with a FWHM of 8 mm. For other analyses, such as regional homogeneity (ReHo) and amplitude of low frequency fluctuation (ALFF), the smoothing procedure (with the same smoothing kernel) was performed after the measures were generated.

(6) Regressing out sources of noise

We regressed out several sources of variances, including the linear drift, Friston's 24 head motion parameter, signal from a region centered in the white matter and signal from the ventricular region. Notably, the regression of the global signal is still a matter of debate. Thus, both data with and without regressing out global signal were obtained in the data preprocessing.

(7) Scrubbing

We calculated frame-wise displacement (FD), indexing volume-to-volume changes in head position. These changes were obtained from derivatives of the rigid-body realignment estimates for fMRI data. The movement-contaminated time points were defined by $FD_{Jenkinson} > 0.5$ mm in this study. Then, the contaminated volumes, as well as 1 forward and 2 backward from these volumes, were deleted and imputed using cubic spline interpolation. If the number of imputed volumes was more than one third of the total length, the participant would be excluded from the study.

(8) Filtering

The temporal band-pass filtering (0.01-0.08 Hz) was performed on time series of each voxel to reduce the effects of low-frequency drift and high-frequency noise.

5.5 ASL

The ASL images were obtained using the spiral 3D FSE sequence, and the CBF maps were automatically generated by the scanner workstation immediately after the acquisition. Thus, here we did not include the steps for CBF calculation.

(1) Merge raw data (FSLMATHS)

The raw ASL images acquired by the spiral 3D-FSE sequence had good SNR but poor GM/WM contrast, while the ASL subtraction map had good GM/WM contrast but poor SNR. In order to integrate the advantages of these two types of images, we merged them using the following equation: merged map = (subtraction map × ASL map) / mean of all voxels in the subtraction map. The merged map was then used for later data processing.

(2) Skull stripping (BET)

The non-brain tissues in the merged map were stripped using the brain extraction tool (BET) implemented in FSL.

(3) Spatial normalization

A two-step procedure was used to estimate the co-registration parameters between individual CBF space and MNI standard space.

(a) Individual skull-stripped images were aligned to the corresponding structural images using the BBR algorithm implemented in FSL.

(b) The BBR parameters were concatenated with the DARTEL deformation field (from individual space to MNI space) generated in VBM analyses.

(c) The merged deformation field was used to register the individual CBF map into the MNI space and resampled into a cubic voxel of 2-mm.

(4) Scaling

Early studies had shown that CBF values are significantly influenced by labeling parameters and arterial circulation properties. In order to minimize the effect of the labeling variance across participants, the coregistered CBF map of each subject was further scaled by the demean scaling and z-score scaling methods.

(5) Smoothing

Each scaled CBF map was spatially smoothed with a Gaussian kernel of 6-mm

FWHM.

6. Quality control during analyses

(1) Reducing variations from centers and scanners

During analyses, the center and scanner factors should be controlled by adding covariates of no interest. We can also perform meta-analysis to better integrate results from different centers and scanners.

(2) Controlling for other confounding factors

Similarly, other disturbing factors such as head motion should also be controlled during the analyses.

7. Preliminary results

The first stage of quality control of the CHIMGEN data included 5819 participants and 4885 of them were genotyped. After excluding 23 participants with metal artefact, 1 with brain atrophy, 1 with excessively large ventricle and 41 with unqualified genetic data, the remaining 5753 participants (4844 with and 909 without genotyping data; 5753 participants with sMRI, DTI and rsfMRI data, 3619 with DKI data and 4108 with ASL data) were included in the quality control of neuroimaging data. The quality control results for neuroimaging data are shown in Figure 6.4.

In the 5753 participants, there were 5743 (99.83%) with qualified sMRI data, 5507 (95.72%) with qualified rs-fMRI data, and 5750 (99.95%) with qualified DTI. In the 3619 participants with DKI data, 3610 (99.75%) participants had qualified DKI data. In the 4108 participants with ASL data, 4108 (100%) participants had qualified ASL data.

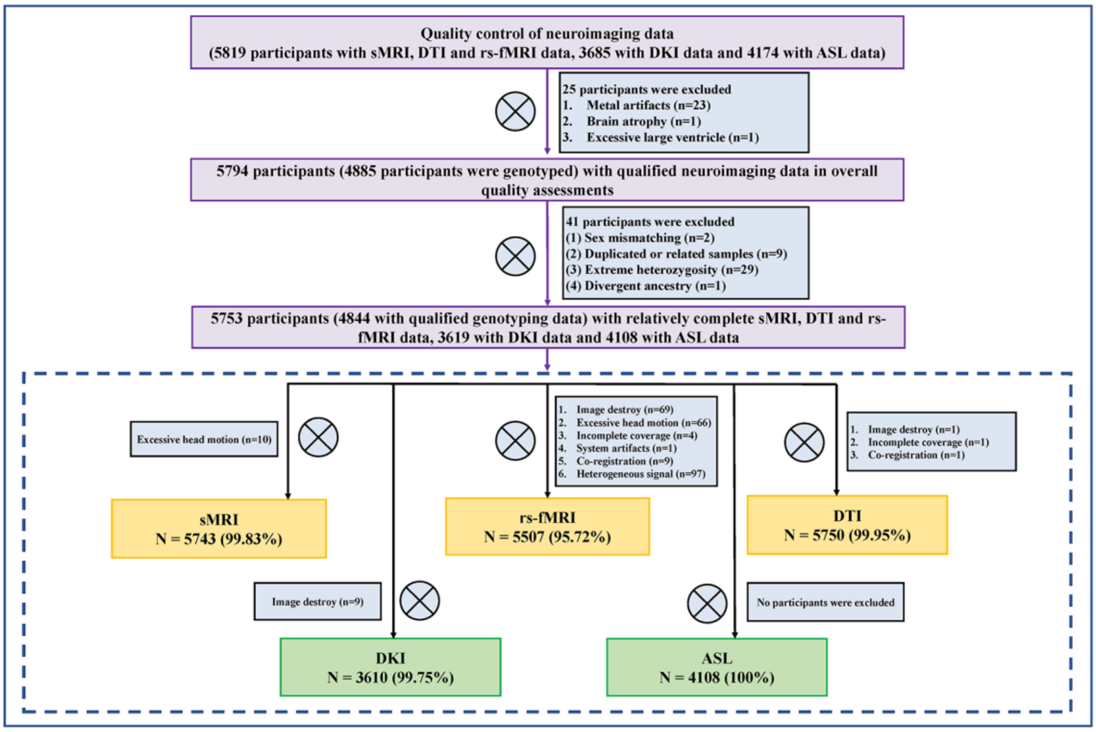

Figure 6.4 Quality control of MRI data
