# Supplementary material for: CHIMGEN: a Chinese imaging genetics cohort to enhance cross-ethnic and cross-geographic brain research
Source: Mol Psychiatry. 2019 Dec 11;25(3):517–29. doi: 10.1038/s41380-019-0627-6 (PMC7042768; doi:10.1038/s41380-019-0627-6)
Supplement: Supplementary file 4 [file 41380_2019_627_MOESM4_ESM.pdf]

DATA SHARING POLICY

The CHIMGEN consortium

Content

Chapter 1 Introduction…...……..…………………………………...……………2

Chapter 2 Principles for data sharing.…….………………………………...………3

Chapter 3 Available data resources...……………………………………....….…4

Chapter 4 Application and review....………………….……………………….………6

Chapter 5 Application form for collaboration..…….......…….….…………….………10

Chapter 6 Application form for summary data.………….….……………………..11

Chapter 7 Agreement for collaboration.……………….……………………….……………12

Chapter 8 Agreement for summary data...…………….……………………….……………13

Chapter 1 Introduction

The Chinese Imaging Genetics (CHIMGEN) study has an identifier of NCT02681822 on ClinicalTrials.gov. This study aims to identify genetic and environmental factors and their interactions that are associated with neuroimaging and behavioral phenotypes. This study was initiated in 2015 and has collected genomic, environmental, neuroimaging and behavioral data from over 7000 healthy Chinese Han participants aged between 18 and 30 years in 30 research centers located in 21 cities in the mainland of China. The CHIMGEN study is currently the largest available neuroimaging genetics cohort of non-Caucasian (Chinese Han) population. In particular, the environmental data also includes hundreds of quantitative natural and social environmental measures associated with the locations of each participant from birth to present derived from satellite images and national survey databases, which may facilitate the discovery of new environmental factors associated with the human brain. The CHIMGEN data will be open to bona fide researchers worldwide for scientific purposes.

The main objective of the Data Sharing Policy is to encourage extensive and appropriate use of the CHIMGEN data. All data sharing should comply with the laws and regulations of China at all times.

Chapter 2 Principles for data sharing

1. The CHIMGEN data will be accessible to all researchers for scientific purposes via different ways.
2. Applications for using the CHIMGEN data will be reviewed to ensure that research proposals have sufficient scientific significance and innovation, and are consistent with the ethical requirements and the consent of the participants.
3. Access to raw genetic data requires the permission from the Ministry of Science and Technology of the People´s Republic of China.
4. Applicants must sign a Data Use Agreement to ensure the anonymity and confidentiality of participants' data.
5. The CHIMGEN consortium has the ownership of the database, but will not claim over any inventions developed by researchers using the data.
6. For any publications using the CHIMGEN data, the phrase "for the CHIMGEN consortium*" should be included after the named authors. The asterisk referring to the following statement and list of names: "Data used in this article were obtained from the CHIMGEN study (http://chimgen.tmu.edu.cn). A list of the CHIMGEN investigators can be found at: http://chimgen.tmu.edu.cn/authorlist."
7. In the Method section of all published papers using the CHIMGEN data, researchers should include the following sentence to acknowledge data acquisition by the CHIMGEN consortium: "Data used in this article were obtained from the Chinese Imaging Genetics (CHIMGEN) study (http://chimgen.tmu.edu.cn)."
8. For each approved proposal, the CHIMGEN consortium will ask researchers to write a research summary within 2 weeks after approval and to timely report the status of the research (e.g. approved, in progress, completed, or published). The information will be shown on the CHIMGEN website.
9. Researchers should provide a copy of the published paper and/or a summary of other products developed using the CHIMGEN data to the consortium, which will be shown on the CHIMGEN website.
10. The CHIMGEN consortium reserves the right to amend the Data Sharing Policy when necessary.

Chapter 3 Available data resources

1. The CHIMGEN consortium will continuously update the available resources on the CHIMGEN website (http://chimgen.tmu.edu.cn), including the raw data, statistical summary data and higher-level scientific results.

2. The potential data types

2.1 Genetic data

(1) The whole genomic data after quality control, including DNA sequencing and genotyping data. Researchers can use the whole genomic data via collaboration with the CHIMGEN consortium. Transferring genetic data to other institutions is strictly prohibited unless the permission from the Ministry of Science and Technology of the People´s Republic of China is obtained.

(2) The statistical summary data of associations between genotypes and neuroimaging or behavioral phenotypes. Researchers can submit a formal application for accessing these summary data.

(3) The higher-level scientific results derived from the CHIMGEN data will be freely available to all researchers and no applications are required.

2.2 Environmental data

(1) The urbanization scores of individual participants are accessible via a formal application.

(2) The early life adversity data are accessible via collaboration with the CHIMGEN consortium as sensitive personal information of participants may be involved.

(3) The environmental measurements derived from remote sensing (i.e., satellite images) and national surveys are accessible via collaboration with the CHIMGEN consortium.

(4) The statistical summary data of associations between environmental factors and neuroimaging or behavioral phenotypes are accessible via a formal application.

(5) The higher-level scientific results derived from the CHIMGEN data will be freely available to all researchers and no applications are required.

2.3 Neuroimaging data

(1) The raw data of the structural MRI (sMRI), diffusion tensor imaging (DTI), resting-state functional MRI (rs-fMRI), diffusion kurtosis imaging (DKI), and arterial spin labelling (ASL) are accessible via collaboration with the CHIMGEN

consortium.

(2) The summary data for neuroimaging measures are accessible via a formal application. The neuroimaging measures include a number of metrics derived from the five imaging modalities, such as the cortical thickness, volume, surface area, cerebral blood flow, regional homogeneity, amplitude of low frequency fluctuation of each brain region, diffusion measures of critical white matter tracts, and functional connectivity between brain regions.

(3) The higher-level scientific results derived from the CHIMGEN data will be freely available to all researchers and no applications are required.

2.4 Cognitive data

(1) The cognitive assessments include verbal memory assessed by California verbal learning test (CVLT), spatial memory assessed by Rey-Osterrieth complex figure test, working memory assessed by N-back test, executive function assessed by Go/No-Go task, fair decision making assessed by ultimatum game, information processing speed assessed by symbol digit modalities test, and perspective taking assessed by ball tossing game.

(2) The raw data of cognitive assessments are accessible via collaboration with the CHIMGEN consortium.

(3) The statistical summary data for cognitive assessments are accessible via a formal application.

(4) The higher-level scientific results derived from the CHIMGEN data will be freely available to all researchers and no applications are required.

2.5 Other assessments

(1) Other assessments include state and trait anxiety inventory, Beck depression inventory, and tridimensional personality questionnaire.

(2) These data are accessible via a formal application.

2.6 Demographic data

(1) Demographic data include age, sex, handedness, height, weight, etc.

(2) These data are accessible via a formal application.

Chapter 4 Application and review

1. Application

The application process is entirely on-line via the CHIMGEN website, mainly including three stages:

1.1 Registration

To confirm the identity of each researcher who applies for using the CHIMGEN data. During registration, each researcher needs to provide the following information:

(1) The name, address, email and brief curriculum vitae.

(2) The department, address, telephone and web site of his/her institution.

(3) A list of their most representative peer-reviewed publications.

1.2 Application categories

1.2.1 Application for collaboration

To apply for the raw data which are accessible via collaboration with the CHIMGEN consortium, researchers need to fill in an *application form for collaboration* which requires the following information:

(1) Required data type and quantity;

(2) Names of all researchers;

(3) A summary (< 200 words) of the research project clearly stating the core scientific question to be answered;

(4) Scientific rationale including background, experimental details and design;

(5) A description of the feasibility;

(6) A proposed timetable of the planned study;

(7) Preferred ways of collaboration.

1.2.2 Application for accessing statistical summary data

To apply for statistical summary data, researchers need to fill in an *application form for summary data* which requires the following information:

(1) Required data type and quantity;

(2) Names of all researchers;

(3) A summary (< 200 words) of the research project clearly stating the core scientific question to be answered;

(4) Scientific rationale including background, experimental details and design;

(5) A description of the feasibility;

(6) A proposed timetable of the planned study.

1.3 Signing an agreement

(1) Agreement for collaboration

After approval, researchers should sign an agreement for collaboration.

(2) Data use agreement for statistical summary data

After approval, researchers should sign a data use agreement for accessing statistical summary data.

2. The review process

To ensure each application can be reviewed in a timely manner, the review process will be conducted in the following intended timeframe (although the need to seek further information on particular applications may lead to a prolonged process):

(1) Registration: 2 weeks to check identity.

(2) Application: 4 weeks to review and respond.

(3) Agreement: 2 weeks to check.

(4) Data release: 4 weeks to release.

2.1 Registration

The CHIMGEN consortium will check the identity and the scientific achievements of each applicant. When this is done, the applicant will be issued with a unique identifying number which will be used for subsequent applications.

2.2 Application

(1) The CHIMGEN consortium will conduct a series of standard checks and seek advice on particular applications as needed. Decision on each application will be made by the CHIMGEN Principal Investigator (or his representatives);

(2) If needed, the consortium will ask the applicant to provide further information;

(3) When the review is completed, a decision letter will be sent to the applicant;

(4) For approved applications, the applicant will be asked to sign an agreement;

(5) For declined applications, the decision letter will explain the reasons for rejection.

2.3 Agreement

(1) Once the application is approved, the applicant will be asked to sign an Agreement for Collaboration or a Data Use Agreement for accessing summary data.

(2) After the agreement is signed, actions will be taken according to the agreement.

3. Access to the data

3.1 For collaboration

(1) The CHIMGEN consortium will designate an investigator for each approved collaboration study. This investigator will help the applicant to access the data.

(2) All data are stored and will be processed and analyzed at the cloud platform of the National Supercomputer Center (NSCC) in Tianjin. Researchers can directly access the raw data or the preprocessed data via a VPN account that connects to NSCC server to process the data. Downloading the raw data to researchers' local computers is prohibited due to safety reasons.

(3) Most popular software for imaging or genetic data analyses have been installed at the NSCC cloud platform. If the NSCC cloud platform does not meet some specific computing needs of an approved study, researchers can provide the codes or software and the designated investigator will help to install the software on the computing platform.

(4) There will be a fee for using NSCC platform service, usually cheaper than the charges of commercial companies. Both sides of the collaboration (i.e., the designated investigator from the CHIMGEN consortium and the applicant) should share the expenses evenly. The charging standard of NSCC and the way of payment can be found at the CHIMGEN website: http://chimgen.tmu.edu.cn.

(5) Regular meetings will be held between the two sides of collaboration to ensure the progress of the approved study.

3.2 For summary data

(1) After approval, the applicant can access or download the summary data from our website with the applicant-unique ID.

(2) The CHIMGEN consortium will also designate an investigator to help with any particular need in accessing summary data.

4. Publications and products

4.1 Using the CHIMGEN data will generate publications or other products.

4.2 All publications derived from the CHIMGEN data should:

(1) Each publication should include the phrase "for the CHIMGEN consortium*" after the named authors. The asterisk referring to the following statement and list of names: "Data used in this article were obtained from the CHIMGEN study (http://chimgen.tmu.edu.cn). A list of CHIMGEN investigators can be found at: http://chimgen.tmu.edu.cn/authorlist."

(2) The Method section of all publications using the CHIMGEN data should include a

sentence to acknowledge data acquisition by the CHIMGEN consortium. "Data used in this article were obtained from the Chinese Imaging Genetics (CHIMGEN) study (http://chimgen.tmu.edu.cn)."

(3) The summary and full text should be provided for each publication that will be shown on the CHIMGEN website (http://chimgen.tmu.edu.cn/publications).

(4) All high-level scientific results derived from the CHIMGEN data should be made freely available to the public.

(5) Publications by collaboration should include the CHIMGEN investigators as co-authors (including co-first and co-corresponding authors).

5. Reviewers

5.1 The academic board of the CHIMGEN consortium will review all applications.

5.2 The Principal Investigator (Prof. Chunshui Yu) of the CHIMGEN study will make the final decision on each application based on the suggestions and information provided by the consortium academic board.

5.3 The critical considerations for a decision are:

(1) Scientific significance of the proposal;

(2) Innovation of the proposal;

(3) Feasibility of the proposal;

(4) Academic background of researchers;

(5) Ethnical considerations and consent of participants.

Chapter 5 Application form for collaboration

Chinese Imaging Genetics (CHIMGEN)

Application Form for Collaboration

1. General information

(1) Project title

(2) Lead investigator

(3) Email

(4) Institution

(5) Proposed starting and finishing dates

2. Project proposal

(1) Background and scientific significance (< 200 words)

(2) Aims (< 50 words)

(3) Main analyses (< 500 words)

(4) Feasibility (< 100 words)

(5) Resources and specialties for supporting collaboration (< 200 words)

3. Required data type and quantity

(1) Genetic data

(2) Environmental data

(3) Neuroimaging data

(4) Cognitive data

(5) Other assessments

(6) Demographic data

4. Collaboration approaches

(1) Applying a VPN account for on-line data processing

(2) Visiting the lab of TMU for data processing

(3) Providing codes or software to be installed on NSCC cloud platform and the analyses can be performed by the applicant or the designated investigator.

(4) Performing Meta-analysis

(5) Regular meetings

Chapter 6 Application form for summary data

Chinese Imaging Genetics (CHIMGEN)

Application Form for Statistical Summary Data

1. General information

(1) Project title

(2) Lead investigator

(3) Email

(4) Institution

(5) Proposed starting and finishing dates

2. Project proposal

(1) Background and scientific significance (< 200 words)

(2) Aims (< 50 words)

(3) Main analyses (< 500 words)

(4) Feasibility (< 100 words)

3. Required data type and quantity

(1) Genetic data

(2) Environmental data

(3) Neuroimaging data

(4) Cognitive data

(5) Other assessments

(6) Demographic data

Chapter 7 Agreement for collaboration

Chinese Imaging Genetics (CHIMGEN)

Agreement for Collaboration

I fully understand that the access to the raw or summary data of the CHIMGEN study is only for the purpose of scientific investigations and I agree to the following terms:

1. I am responsible to make sure that any member in my team who utilizes these data to comply with this agreement.
2. I will not attempt to identify the identity of participants or attempt to contact them.
3. I will not download any raw data unless permission is gained.
4. I will not transfer the VPN account to anyone who is not involved in the project.
5. I understand that any results derived from the collaboration should be shared by the two sides. Any publications derived from the collaboration should include main investigators of the two sides. The author list should be determined by the Applicant and the CHIMGEN Principal Investigator.
6. If I publish papers using CHIMGEN data, I agree to include a sentence to acknowledge data acquisition by the CHIMGEN consortium: "Data used in this article were obtained from the Chinese Imaging Genetics (CHIMGEN) study (http://chimgen.tmu.edu.cn)."
7. I will submit a summary and the full text of any publication using the CHIMGEN data to the CHIMGEN consortium.
8. I agree to provide all summary data from the collaboration to make them freely available after being published.
9. I agree to provide all scientific results of publications derived from the CHIMGEN data to researchers worldwide.
10. I understand that the expenses for using the NSCC cloud platform service are shared evenly by me and the designated investigator from the CHIMGEN consortium.
11. I understand that failure to abide by these rules will result in termination of the collaboration. In that situation, any results derived from the CHIMGEN data cannot be published by the Applicant or anyone in his/her team. Should this happen, we reserve the right to appeal to legal means to compensate for and minimize all resultant damages.

____Electronic Signature of User

Chapter 8 Agreement for summary data

Chinese Imaging Genetics (CHIMGEN)

Data Use Agreement for Statistical Summary Data

I fully understand that the access to statistical summary data of the CHIMGEN study is only for the purpose of scientific investigations and I agree to the following terms:

1. I am responsible to make sure that any member in my team who utilizes these data to comply with this agreement.
2. I will not attempt to identify the identity of participants or attempt to contact them.
3. I understand that any personal information linked to any individual participant or any raw data from which the summary data are generated is not available.
4. I will not disclose these data to others beyond the proposed project.
5. If I publish papers using CHIMGEN data, I agree to include the phrase "for the CHIMGEN consortium*" after the named authors. The asterisk referring to the following statement and list of names: "Data used in this article were obtained from the CHIMGEN study (http://chimgen.tmu.edu.cn). A list of CHIMGEN investigators can be found at: http://chimgen.tmu.edu.cn/authorlist."
6. If I publish papers using CHIMGEN data, I agree to include a sentence to acknowledge data acquisition by the CHIMGEN consortium. "Data used in this article were obtained from the Chinese Imaging Genetics (CHIMGEN) study (http://chimgen.tmu.edu.cn)."
7. I will submit a summary and the full text of any publication using the CHIMGEN data to the CHIMGEN consortium.
8. I agree to provide all scientific results derived from the CHIMGEN data to researchers worldwide.
9. I understand that failure to abide by these guidelines will result in termination of my access to the CHIMGEN data. In that situation, any results derived from the CHIMGEN data cannot be published by the Applicant or anyone in his/her team.

____Electronic Signature of User
