# Supplementary figures and images for: CHIMGEN: a Chinese imaging genetics cohort to enhance cross-ethnic and cross-geographic brain research
Source: Mol Psychiatry. 2019 Dec 11;25(3):517–29. doi: 10.1038/s41380-019-0627-6 (PMC7042768; doi:10.1038/s41380-019-0627-6)

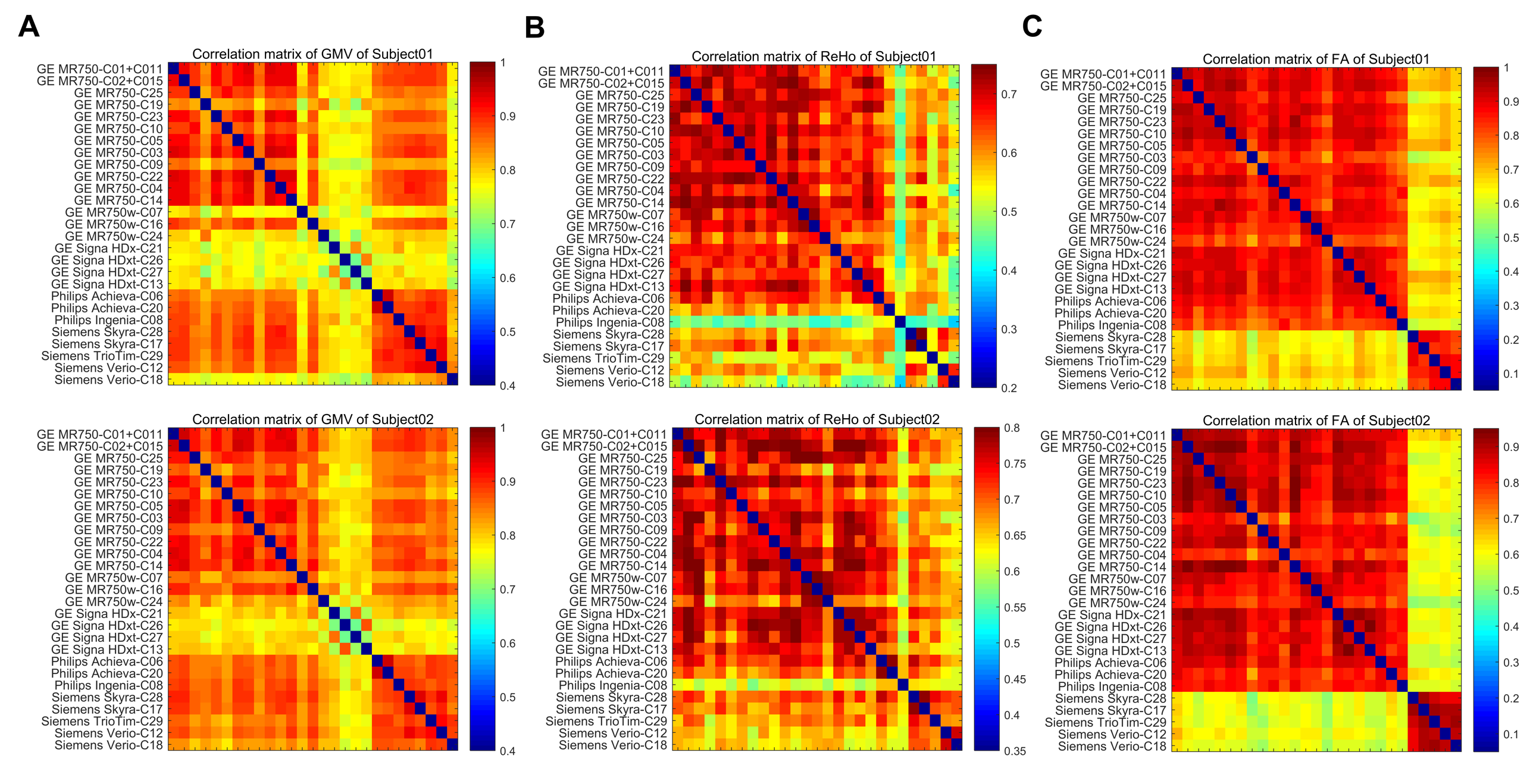

Supplement: Supplementary file 5 — Supplementary Figure 1 [file 41380_2019_627_MOESM5_ESM.tif]

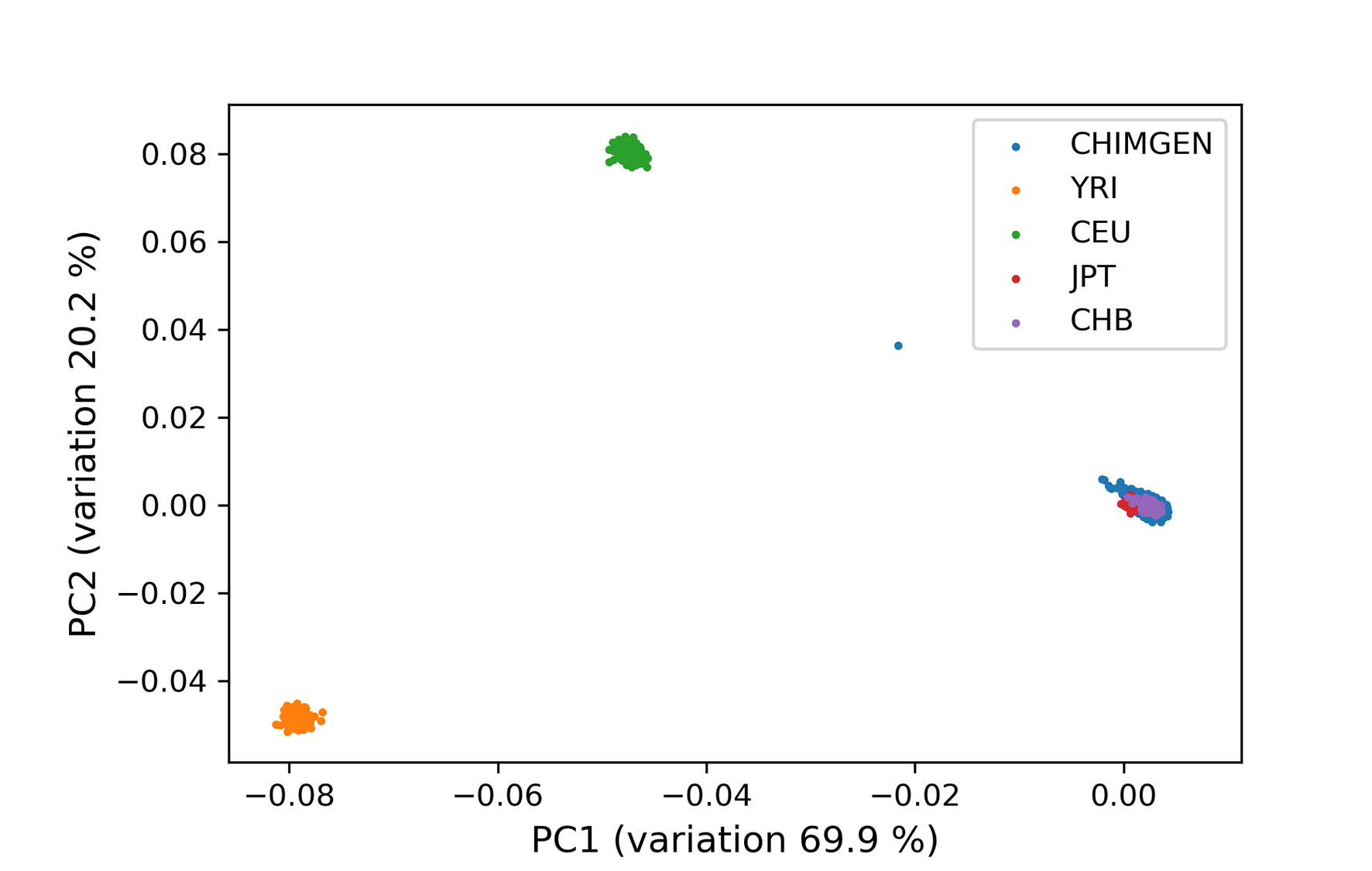

Supplement: Supplementary file 6 — Supplementary Figure 2 [file 41380_2019_627_MOESM6_ESM.tif]

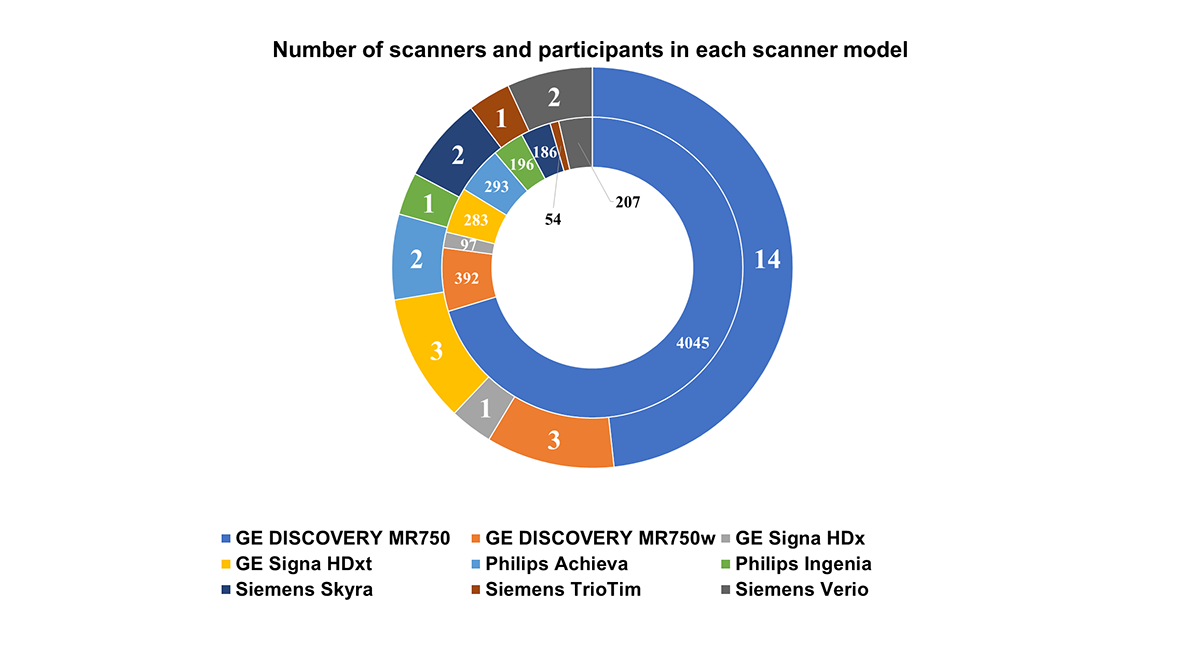

Supplement: Supplementary file 7 — Supplementary Figure 3 [file 41380_2019_627_MOESM7_ESM.tif]

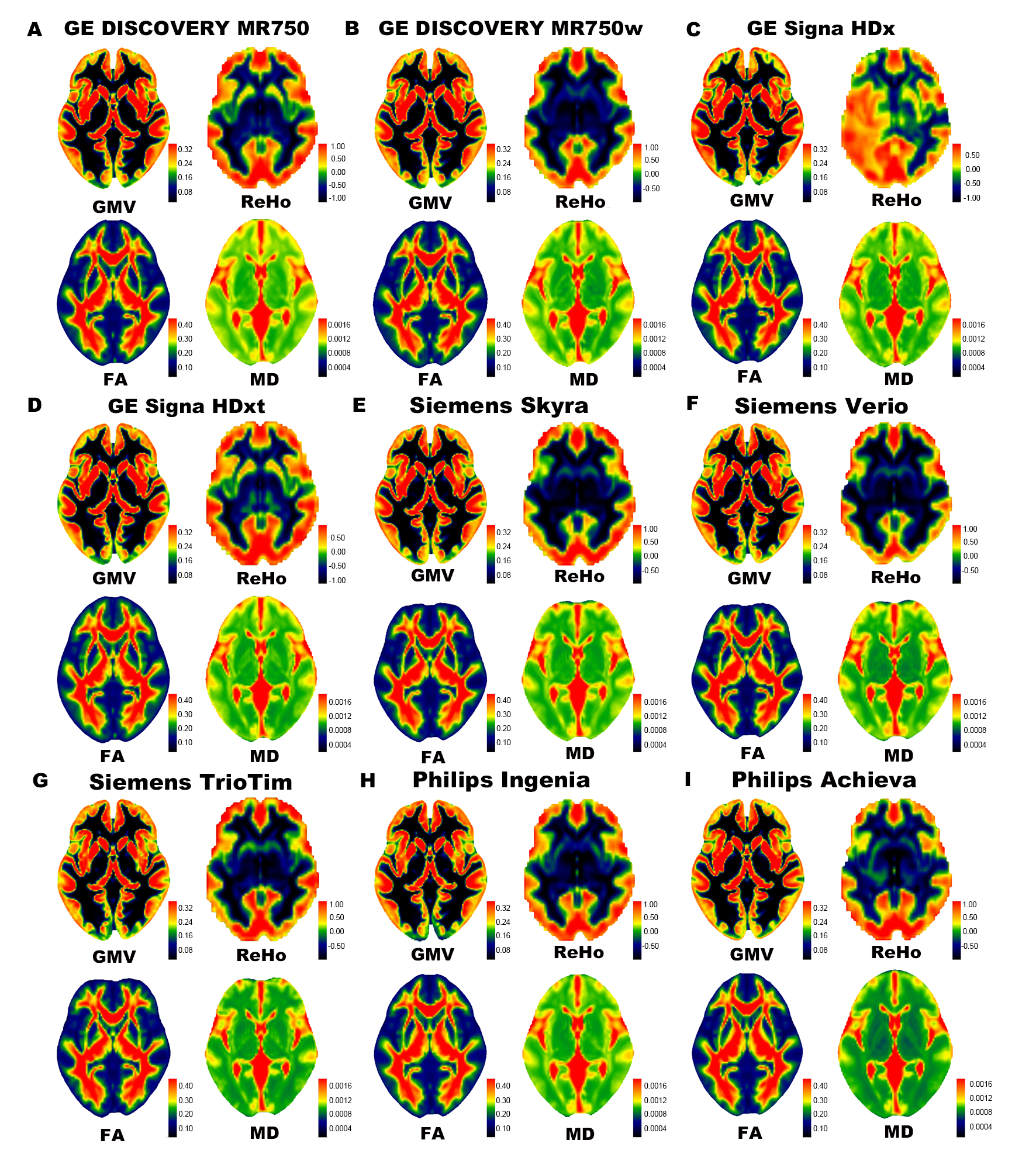

Supplement: Supplementary file 8 — Supplementary Figure 4 [file 41380_2019_627_MOESM8_ESM.tif]

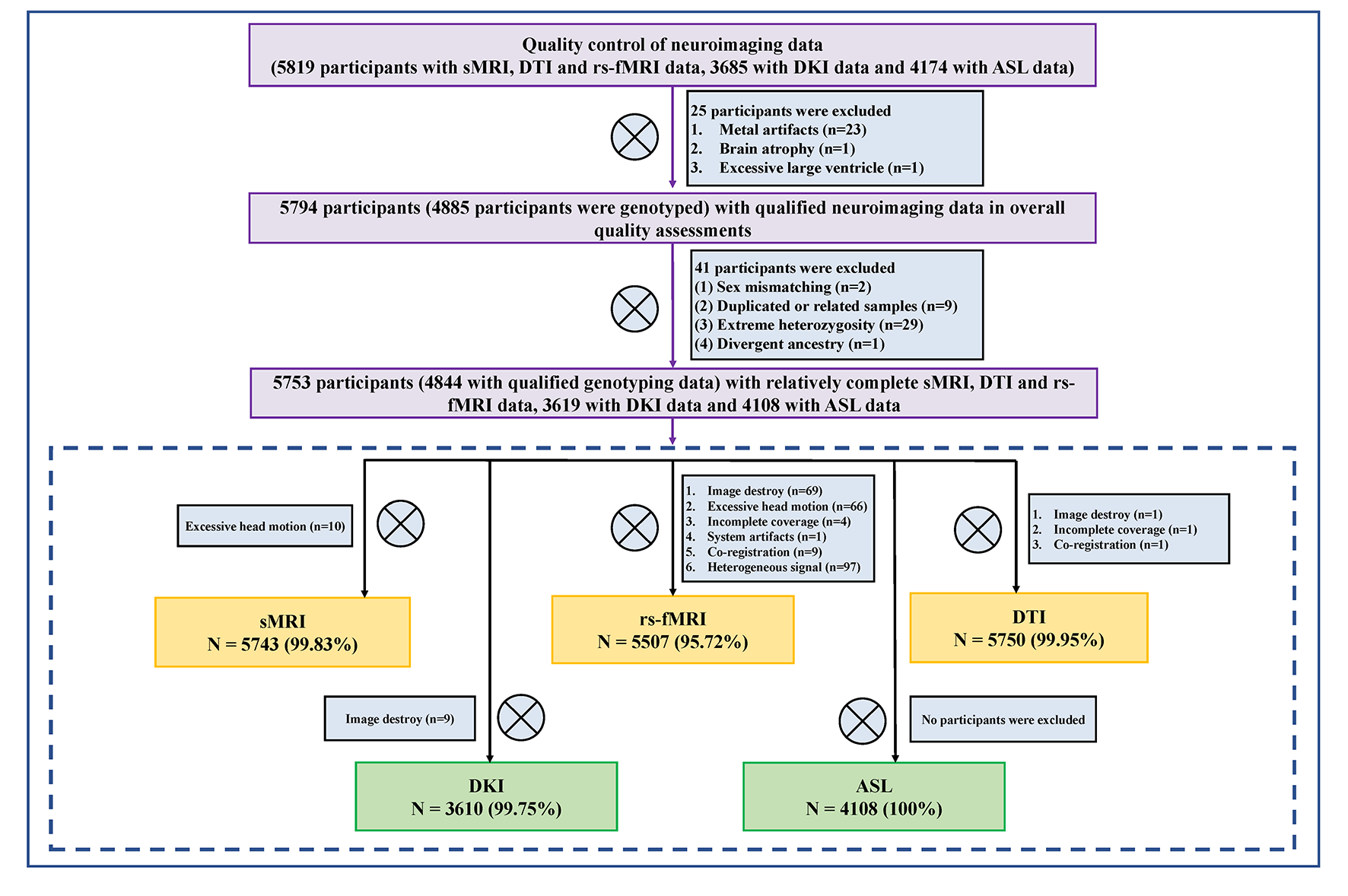

Supplement: Supplementary file 9 — Supplementary Figure 5 [file 41380_2019_627_MOESM9_ESM.tif]

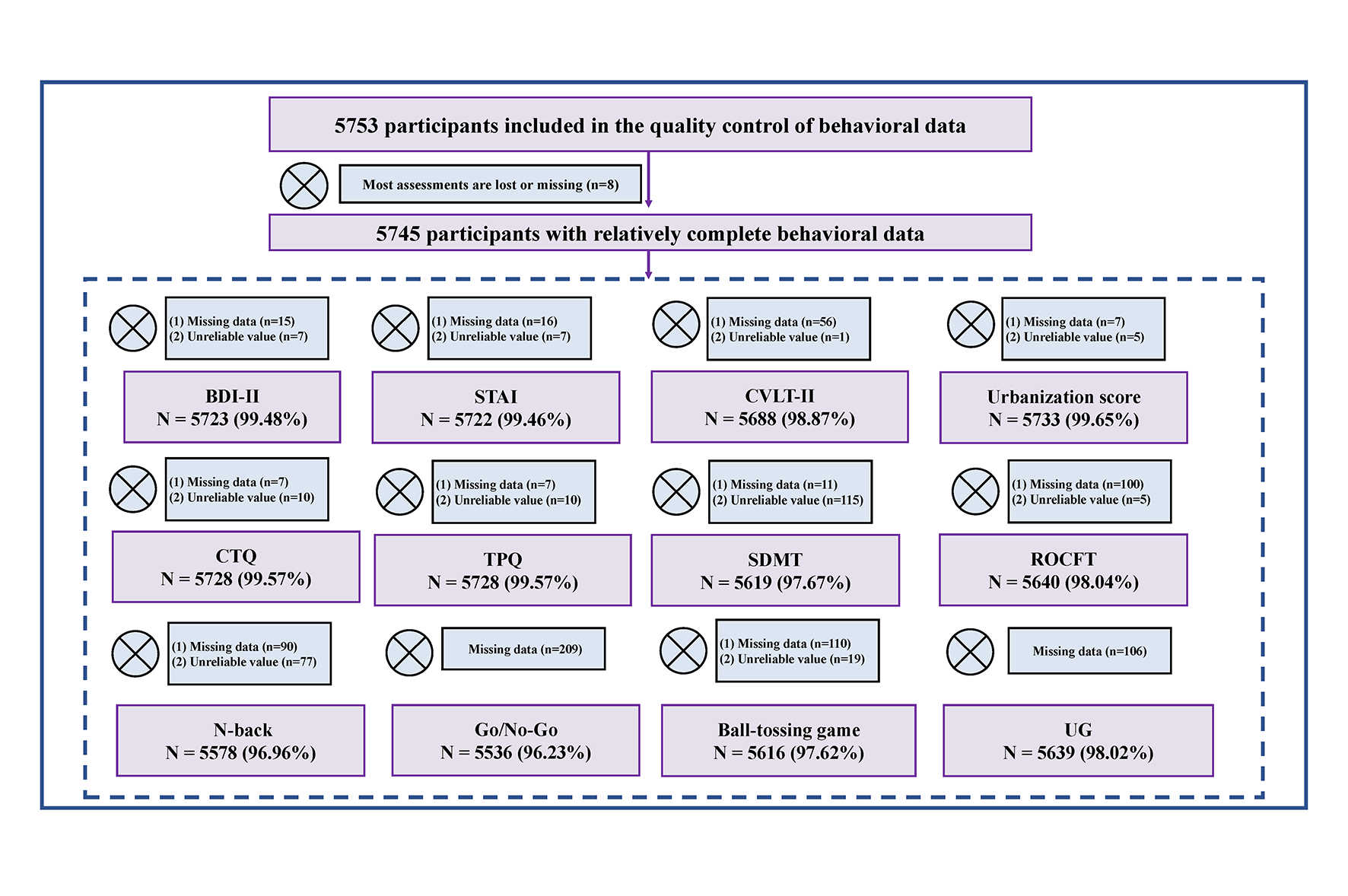

Supplement: Supplementary file 10 — Supplementary Figure 6 [file 41380_2019_627_MOESM10_ESM.tif]
