# Supplementary material for: CHIMGEN: a Chinese imaging genetics cohort to enhance cross-ethnic and cross-geographic brain research
Source: Mol Psychiatry. 2019 Dec 11;25(3):517–29. doi: 10.1038/s41380-019-0627-6 (PMC7042768; doi:10.1038/s41380-019-0627-6)
Supplement: Supplementary file 11 — Supplementary Figure Legends [file 41380_2019_627_MOESM11_ESM.docx]

**Supplementary Figure Legends**

**Supplementary Figure 1. Voxel-wise spatial correlation matrices of common MRI measures calculated from MRI data of two human volunteers acquired by different MR scanners (n = 27).**

Voxel-wise spatial correlation matrices are used to assess the consistency of GMV (A), ReHo (B), and FA (C) calculated from MRI data of subject 01 (upper row) and subject 02 (lower row) acquired by the 27 MR scanners. X and Y axes indicate the type of MR scanner used in each center. Color bar denotes the correlation coefficient. It should be noted that 27 MR scanners are used to acquire MRI data for the 29 centers because the MRI data of the center 11 are acquired in the center 1 and the MRI data of the center 15 are acquired in the center 2. FA, fractional anisotropy; GMV, grey matter volume; ReHo, regional homogeneity

**Supplementary Figure 2. Principal component analyses for identifying population stratification.** Only one participant is deviated from the population in population stratification analysis. CEU, Utah residents of Northern and Western European ancestry; CHB, Han Chinese in Beijing, China; CHIMGEN, Chinese Imaging Genetics; PC, Principle component; JPT, Japanese in Tokyo, Japan; YRI, Yoruba in Ibadan, Nigeria

**Supplementary Figure 3. Number of scanners and participants in each scanner model (n = 5753).** Inner circle indicates the number of participants in each scanner model. Outer circle indicates the number of scanners in each scanner model. GE, General Electrics

**Supplementary Figure 4. The mean parameter maps of each type of MRI scanner.** For each type of MRI scanner (A-I), the voxel-level maps of GMV calculated based on structural MRI data, ReHo calculated based on resting-state functional MRI data, and FA and MD calculated based on diffusion tensor imaging data averaged across all qualified participants are shown. All types of scanners showed similar and symmetrical spatial distribution of the GMV, FA and MD and 8/9 types of scanners showed similar and symmetrical spatial distribution of ReHo. However, the GE Signa HDx shows asymmetric spatial distribution of the ReHo map, especially in posterior brain regions (C). FA, fractional anisotropy; GMV, grey matter volume; MD, mean diffusivity; ReHo, regional homogeneity

**Supplementary Figure 5.** **Quality control of MRI data.** The diagram shows the streamline of quality control of MRI data. The reasons for exclusion, qualified number and rates are shown in corresponding box. ASL, arterial spin labelling; DKI diffusion kurtosis imaging; DTI, diffusion tensor imaging; rs-fMRI, resting-state functional MRI; sMRI, structural MRI

**Supplementary Figure 6. Quality control of behavioral assessments**. The diagram shows the streamline of quality control of behavioral assessments. The reasons for exclusion, qualified number and rates are shown in corresponding box. BDI-Ⅱ, Beck depression inventory-Ⅱ; CTQ, childhood trauma questionnaire; CVLT-Ⅱ, California verbal learning test-Ⅱ; ROCFT, Rey-Osterrieth complex figure test; SDMT, symbol digit modalities test; STAI, state and trait anxiety inventory; TPQ, tridimensional personality questionnaire; UG, ultimatum game
